# Are heritability and selection related to population size in nature? Meta‐analysis and conservation implications

**DOI:** 10.1111/eva.12375

**Published:** 2016-04-03

**Authors:** Jacquelyn L. A. Wood, Matthew C. Yates, Dylan J. Fraser

**Affiliations:** ^1^ Department of BiologyConcordia UniversityMontrealQCCanada; ^2^ Group for Interuniversity Research in Limnology and Aquatic Environment (GRIL)Université du Québec à Trois‐RivièresTrois‐RivièresQCCanada

**Keywords:** adaptation, biodiversity conservation, effective population size, evolution, habitat fragmentation, heritability, natural selection

## Abstract

It is widely thought that small populations should have less additive genetic variance and respond less efficiently to natural selection than large populations. Across taxa, we meta‐analytically quantified the relationship between adult census population size (*N*) and additive genetic variance (proxy: *h* ^2^) and found no reduction in *h* ^2^ with decreasing *N*; surveyed populations ranged from four to one million individuals (1735* h* ^2^ estimates, 146 populations, 83 species). In terms of adaptation, ecological conditions may systematically differ between populations of varying *N*; the magnitude of selection these populations experience may therefore also differ. We thus also meta‐analytically tested whether selection changes with *N* and found little evidence for systematic differences in the strength, direction or form of selection with *N* across different trait types and taxa (7344 selection estimates, 172 populations, 80 species). Collectively, our results (i) indirectly suggest that genetic drift neither overwhelms selection more in small than in large natural populations, nor weakens adaptive potential/*h* ^2^ in small populations, and (ii) imply that natural populations of varying sizes experience a variety of environmental conditions, without consistently differing habitat quality at small *N*. However, we caution that the data are currently insufficient to determine whether some small populations may retain adaptive potential definitively. Further study is required into (i) selection and genetic variation in completely isolated populations of known *N*, under‐represented taxonomic groups, and nongeneralist species, (ii) adaptive potential using multidimensional approaches and (iii) the nature of selective pressures for specific traits.

## Introduction

The management of small populations remains a major focus of conservation biology. Indeed, human‐induced habitat fragmentation has sufficiently depleted many species that they now only exist as small, isolated populations, and small adult census population size (*N*) is associated with elevated extinction risk (Lande 1988; Willi et al. 2006; Frankham et al. 2014). From a genetic‐evolutionary standpoint, small populations are theoretically expected to have reduced genetic variation underlying quantitative traits (additive genetic variance, *V*_A_) and be subject to stronger genetic drift (Lande 1988; Reed and Frankham 2003; Hoffmann and Sgrò 2011). Consequently, small populations are expected to have reduced adaptive potential wherein genetic drift is thought to overwhelm selection, with response to selection (which is linked to selection and *h* ^2^ through the breeder's equation *R* = *h* ^2^ *S*; Falconer and Mackay 1996) being less efficient than in large populations (Lande 1988; Ellstrand and Elam 1993; Falconer and Mackay 1996; Willi et al. 2006; Frankham et al. 2010). From a conservation standpoint, discerning the relationship between *N* and adaptive potential is important for prioritizing populations and for determining minimum *N* needed to deal with ongoing habitat fragmentation and future environmental change (Jamieson and Allendorf 2012; Frankham et al. 2013).

Laboratory studies have frequently supported that positive relationships exist between *N* and either quantitative genetic variation or response to selection (e.g. Wade et al. 1996; Swindell and Bouzat 2005; Bakker et al. 2010). However, the benign conditions typical of common garden experiments may not adequately represent stresses found in nature, particularly if these are related to *N*. These studies have also traditionally focused on a small number of species (e.g. *Drosophila*) so their conclusions might not necessarily apply to natural populations, specifically natural vertebrate populations, which can exhibit complex behaviours that might alter the relationship between *N* and genetic variation or response to selection. In fact, studies that have examined the relationship between quantitative genetic variation and *N* in natural populations have yielded no consensus, finding either greater or reduced heritability (*h* ^2^, a proxy for *V*_A_) in small relative to large populations (Widen and Andersson 1993; Waldmann 2001; Willi et al. 2006, 2007). In perhaps the most comprehensive study to date in terms of the number of populations and different traits assessed, no evidence was found for differences in *V*_A_ or *h* ^2^ from very small to large *N* in brook trout (Wood et al. 2015).

It is also rarely considered how the process of habitat fragmentation may alter selection, and hence possibly the response to selection via the breeder's equation, in addition to the genetic characteristics of populations as *N* is reduced (see Willi et al. 2007; Willi and Hoffmann 2012; Fraser et al. 2014; Wood et al. 2014, 2015). Broadly speaking, one might envision that ecological conditions differ between populations of varying *N* (Kawecki 2008) and so the magnitude of selection may also differ. A few empirical studies have provided equivocal support for this idea in natural populations, but had methodological issues such as reduced statistical power (Weber and Kolb 2013) or questionable proxies for *N* (density; Murúa et al. 2010). More generally, a clear conceptual and theoretical framework is currently lacking for predicting how habitat fragmentation affects selection *per se* as populations are reduced in size. Towards remedying this, we propose the following mutually nonexclusive hypotheses. These are intended as a reasonable point of departure for investigating how continuing fragmentation affects habitat conditions within and among fragments, and how this might consequently affect the relationships between *N* and *h* ^2^ or *N* and selection (or potential response to selection).

**Figure 1:**
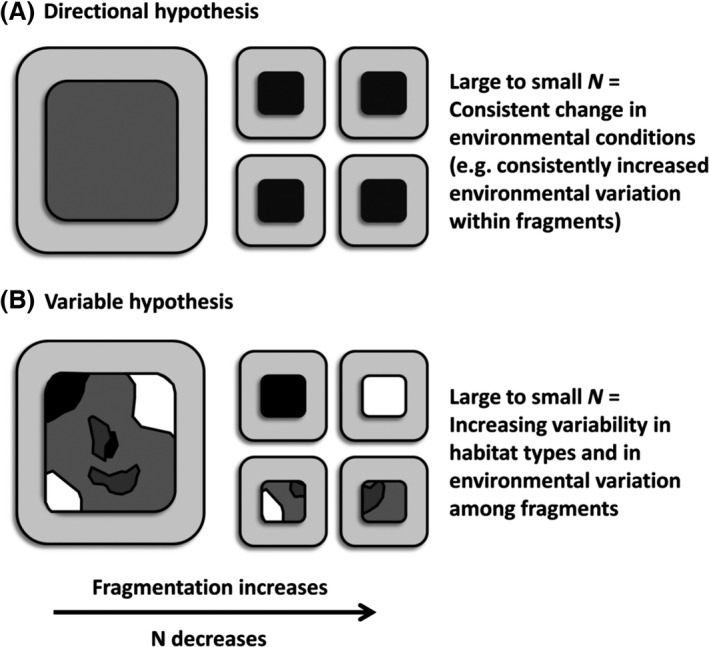
Two alternatives for the effect of habitat fragmentation on environmental conditions within and among fragments occupied by populations differing in *N*.

A first ‘Directional hypothesis’ is that habitat conditions shift in a persistent manner as fragment and population size are reduced during fragmentation (Fig. 1A; Willi and Hoffmann 2012; Wood et al. 2014). Conventionally, reduced gene flow, stronger genetic drift (and inbreeding depression) and reduced habitat quality in small populations (Willi et al. 2006; references therein) are the most likely net result. As a consequence, *h* ^2^ is reduced and the magnitude, direction or form of selection will consistently change across small relative to large populations and potentially also result in a systematic change in response to selection across an *N* gradient. This expectation might change somewhat for traits experiencing ongoing selection versus traits responding to novel selective factors. For example, ongoing balancing selection might maintain genetic variation for relevant genes even in small populations, thereby preserving adaptive potential (Turelli and Barton 2004), whereas under novel environmental conditions, selective response might depend on the amount or type of genetic variation present in the population (assuming trait independence). Under the Directional hypothesis, the point is that *in a consistent manner*,* h* ^2^ is reduced in the smaller, isolated populations experiencing persistent drift (Willi et al. 2006), and the magnitude, direction or form of selection change *in a consistent and directional manner*.

A possible alternative ‘Variable’ hypothesis is that habitat conditions become more variable as fragment and population size are reduced during fragmentation (Fig. 1B; Willi et al. 2006, 2007; Wood et al. 2014). From an ecological‐evolutionary standpoint, habitat fragments inhabited by small populations are considered to be simply random samples of larger, more complex fragments (e.g. Connor and McCoy 1979; Kotliar et al. 1999). Some of these small population fragments will thus typify the habitat heterogeneity or quality of the larger population fragments, while some will be more homogeneous and/or of poorer quality (Fraser et al. 2014; Wood et al. 2014). As a result, under the Variable hypothesis, *h* ^2^ and the magnitude of selection may be expected to be more variable among small than among large populations, as might the direction and form of selection. Put another way, some small populations will not necessarily exhibit or only partially exhibit the classic erosive attributes of the Directional hypothesis (e.g. reduced *h* ^2^ and a reduced response to selection).

Empirical evidence supporting these hypotheses is overall mixed but indicates that processes underlying both can operate simultaneously. For example, reduced *V*_A_, *h* ^2^ and selection are not always observed in small relative to large populations (e.g. Widen and Andersson 1993; Wood et al. 2015 versus Weber and Kolb 2013; Koskinen et al. 2002 versus Johansson et al. 2007). Evidence for more varying selection at various fitness traits was observed among small than among large populations of a plant and fish species, respectively (Willi et al. 2007; Fraser et al. 2014); yet in the fish, the extent of selection was also consistently higher in small than in large populations (Fraser et al. 2014).

We conducted a meta‐analytic review to investigate whether relationships exist between *N* and *h* ^2^ or *N* and selection (magnitude, direction, form) in natural populations, across taxa and a wide range of population sizes. Given the aforementioned points, we tested the following hypotheses: (i) *h* ^2^ decreases as *N* decreases; (ii) the magnitude, direction and form of selection differ consistently among populations of varying *N*; (iii) variability in *h* ^2^ increases as *N* decreases; and (iv) variability in the magnitude, direction and form of selection increases as *N* decreases. Support for hypotheses (i) and (ii) would be more consistent with the Directional hypothesis framework for explaining how habitat fragmentation affects the relationship between *N* and *h* ^2^ or *N* and selection; support for hypotheses (iii) and (iv) would be more consistent with the Variable hypothesis.

## Methods

### Quantitative review of primary literature

#### Heritability database

We collated *h* ^2^ estimates for natural populations of known *N* from the peer‐reviewed literature between 1980 and December 2014 using Google Scholar and one or more of the following key terms: *heritability*,* narrow‐sense heritability, quantitative genetic parameters*,* wild population* and *natural population*. We also searched the literature cited of previously published reviews (e.g. Geber and Griffen 2003; Carlson and Seamons 2008) and book chapters (e.g. Merila and Sheldon 2001; Postma 2014) for studies that met our criteria.

#### Selection database

To compile selection estimates acting on natural populations for which *N* data were also available, we surveyed the literature from 1984 to December 2014. Google Scholar was used to search within studies citing Lande and Arnold (1983) and the key terms: *natural population*,* wild population*,* population size*,* effective population size* and *breeding pairs*. Keyword searches of all Google Scholar articles were also conducted using various combinations of the terms: *phenotypic selection*,* natural selection*,* sexual selection*,* natural population*,* wild population*,* population size*,* effective population size*,* breeding pairs*,* selection coefficient*,* selection differential* and *selection gradient*. In assembling the database, selection studies included in earlier syntheses by Kingsolver et al. (2001) and Siepielski et al. (2009) were also reviewed and included where they met the necessary requirements. Finally, *N* databases provided in Leimu et al. (2006) and Palstra and Fraser (2012) were reviewed to determine whether any of the populations therein had also been investigated for selection.

### Criteria for inclusion in the database

#### Narrow‐sense heritability database

To be included in the *h* ^2^ database, studies must have estimated: (i) *h* ^2^ for quantitative traits in the study population(s) and (ii) *h* ^2^ for natural populations; studies of genetically manipulated populations were excluded. Following Carlson and Seamons (2008), we retained only those *h* ^2^ estimates generated from a breeding design which included half‐sibling families or from the regression of sire or mid‐parent phenotypic trait values on offspring trait values. Although the contribution of maternal effects to the covariance between mother and daughter is equal to the maternal genetic variance which may be quite small, estimates of *h* ^2^ based on dam‐offspring regressions were only included where this was the study's only method of *h* ^2^ estimation. This typically occurred when the traits being investigated were specific to females (e.g. laying date).

Occasionally, studies included in our database used multiple methodologies to generate several *h* ^2^ estimates from the same trait data. In such instances, we included only a single *h* ^2^ estimate in our analysis, prioritized based on the following order of estimation methods: animal model (REML), sibling analysis, mid‐parent–offspring regression and father–offspring regression. Estimates generated from animal models were prioritized first, as animal models are generally expected to yield more precise estimates of quantitative genetic parameters than other estimation methods for various reasons, including the ability to deal with unbalanced designs and account for various types of relationships (see Kruuk 2004; Kruuk and Hadfield 2007). Although low precision can be a problem in half‐sibling analyses, it can also provide a less biased *h* ^2^ estimate than parent–offspring regression if genotype‐by‐environment interactions (*G* × *E*) exist, so sibling analyses were prioritized secondarily. Finally, our database contained many studies of bird populations (133 of 249 studies) for which paternal care is common and might result in *h* ^2^ estimates inflated by paternal effects; we preferentially chose estimates derived from mid‐parent–offspring regression where both father–offspring and mid‐parent–offspring options were presented. Mittel et al. (2015) found that most *h* ^2^ methods yielded similar values; this suggests that even if methodologies had been prioritized in a different order, it would likely not have affected the outcome of our analysis.

Where *h* ^2^ was measured for the same population under different conditions, we used the estimate from the treatment that most closely reflected current conditions experienced by the population in nature or that represented an average or intermediate condition between putatively ‘ideal’ or ‘stressful’ treatments.

#### Selection gradient and differential database

We used similar inclusion criteria for selection as for the *h* ^2^ database. Following Kingsolver et al. (2001) and Siepielski et al. (2009), we only included studies that estimated selection using either standardized linear selection gradients (*β*), standardized quadratic selection gradients, standardized linear selection differentials (*s*), standardized quadratic selection differentials or any combination thereof (Lande and Arnold 1983; Arnold and Wade 1984a,b). These metrics estimate selection on a trait as the effect on relative fitness in units of phenotypic standard deviations, thereby allowing cross‐study comparisons of different populations, species and traits. Linear selection gradients estimate the strength of selection acting directly on a trait by removing the effects of selection from correlated traits included in the analysis, whereas selection differentials estimate total selection on the trait including indirect selection on other, correlated traits (Lande and Arnold 1983). Quadratic selection gradients and differentials estimate the curvature of the selection function. Stabilizing selection implies negative quadratic gradients and differentials, while disruptive selection implies positive values, although the observation of negative and positive values do not necessarily confirm stabilizing or disruptive selection (Kingsolver et al. 2001).

Some authors estimated selection in multiple years, but presented averaged data over the time period of their study. We attempted to contact these authors directly to obtain year‐specific selection coefficients, but these studies were included in the database irrespective of whether annual data were available. We also contacted authors where data were presented in figure format, and in several cases, we extracted selection coefficients using the Figure Calibration digitizing plugin (Rasband 2011) available for ImageJ (Abramoff et al. 2004).

### Population size data

Adult census population size, *N*, was used as the measure of population size in the analyses because only a small proportion of the total number of articles reviewed reported effective population size (*N*_e_), a caveat treated in the Discussion. Only a small proportion of *h* ^2^ or selection studies also reported estimates of *N*. For some studies lacking *N* data, estimates were obtained from other sources conducting work on the same population (other peer‐reviewed publications, government technical reports, etc.). Where *N* data could not be obtained from the original article or related sources, authors were contacted directly. Nineteen papers in the *h* ^2^ database and seven papers in the selection database contained *N* information in figures; in these instances, ImageJ was used to extract the relevant data digitally.

Over *t* generations, *N*_e_ in a fluctuating population is the harmonic mean of *N*_e_ and will be closest to the size of the generation with the smallest single generation *N*_e_ (Frankham 1995; Frankham et al. 2010). We therefore calculated the harmonic mean rather than arithmetic mean of *N* where selection was estimated in multiple years, but only a range of *N* across all years was provided (or for single‐year *h* ^2^ or selection estimates when only a range of *N* was available).

The *h* ^2^ and selection databases included many studies of colonially breeding or cavity nesting species of wild birds. The available *N* metric for most of these studies was the total number of breeding pairs in a given year, which we multiplied by two to approximate *N*. Across all taxa, for a few studies (18 of 167 populations in the *h* ^2^ database and 16 of 172 populations for the selection database), *N* was reported only as being greater than a certain value (more specific *N* data could not be obtained); here, the value itself was used as the estimate of *N*. For example, if *N* was estimated to be >100 000 individuals, 100 000 was reported in the database. This was likely justified where *N* was very large (>10 000, as for 8 and 5 populations in the *h* ^2^ and selection databases, respectively). Indeed, genetic diversity is sigmoidally related to *N*_e_ (Willi et al. 2006) so we did not expect much difference in the amount of genetic variation between populations that were 10 000 vs 20–30 000 individuals for example, or between those above a certain threshold population size. In the remaining cases (10 and 11 populations in the *h* ^2^ and selection databases), *N* was specified as being greater than some relatively low population size (e.g. *N *> 500). Although a disproportionately larger difference in genetic diversity is expected with incremental changes in *N* among small or medium sized populations compared with very large populations, these populations were retained in the analysis because their exclusion did not affect the results.

### Heritability and selection‐population size meta‐analysis data

For each retained study, we recorded the species name and taxonomic grouping (‘vertebrate’, ‘plant’ or ‘invertebrate’), common grouping (‘mammal’, ‘bird’, ‘fish’, etc.), *N*, trait class (‘morphological’ versus ‘life history’ versus ‘other’), *h* ^2^ estimate or selection coefficient, sample size, as well as the standard errors (SE) and *p*‐values when these data were available. For the *h* ^2^ database, we also recorded the statistical method used to determine whether this factor influenced estimates of *h* ^2^.

### Statistical analysis

In many database studies, SE were not reported; they were only available for 47% of *h* ^2^ estimates, 59% of linear selection gradients, 30% of linear selection differentials, 62% of quadratic selection gradients and 46% of quadratic selection differentials. Therefore, a formal meta‐analysis for the subset of *h* ^2^ and selection estimates with associated SE was conducted (hereafter, *Mh* ^2^ and *Msel,* respectively). An unweighted statistical subanalysis using the entire data set for *h* ^2^ and selection databases was also performed (hereafter, *UWh* ^2^ and *UWsel*, respectively).

One factor that we could not account for entirely in our analysis was the influence of gene flow on *h* ^2^ or selection; most studies lacked detailed information regarding immigration into researched populations. Bird populations were well represented in the *h* ^2^ data set and particularly epitomize this issue in their high capacity for dispersal. Therefore, we performed an additional subanalysis that implemented the same *h* ^2^ models but that excluded bird data (hereafter denoted *Mh* ^2^ *_no_bird* and *UWh* ^2^ *_no_bird*). In addition, *N* estimates for some bird populations may not always adequately reflect actual *N* (e.g. where they are based on the number of erected nest‐boxes); reported *N* might constitute only a portion of the population. Thus, we also conducted analyses of selection coefficient data both including and excluding bird populations (hereafter denoted *Msel_no_bird* and *UWsel_no_bird*, respectively).

#### Meta‐analysis: heritability

Although the distribution of *h* ^2^ is not well defined and the use of SE as an appropriate variance weight may be biased, it should still be possible to conduct a relatively conservative meta‐analysis using standard techniques (Ricklefs and Cadena 2008). For the formal meta‐analyses (*Mh* ^2^; *Mh* ^2^ *_no_bird*), *h* ^2^ estimates were therefore treated as if they followed a Gaussian distribution and were weighted according to the inverse of their variance as estimated from published SE (when available).

We evaluated the effect of *N* on *h* ^2^ using Bayesian mixed‐effects meta‐analyses implemented in the R package MCMCglmm (Hadfield 2010) in R (version 2.13.0; R Development Core Team 2011). We also conducted unweighted analyses (*UWh* ^2^; *UWh* ^2^_*no_bird*) using Bayesian mixed‐effect techniques. For all *h* ^2^ models, we used the default (weakly informative) priors. MCMC chains were run for 600 000 iterations with a burn period of 100 000 and thinning interval of 50; hence, parameters and associated confidence intervals (CI) were based on sampling the posterior distribution 10 000 times; model convergence and mixing were verified by visual examination of posterior traces and autocorrelation values.

Posterior modes for *h* ^2^ were calculated from models in which the mean‐centred logarithm of *N* was included as a continuous moderator variable, in addition to the categorical moderators: (i) trait class (‘morphology’, ‘life history’, ‘other’); and (ii) the type of analysis used to obtain the *h* ^2^ estimate (‘animal model (REML)’, ‘animal model (Bayesian)’, ‘parent–offspring regression’, ‘half‐sibling analysis’). Parent–offspring regression was not included in models using *Mh* ^2^ *_no_bird* or *UWh* ^2^_*no_bird* as these analysis types were no longer represented in the ‘other’ trait class. We approximated SE for *h* ^2^ estimates derived from Bayesian techniques as one half of the difference between the upper and lower CI divided by 1.96; these were included in the meta‐analyses.

Population and study were included as random effects to account for issues of autocorrelation. Variance structures were specified using the *idh* function which allowed the fitting of heterogeneous variances for the random effects and residuals; population and residual error variances were allowed to differ for each categorical moderator variable combination. However, study level variance was allowed to differ only for trait class, as most studies employed only a single analysis type.

In some cases, *h* ^2^ was estimated using genomic methods or from the regression of phenotypic similarity on the marker‐based coancestry (Ritland's regression; Ritland 1996); these only constituted a small proportion of the data set (8 of 249 total studies) so we excluded them from analyses.

#### Meta‐analysis: direction and form of selection

We evaluated the effect of *N* on the direction and form of selection using MCMCglmm as described for *h* ^2^. Posterior modes for each selection coefficient (linear gradients, quadratic gradients, linear differentials and quadratic differentials) were estimated from models in which the natural logarithm of *N* (mean centred) was specified as a continuous moderator variable. For linear and quadratic selection gradients and linear selection differentials, separate models were considered that contained an additional moderator variable of either trait class (morphology versus life history) or taxa (plants versus vertebrates; there were insufficient data available for invertebrates), and an interaction term of trait class or taxa with *N*. These additional variables were not included in the quadratic selection differential models as too few populations in some of the different moderator variable groups precluded confident assessments involving trait class or taxonomic interactions with *N*. We also could not evaluate more specific taxonomic groupings (mammal, fish, plants, etc.) in selection coefficient models due to insufficient data at this level. For *Msel_no_bird*, we were unable to investigate the *N *× taxa interaction for linear selection differentials as only plant data remained.

Study and population were included as random effects in all models to account for issues of autocorrelation. Heterogeneous variances were modelled for population and the residual variance between trait classes. For the taxon models, the residual variance was allowed to differ between vertebrates and plants.

#### Meta‐analysis: magnitude of selection

For linear selection coefficient data, not only the direction of selection but also the magnitude (absolute value) was of interest. Hence, a second set of models were performed in MCMCglmm which incorporated the folded normal distribution (Hereford et al. 2004; Kingsolver et al. 2012; Morrissey and Hadfield 2012).

To easily apply model outputs to a folded normal distribution, it was necessary to first discretize *N* by dividing populations into three or four bins, depending on the amount of data available for each type of selection coefficient (linear gradients; <100 individuals, 100–499, 500–999, and ≥1000 individuals, and linear differentials; <100, 100–999, and ≥1000 individuals). We designated our smallest bin as *N *<* *100 since, under stabilizing selection, loss of *V*_A_ might only become a serious issue at extremely small *N* (Willi et al. 2006). *N *<* *100 would correspond with *N*_e_ <10 applying the average ratio of *N*_e_/*N* of 0.1 reported by Frankham (1995); likewise *N *=* *100–499 might represent ‘typical’ small populations that would generally be considered vulnerable to loss of *V*_A_ (i.e. *N*_e_ <50 applying the 50/500 rule; Franklin 1980). At *N*_e_/*N *=* *0.1, populations with *N *=* *500–999 would be expected to have small‐to‐moderate *N*_e_, whereas *N *≥* *1000 populations would be much less likely to have small *N*_e_. Still, some extreme life‐history types (e.g. marine fishes) can generate extremely small *N*_e_/*N* ratios such that populations with *N *≥* *1000 individuals could still potentially have a small *N*_e_ and hence might be genetically similar to populations in the small size bins (Frankham 1995; Palstra and Ruzzante 2008; Palstra and Fraser 2012). Thus, for linear selection gradients where there was sufficient data in the largest bin, a separate set of analyses was conducted where the largest bin consisted of populations with *N *> 4000 individuals; this is close to the median, minimum viable population size that was found across species (median = 4169 individuals) in Traill et al. (2007).

Similar to the ‘direction and form of selection’ analysis, separate models were considered that contained trait class or taxa in conjunction with *N*, as well as a trait class or taxa by *N* interaction term. Interaction terms were included for linear selection gradients only, due to the limited data available for linear selection differentials. Study and population were included as random effects in all models. Heterogeneous variances were fitted for study, population and the residual variance across each factor‐level combination of trait class and *N* bin. For the taxon models, heterogeneous variances were specified for study and population for each *N* bin, while the residual variance was allowed to differ for each taxon × *N* bin combination. For the analysis using *Msel_no_bird*, sufficient linear gradient data were available to investigate the effect of *N* and its interaction with trait class only (*N *≥* *1000).

Model outputs (means and variances) for the selection coefficient data were then applied to the folded normal distribution to estimate the magnitude of selection associated with each *N* bin, trait class and taxon. Significance in differences between factor‐level combinations was assessed based on overlapping CI.

#### Variable hypothesis: heritability and selection

To investigate whether there was increased variability in *h* ^2^ or selection estimates at small *N* for different trait classes and taxa, we fitted an observation‐level random effect in the form of ‘*idh*(sqrt(1/ln(*N*))):units’. This fit a regression coefficient to each observation; these coefficients were drawn from a normal distribution with an estimated variance that decreases with increasing *N* (J. Hadfield; personal communication). This approach allows the exploration of variance in *h* ^2^ and selection along a continuous gradient of *N* while simultaneously accounting for uncertainty in *h* ^2^ and selection estimates. Standard errors for selection coefficients were negatively related to *N* (Appendix S1), but the relationships were positive between sample size and *N* (Appendix S2), together suggesting greater uncertainty in selection estimates for small populations; in an unweighted analysis, any observed increased heteroscedasticity at small *N* could be due to this potential bias. We therefore investigated variance in *h* ^2^ and selection with the inverse‐variance‐weighted data sets only (*Mh* ^2^ and *Msel*).

Model MCMC chains were run for 6 000 000 iterations with a burn period of 100 000 and thinning interval of 250 such that parameters and associated CI were based on effectively sampling the posterior distribution at least 5000 times; model convergence and mixing were verified by visual examination of posterior traces and autocorrelation values. For each model, we extracted the *N*‐related residual variance component and 95% CI at four different values for *N*: 50, 1000, 10 000 and 100 000. A minimum *N* of 50 was chosen because our database contained many populations with <100 individuals (18.6% of selection gradient estimates and 20% of *h* ^2^ estimates, for example); a maximum of 100 000 was chosen as a representative upper limit of *N*.

## Results

### Heritability data

Of 1106 *h* ^2^ studies reviewed, 249 met the criteria for inclusion in the database (Appendix S3). The full database included 3371 individual estimates of *h* ^2^ representing 167 different populations across 86 species in six different taxonomic groups. However, some studies estimated *h* ^2^ for the same data using multiple analysis methods or for both sexes; duplicate values were removed resulting in a final data set of 1735 *h* ^2^ estimates (146 populations and 83 species) which was used in all subsequent analyses (see Methods for removal criteria). The pared *h* ^2^ database included more estimates for vertebrates than plants (1134 vs 601 *h* ^2^ estimates, respectively), and more estimates for morphology (1136) than for either life‐history or other trait classes (446 and 153 *h* ^2^ estimates; Appendix S4).

### Selection data

Of over 2000 phenotypic selection studies reviewed, 133 met the criteria for inclusion in the database (see Appendix S5). Of these, 20 and 43 studies, respectively, overlapped with those in Kingsolver et al. (2001) and Siepielski et al. (2009); an additional four studies overlapped exclusively with Siepielski et al. (2013). Thus, our meta‐analysis found 66 additional studies with selection estimates (50% of studies and 32% of selection estimates, respectively). The database included 4293 records and 7344 individual estimates of selection across the four types of selection coefficients and represented 172 populations across 80 species in six different taxonomic groups (see Appendix S4). Most species included were widespread (88% of the total), generalist (89%) and diploid (81%). Overall, there were 44% more estimates of linear versus quadratic selection and 18% more estimates of selection gradients than selection differentials. As for *h* ^2^, the full database was biased towards estimates of selection for vertebrates (specifically for birds) than for plants or invertebrates, and there were also more estimates for morphology (4482 total selection estimates) than for other trait types (2862 estimates; Appendix S4).

### Heritability data: meta‐analysis and unweighted analysis

**Figure 2:**
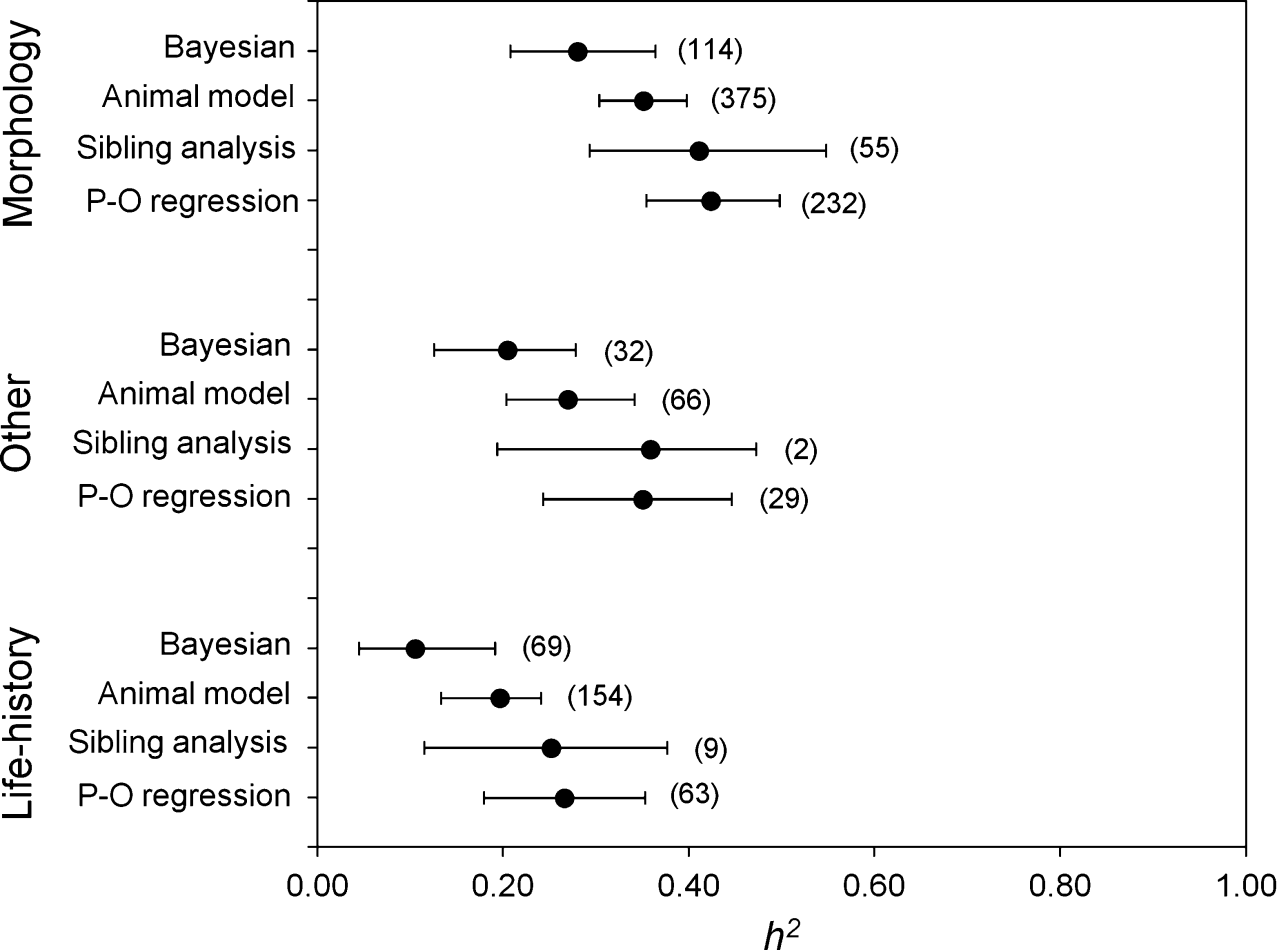
Posterior modes of weighted *h* ^2^ values estimated using four different methods of analysis within each of three different trait classes. Error bars represent 95% HPD confidence intervals calculated using MCMCglmm. Sample sizes in each category are in brackets.

There was no significant effect of *N* on *h* ^2^ using *Mh* ^2^ (Table 1). Morphological trait *h* ^2^ was consistently greater than for life‐history trait *h* ^2^ using *Mh* ^2^ (Fig. 2 and Table 1); *h* ^2^ for traits classified as ‘other’ was greater than *h* ^2^ for life‐history traits but did not differ from morphological traits. *UWh* ^2^ results were similar, except *h* ^2^ values for traits classified as ‘other’ no longer differed from life‐history traits (Appendix S6, Table F1 and G, Fig. G1).

**Table 1:** Results of meta‐analysis to investigate the effect of *N* on *h* ^2^ data using MCMCglmm. Models included *h* ^2^ data for bird populations

Trait class	Intercept	Fixed effect	Posterior mode	l–95% CI	u–95% CI	*P*_MCMC_
Life history (SE)	anova	(Intercept)	0.252	0.115	0.377	<0.001
		*N*	0.00116	−0.0100	0.0114	0.887
		Trait class (MO)	0.161	0.100	0.230	<0.001
		Trait class (O)	0.0859	0.000973	0.164	0.0417
		Analysis type (Bayesian)	−0.142	−0.278	0.00181	0.0610
		Analysis type (P‐O regression)	0.0151	−0.133	0.149	0.873
		Analysis type (REML)	−0.0750	−0.189	0.0678	0.329
	PO regression	(Intercept)	0.266	0.180	0.352	<0.001
		*N*	0.00222	−0.00987	0.0115	0.895
		Trait class (MO)	0.151	0.0970	0.227	<0.001
		Trait class (O)	0.0740	0.00407	0.167	0.0386
		Analysis type (anova)	−0.00940	−0.148	0.129	0.893
		Analysis type (Bayesian)	−0.145	−0.242	−0.0399	0.00560
		Analysis type (REML)	−0.0669	−0.154	0.00471	0.0658
	REML	(Intercept)	0.197	0.133	0.241	<0.001
		*N*	0.000948	−0.0106	0.0112	0.901
		Trait class (MO)	0.171	0.0982	0.229	<0.001
		Trait class (O)	0.0775	0.00573	0.167	0.0390
		Analysis type (anova)	0.0625	−0.0603	0.197	0.319
		Analysis type (Bayesian)	−0.0765	−0.140	0.00344	0.0624
		Analysis type (P‐O regression)	0.0703	−0.00201	0.157	0.0624
	Bayesian	(Intercept)	0.105	0.0451	0.191	0.00180
		*N*	0.00221	−0.00958	0.0118	0.894
		Trait class (MO)	0.150	0.101	0.234	<0.001
		Trait class (O)	0.0713	0.00569	0.165	0.0332
		Analysis type (anova)	0.142	−0.000724	0.282	0.0590
		Analysis type (P‐O regression)	0.154	0.0449	0.245	0.00460
		Analysis type (REML)	0.0697	−0.00570	0.140	0.0664
Morphology (SE)	anova	(Intercept)	0.411	0.293	0.548	<0.001
		*N*	0.000130	−0.0101	0.0112	0.893
		Trait class (LH)	−0.164	−0.229	−0.0976	<0.001
		Trait class (O)	−0.0791	−0.161	0.000695	0.0606
		Analysis type (Bayesian)	−0.136	−0.268	0.00994	0.0558
		Analysis type (P‐O regression)	0.0218	−0.128	0.155	0.881
		Analysis type (REML)	−0.0619	−0.201	0.0604	0.320
	PO regression	(Intercept)	0.424	0.354	0.497	<0.001
		*N*	0.00167	−0.0108	0.0110	0.902
		Trait class (LH)	−0.167	−0.231	−0.101	<0.001
		Trait class (O)	−0.0821	−0.157	−0.000584	0.0500
		Analysis type (anova)	0.0170	−0.150	0.131	0.897
		Analysis type (Bayesian)	−0.128	−0.243	−0.0441	0.00380
		Analysis type (REML)	−0.0704	−0.155	0.00121	0.0604
	REML	(Intercept)	0.351	0.303	0.398	<0.001
		*N*	0.000779	−0.0106	0.0110	0.895
		Trait class (LH)	−0.159	−0.232	−0.0991	0.000200
		Trait class (O)	−0.0805	−0.161	−0.000592	0.0552
		Analysis type (anova)	0.0763	−0.0628	0.194	0.323
		Analysis type (Bayesian)	−0.0611	−0.141	0.000104	0.0552
		Analysis type (P‐O regression)	0.0711	−0.00314	0.153	0.0594
	Bayesian	(Intercept)	0.280	0.207	0.364	<0.001
		*N*	0.00137	−0.0102	0.0114	0.900
		Trait class (LH)	−0.159	−0.229	−0.0985	<0.001
		Trait class (O)	−0.0757	−0.162	−0.00290	0.0550
		Analysis type (anova)	0.141	−0.00512	0.274	0.0560
		Analysis type (P‐O regression)	0.151	0.0435	0.244	0.00560
		Analysis type (REML)	0.0712	−0.00447	0.141	0.0638
Other (SE)	anova	(Intercept)	0.358	0.193	0.472	<0.001
		*N*	0.00160	−0.0102	0.0114	0.892
		Trait class (LH)	−0.0761	−0.165	−0.00304	0.0372
		Trait class (MO)	0.0771	0.000958	0.161	0.0548
		Analysis type (Bayesian)	−0.137	−0.273	0.00203	0.0498
		Analysis type (P‐O regression)	0.00402	−0.138	0.146	0.899
		Analysis type (REML)	−0.0562	−0.196	0.0597	0.303
	PO regression	(Intercept)	0.350	0.243	0.446	<0.001
		*N*	0.0000625	−0.00993	0.0115	0.899
		Trait class (LH)	−0.0835	−0.165	−0.00289	0.0364
		Trait class (MO)	0.0807	−0.000416	0.159	0.0538
		Analysis type (anova)	−0.0193	−0.155	0.133	0.896
		Analysis type (Bayesian)	−0.132	−0.245	−0.0442	0.00580
		Analysis type (REML)	−0.0674	−0.157	−0.00200	0.0564
	REML	(Intercept)	0.270	0.203	0.342	<0.001
		*N*	0.00119	−0.0101	0.0112	0.878
		Trait class (LH)	−0.0852	−0.166	−0.00203	0.0412
		Trait class (MO)	0.0842	−0.000664	0.160	0.0570
		Analysis type (anova)	0.0764	−0.0557	0.201	0.316
		Analysis type (Bayesian)	−0.0730	−0.141	0.00228	0.0610
		Analysis type (P‐O regression)	0.0686	−0.00525	0.154	0.0650
	Bayesian	(Intercept)	0.205	0.125	0.278	<0.001
		*N*	0.000827	−0.0103	0.0110	0.898
		Trait class (LH)	−0.0804	−0.168	−0.00769	0.0350
		Trait class (MO)	0.0664	0.000251	0.159	0.0522
		Analysis type (anova)	0.129	−0.0114	0.269	0.0632
		Analysis type (P‐O regression)	0.154	0.0460	0.246	0.00440
		Analysis type (REML)	0.0805	−0.00493	0.139	0.0616

Among the different methods used to estimate *h* ^2^ for the full data set, Bayesian animal models tended to produce lower *h* ^2^ estimates. Using *Mh* ^2^, Bayesian animal models produced significantly lower *h* ^2^ estimates relative to parent–offspring regression (Fig. 2 and Table 1) but did not differ from REML animal models. anova half‐sibling models tended to produce intermediate *h* ^2^, and these did not differ from REML animal models, parent–offspring regression or Bayesian animal models. REML‐derived estimates of *h* ^2^ were also similar to *h* ^2^ estimated using parent–offspring regression.

Using *UWh* ^2^, REML and Bayesian animal model techniques produced significantly lower *h* ^2^ values than *h* ^2^ estimates produced by parent–offspring regression (Appendix S6, Table F1 and G, Fig. G1). Heritability estimated by half‐sibling analysis was also significantly greater than produced by Bayesian animal models, but did not differ from *h* ^2^ generated by REML animal models. Heritability estimated from parent–offspring regressions and half‐sibling methods was similar.

Using *Mh* ^2^ *_no_bird* and *UWh* ^2^ *_no_bird*, no effect of *N* on *h* ^2^ was observed, nor were there any significant differences between any of the trait classes or analysis methods (Appendix S6, Table F2 and G, Figs. G2 and G3).

### Selection data: meta‐analysis and unweighted analysis

#### Direction of selection: linear gradients and differentials

**Figure 3:**
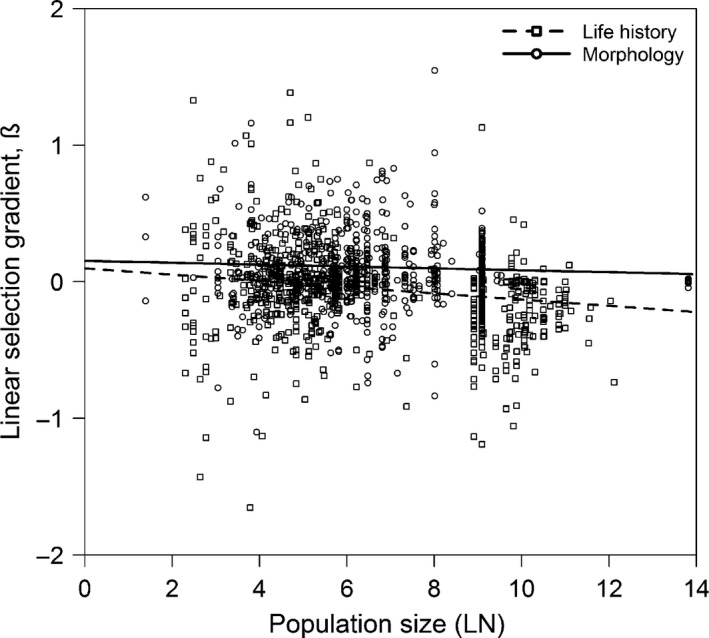
The relationship of weighted linear selection gradient values with (log‐transformed) *N*.

There was little evidence for a relationship between *N* and the direction of linear selection gradients using *Msel*, the selection coefficient with the most data (Table 2). The exception was that directional selection exhibited a weak negative relationship with *N* that was significant for life‐history traits but not for morphological traits (Fig. 3). Similar to Kingsolver et al. (2012), the direction of selection on morphological traits was positive overall. Across taxa, directional selection acting on plants tended to be shifted towards more positive values relative to vertebrates (Table 2). *UWsel* yielded similar results to *Msel* except that directional selection acting on life‐history traits was positive and significant and there was no longer an effect of *N* for life‐history traits (Appendix S8, Table H1).

**Table 2:** Results of meta‐analysis to investigate the effect of *N* on selection coefficient data using MCMCglmm. Models included selection coefficient data for bird populations

Selection coefficient	Intercept	Fixed effect	Posterior mode	l–95% CI	u–95% CI	*P*_MCMC_
Linear gradient (SE)	Life history	(Intercept)	−0.0411	−0.0881	0.0149	0.156
		*N*	−0.0214	−0.0410	−0.00443	0.0182
		Trait class (MO)	0.144	0.0786	0.220	<0.001
		*N *× trait class (MO)	0.0139	−0.00994	0.0439	0.236
	Morphology	(Intercept)	0.114	0.0633	0.161	<0.001
		*N*	−0.00812	−0.0268	0.0132	0.506
		Trait class (LH)	−0.153	−0.221	−0.0771	<0.001
		*N *× trait class (LH)	−0.0185	−0.0438	0.0102	0.238
	Plants	(Intercept)	0.116	0.0407	0.175	0.00150
		*N*	−0.0132	−0.0331	0.00851	0.230
		Taxa (V)	−0.0842	−0.165	−0.00596	0.0335
		*N *× taxa (V)	−0.00471	−0.0290	0.0254	0.890
	Vertebrates	(Intercept)	0.0240	−0.0223	0.0657	0.319
		*N*	−0.0159	−0.0321	0.00356	0.111
		Taxa (P)	0.0962	0.00872	0.169	0.0310
		*N *× taxa (P)	0.00173	−0.0261	0.0287	0.896
Linear differential (SE)	Life history	(Intercept)	−0.0951	−0.222	0.0518	0.181
		*N*	−0.0429	−0.0844	0.0263	0.267
		Trait class (MO)	0.246	0.0578	0.43127	0.0124
		*N *× trait class (MO)	0.0183	−0.0430	0.0869	0.498
	Morphology	(Intercept)	0.161	0.0245	0.292	0.0174
		*N*	−0.0101	−0.0405	0.0253	0.604
		Trait class (LH)	−0.260	−0.435	−0.0609	0.0088
		*N *× trait class (LH)	−0.0255	−0.0844	0.0469	0.492
	Plants	(Intercept)	0.191	0.0319	0.373	0.0218
		*N*	−0.0141	−0.0573	0.0206	0.346
		Taxa (V)	−0.208	−0.430	0.000499	0.0600
		*N *× taxa (V)	0.0259	−0.0377	0.0774	0.463
	Vertebrates	(Intercept)	−0.00199	−0.127	0.127	0.972
		*N*	0.00098	−0.0396	0.0462	0.901
		Taxa (P)	0.196	−0.00014	0.414	0.0524
		*N *× taxa (P)	−0.0315	−0.0794	0.0364	0.476
Quadratic gradient (SE)	Life history	(Intercept)	0.0290	−0.109	0.170	0.673
		*N*	0.0369	−0.00363	0.0822	0.0718
		Trait class (MO)	−0.0391	−0.174	0.111	0.648
		*N *× trait class (MO)	−0.0255	−0.0754	0.0184	0.204
	Morphology	(Intercept)	−0.000500	−0.0335	0.0295	0.861
		*N*	0.00756	−0.00891	0.0271	0.310
		Trait class (LH)	0.0201	−0.110	0.175	0.644
		*N *× trait class (LH)	0.0349	−0.0179	0.0739	0.197
	Plants	(Intercept)	0.00897	−0.0726	0.106	0.694
		*N*	0.0716	0.0232	0.107	0.0026
		Taxa (V)	−0.0375	−0.131	0.0583	0.429
		*N *× taxa (V)	−0.0567	−0.102	−0.0146	0.00900
	Vertebrates	(Intercept)	−0.0176	−0.0524	0.0129	0.213
		*N*	0.00415	−0.00616	0.0210	0.297
		Taxa (P)	0.0487	−0.0542	0.130	0.415
		*N *× taxa (P)	0.0595	0.0171	0.103	0.0094
Quadratic differential (SE)		Intercept	−0.0120	−0.0646	0.0440	0.733
		*N*	0.00927	−0.0112	0.0436	0.231

*Msel* results for linear selection differentials were similar to those for linear gradients (Table 2). Morphological traits experienced positive selection that differed significantly from selection acting on life history; life‐history directional selection was not significant overall. *N* had no effect on the direction of selection for either trait class, and directional selection also did not change with increasing *N* for either plants or vertebrates. *UWsel* differed from *Msel* in that linear differential values were similar for the two trait classes and differed significantly from zero for vertebrates but not for plants (Appendix S8, Table H1). Using *Msel_no_bird* and *UWsel_no_bird*, the results were concordant with *Msel* and *UWsel* in the majority of cases (Appendix S8, Table H2).

#### Form of selection: quadratic gradients and differentials

*N* had no effect on the form of selection as measured by quadratic selection differentials and did not influence quadratic selection gradients for either life‐history or morphological traits using *Msel* (Table 2). Overall, quadratic selection gradient estimates for life‐history and morphological traits did not differ from zero, similar to that observed in Kingsolver et al. (2012). Changing *N* did have a different effect on quadratic gradient values between plants and vertebrates; *N* had no effect on quadratic gradients for vertebrates but a significant, positive effect in plants (Table 2). *UWsel* yielded similar results to *Msel* except that the positive effect of *N* on quadratic gradients for plants was no longer significant (Appendix S8, Table H1). Results using *Msel_no_bird* and *UWsel_no_bird* were similar to *Msel* and *UWsel* (Appendix S8, Table H2).

#### Magnitude of selection: linear gradients and differentials

**Figure 4:**
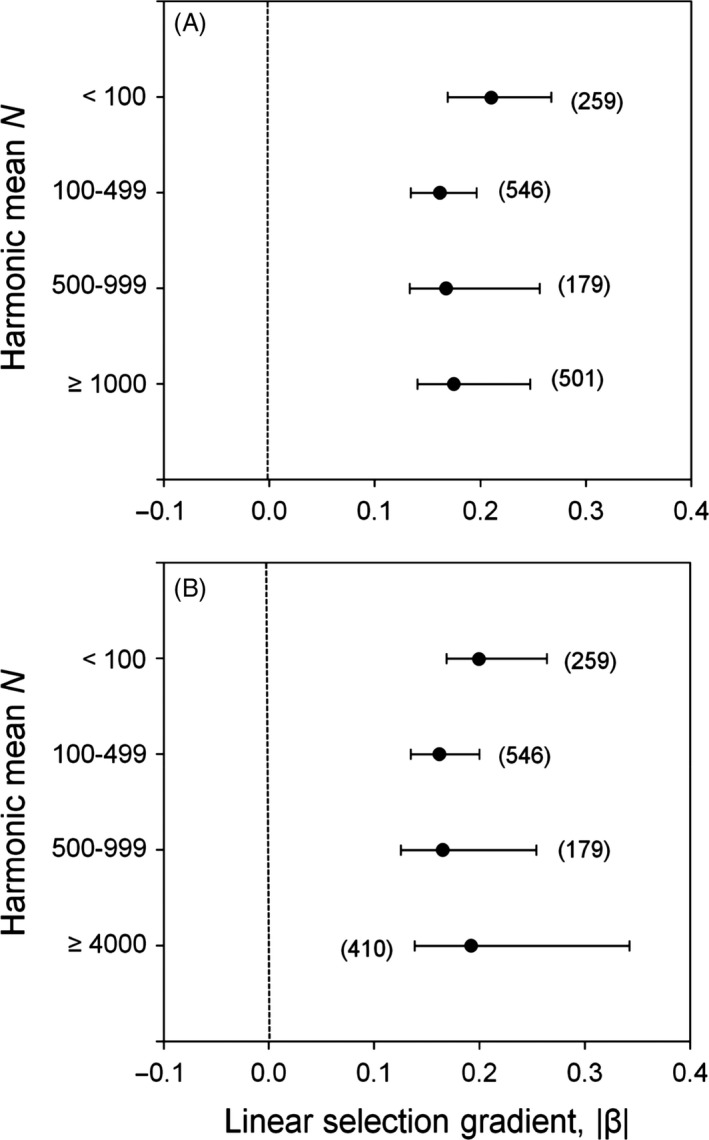
Posterior modes of the weighted magnitude of linear selection gradients for four different *N* bins where the largest bin consisted of (A) *N *≥* *1000 individuals or (B) ≥4000 individuals. The magnitude of selection was calculated using the folded normal distribution. Error bars represent 95% HPD confidence intervals calculated using MCMCglmm. Sample sizes in each *N* bin are in brackets.

**Figure 5:**
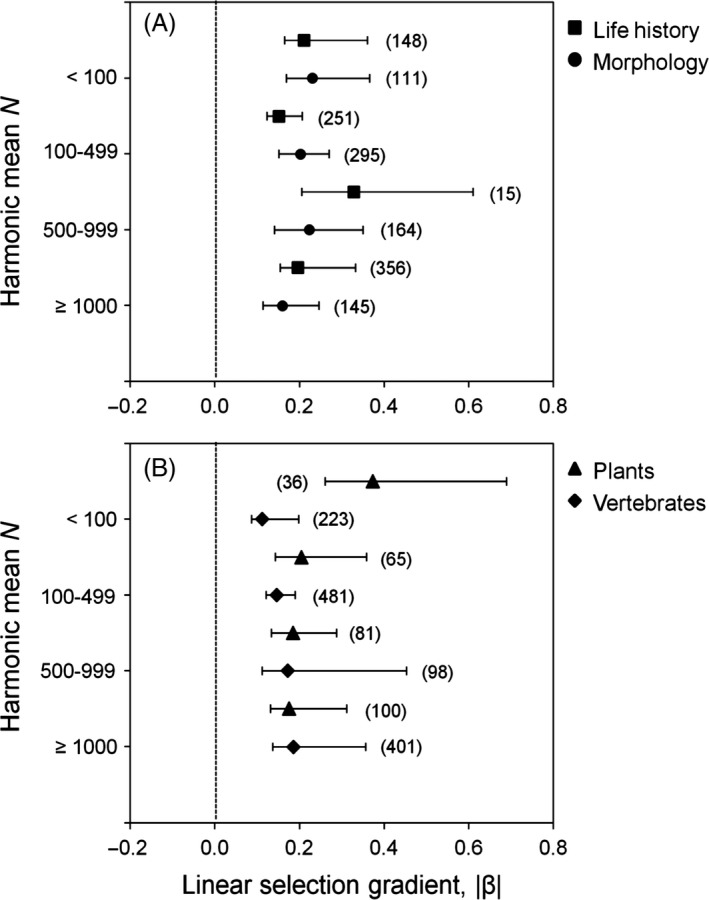
Posterior modes of the weighted magnitude of linear selection gradients for (A) morphological and life‐history traits and (B) plants and vertebrates in each of four *N* bins. The magnitude of selection was calculated using the folded normal distribution. Error bars represent 95% HPD confidence intervals calculated using MCMCglmm. Sample sizes in each *N* bin are in brackets.

No relationship between *N* and the magnitude of linear selection gradients was found using *Msel* (Fig. 4). highest posterior density (HPD) CI were overlapping for all *N* bins, including when the largest bin contained only populations of *N *>* *4000 suggesting no significant difference in the strength of selection (Fig. 4). The magnitude of selection was also similar across *N* bins within each trait class and taxonomic group, and between trait classes and taxa within each *N* bin (Fig. 5); the sole exception was for greater selection acting on plants than vertebrates in the smallest size bin (Fig. 5). Results for linear selection differentials revealed similar trends as for linear gradients; there was no difference in the magnitude of linear differential values in relation to *N* (Appendix S9, Fig. I1).

Results using *UWsel* were similar to *Msel*, with no differences observed in the magnitude of linear selection gradients or differentials relative to *N* including for different traits and taxa (linear gradients only) (Appendix S9, Fig. I1 and I2); however in general, *UWsel* point estimates for the magnitude of selection were higher. The magnitude of *UWsel* linear gradient values differed between different trait classes and taxa in only one instance (morphology versus life history, 100–499 *N* bin; Appendix S9, Fig. I3). *Msel_no_bird* and *UWsel_no_bird* yielded similar results to *Msel* and *UWsel* in most cases (Appendix S9, Figs. I4–I6).

#### Variable hypothesis: heritability and selection

For most trait classes and taxa, there was no evidence that variance in *h* ^2^ decreased with increasing *N*; residual variance CI overlapped for all reference *N* in most cases, with two exceptions: *h* ^2^ was more variable at small (*N *=* *50) than large *N* (*N *=* *10 000 and 100 000) for morphological traits, and also at *N *=* *50 than *N *=* *100 000 for vertebrates (Table 3).

**Table 3:** Results of meta‐analysis to test for increased variance in *h* ^2^ (±95% HPD confidence intervals) with decreasing *N* for different subsets of the *h* ^2^ database

	*N *=* *50	*N *=* *1000	*N *=* *10 000	*N *=* *100 000
All	0.0148 (0.0103, 0.0180)	0.0091 (0.0076, 0.0116)	0.0079 (0.0059, 0.0105)	0.0061 (0.0049, 0.0101)
Life history	0.0050 (0.0023, 0.0093)	0.0042 (0.0021, 0.0075)	0.0040 (0.0018, 0.0071)	0.0034 (0.0016, 0.0068)
Morphology	0.0163 (0.0124, 0.0214)	0.0101 (0.0081, 0.0130)	0.0082 (0.0062, 0.0108)	0.0068 (0.0051, 0.0096)
Other	0.0035 (0.0006, 0.0151)	0.0020 (0.0004, 0.0131)	0.0018 (0.0004, 0.0123)	0.0018 (0.0004, 0.0123)
Plants	0.0198 (0.0137, 0.0312)	0.0176 (0.0122, 0.0267)	0.0169 (0.0104, 0.0260)	0.0164 (0.0091, 0.0260)
Vertebrates	0.0110 (0.0078, 0.0148)	0.0073 (0.0054, 0.0093)	0.0058 (0.0042, 0.0081)	0.0047 (0.0035, 0.0075)

For selection coefficients, weak evidence for heteroscedasticity was found; differences in *N*‐associated residual variance estimates were more often significant than for *h* ^2^, although CI were often nonoverlapping for only the smallest and two largest reference *N*. For linear selection gradients, variance in selection was greater at small *N* than large *N* for both trait classes (Table 4, particularly morphology) and also for plants, but was similar across *N* for vertebrates. Linear selection differentials exhibited a similar pattern except that variance in selection was homogeneous across *N* for morphology (Table 4).

**Table 4:** Results of meta‐analysis to test for increased variance in selection coefficients (±95% HPD confidence intervals) with decreasing *N* for different subsets of the selection database

	*N*	Linear gradients	Linear differentials	Quadratic gradients	Quadratic differentials
All	50	0.0321 (0.0262, 0.0375)	0.0413 (0.0315, 0.0504)	0.0529 (0.0453, 0.0613)	0.0113 (0.0072, 0.0170)
	1000	0.0206 (0.0186, 0.0234)	0.0250 (0.0213, 0.0301)	0.0306 (0.0265, 0.0356)	0.0073 (0.0047, 0.0107)
	10 000	0.0170 (0.0148, 0.0203)	0.0199 (0.0162, 0.0253)	0.0228 (0.0201, 0.0270)	0.0060 (0.0038, 0.0089)
	100 000	0.0145 (0.0121, 0.0186)	0.0163 (0.0131, 0.0233)	0.0191 (0.0162, 0.0220)	0.0052 (0.0030, 0.0079)
Life history	50	0.0419 (0.0311, 0.0526)	0.0543 (0.0371, 0.0712)	0.0758 (0.0629, 0.0908)	0.0159 (0.0068, 0.0287)
	1000	0.0284 (0.0247, 0.0336)	0.0324 (0.0245, 0.0442)	0.0440 (0.0364, 0.0521)	0.0099 (0.0049, 0.0190)
	10 000	0.0242 (0.0204, 0.0298)	0.0255 (0.0185, 0.0360)	0.0325 (0.0279, 0.0398)	0.0083 (0.0039, 0.0165)
	100 000	0.0220 (0.0169, 0.0281)	0.0209 (0.0150, 0.0321)	0.0268 (0.0228, 0.0322)	0.0074 (0.0033, 0.0155)
Morphology	50	0.0171 (0.0143, 0.0208)	0.0218 (0.0174, 0.0352)	0.0103 (0.0067, 0.0158)	0.00017 (0.000096, 0.00035)
	1000	0.0103 (0.0086, 0.0123)	0.0207 (0.0167, 0.0262)	0.0071 (0.0044, 0.0098)	0.00015 (0.000081, 0.00030)
	10 000	0.0080 (0.0066, 0.0096)	0.0209 (0.0143, 0.0259)	0.0053 (0.0035, 0.0081)	0.00013 (0.000075, 0.00028)
	100 000	0.0066 (0.0053, 0.0081)	0.0199 (0.0120, 0.0257)	0.0049 (0.0029, 0.0073)	0.00013 (0.000073, 0.00027)
Plants	50	0.0503 (0.0395, 0.0629)	0.0764 (0.0613, 0.1011)	0.1694 (0.1273, 0.2319)	0.1867 (0.0627, 0.5280)
	1000	0.0298 (0.0229, 0.0364)	0.0468 (0.0370, 0.0591)	0.1038 (0.0776, 0.1397)	0.1480 (0.0637, 0.4441)
	10 000	0.0224 (0.0177, 0.0282)	0.0361 (0.0280, 0.0460)	0.0802 (0.0582, 0.1117)	0.1448 (0.0590, 0.4256)
	100 000	0.0180 (0.0143, 0.0231)	0.0289 (0.0229, 0.0392)	0.0667 (0.0472, 0.0976)	0.1447 (0.0548, 0.4162)
Vertebrates	50	0.0185 (0.0162, 0.0219)	0.0109 (0.0079, 0.0144)	0.0536 (0.0456, 0.0617)	0.0074 (0.0042, 0.0116)
	1000	0.0179 (0.0158, 0.0208)	0.0101 (0.0076, 0.0135)	0.0306 (0.0263, 0.0354)	0.0048 (0.0027, 0.0071)
	10 000	0.0177 (0.0155, 0.0206)	0.0100 (0.0075, 0.0134)	0.0235 (0.0200, 0.0269)	0.0036 (0.0021, 0.0057)
	100 000	0.0176 (0.0153, 0.0205)	0.0097 (0.0074, 0.0134)	0.0186 (0.0162, 0.0220)	0.0031 (0.0018, 0.0050)

Considering the form of selection, there was more variability in quadratic selection gradients at *N *=* *50 than *N *=* *10 000 or 100 000 for both taxa and for life‐history traits, whereas morphology quadratic gradients were similarly variable across population sizes. There was no evidence for differences in quadratic differential residual variance with *N* for any trait class or taxa.

## Discussion

Our meta‐analysis found little evidence for consistent, directional differences in *h* ^2^ or the extent of natural selection across a wide gradient of *N* in nature. These are notable results given our very large databases and the general lack of research investigating patterns of selection in relation to population size in wild species. Our results are not consistent with previous laboratory studies and the widespread view that small populations should have lower amounts of quantitative genetic variation than large populations in nature: no significant effects of *N* on *h* ^2^ were observed in either the meta‐analysis or unweighted analysis. Moreover, our results did not support that the magnitude, direction or form of selection systematically differs in small and large natural populations: it appears that natural populations of varying sizes experience a variety of environmental conditions, without consistently differing habitat quality at small population size. Indeed, CI of the magnitude of linear coefficients overlapped for all *N* bins, *N* had no effect on directional selection, and *N* had little effect on the form of quadratic selection acting on different trait classes and taxa. While the direction of linear selection decreased weakly with increasing *N* across different trait classes, the effect was only significant for life‐history traits and the effect size of the relationship was small.

Our selection results also lend some support to the Variable hypothesis that habitat fragmentation increases among‐fragment variability in habitat types and, by extension, variability in selective pressures. For three of four types of selection coefficients, residual variance decreased significantly with increasing *N* in most cases across the different trait classes and taxa, although this pattern was typically only apparent between the smallest and largest reference *N* (i.e. 50 vs ≥10 000 individuals). The lack of detectable heteroscedasticity for quadratic differentials may have been due to limited data available. While *h* ^2^ generally did not exhibit greater heteroscedasticity with decreasing *N*, observed patterns of *h* ^2^ across varying *N* may still be consistent with the Variable hypothesis: as long as within‐fragment selective pressures remain relatively stable, *h* ^2^ values may remain relatively consistent across habitat fragments and small populations despite potentially more variable selection pressures. Conversely, we found little evidence for the consistent differences in *h* ^2^ and selection between small and large populations predicted by the Directional Hypothesis and that might be expected if, for instance, environmental conditions were generally more stressful in small fragments or if genetic drift consistently eroded *h* ^2^ in small populations.

Collectively, while genetic drift and selection operate simultaneously in nature, our findings suggest, at least indirectly, that drift may not always overwhelm selection at small population size and that populations of varying size may respond to environmental change. The obvious question stemming from these observations is why we found no evidence for differences in *h* ^2^ and selection in relation to *N* in nature?

In regard to *h* ^2^, contemporary genetic structuring among natural populations is the product of a long evolutionary history. Thus, long‐term fluctuating spatial and temporal environmental conditions may have resulted in complex fluctuating selective pressures leading to a lack of relationship between *h* ^2^ and *N* in our meta‐analysis (e.g. Blanckenhorn et al. 1999; Siepielski et al. 2009, 2013). Another possibility is that for some traits, phenotypic plasticity or fitness trade‐offs might help to buffer loss of *V*_A_ at small *N* (Rollinson and Rowe 2015; Wood and Fraser 2015). For example, positive directional selection on juvenile size is common, but evolution is often not observed, possibly due to competing selection against investment per offspring in adults (Rollinson and Rowe 2015). Additionally, some small populations in our database may have only recently declined in *N* and thus *V*_A_ might still be bolstered by dominance or epistatic variance (Barton and Turelli 2004). Even under purely additive models, *V*_A_ is predicted to decrease by 1/(2*N*_e_) per generation and requires 1.4*N*_e_ generations to decline by 50% (Lynch and Hill 1986), so many decades might be required to produce detectable differences in *h* ^2^ among populations varying in *N*. Finally, we could not account for the degree of isolation of most study populations owing to a lack of information on gene flow. Even small amounts of gene flow can help to retain genetic variation in small populations that would otherwise be lost by persistent genetic drift and inbreeding depression; this might have affected the observations regarding patterns of *h* ^2^ in relation to *N* (Lynch 1991; Lenormand 2002; Jamieson and Allendorf 2012). When we excluded data on taxa where gene flow is prevalent (birds), we still found no effect of *N* on *h* ^2^ (Appendix S6, Table F2), but this subanalysis reduced sample size, and gene flow might also occur in populations of other taxa in our data set. The salient point is that research investigating *h* ^2^ in natural populations that are demonstrably isolated is sorely needed. Yet for some species, even isolated populations might not support the expected relationship between quantitative genetic variation and *N* (e.g. Wood et al. 2015).

In regard to selection and *N*, our results suggest that the extent of selection among natural populations is a product of random processes. For small populations, random habitat fragmentation appears to lead to an increase in among‐fragment variability of habitat types and therefore environmental conditions as predicted by the Variable hypothesis. Selection was frequently less variable at large *N* even though the Variable hypothesis predicts within‐habitat environmental variability to be similarly large across different large habitats. It is possible that studies with temporally replicated estimates of selection may specifically target populations inhabiting stable environments and experiencing stable selective pressures since this permits the repeat application of standardized methods of data collection and analysis (Morrissey and Hadfield 2012). Indeed, the selection database was heavily biased in favour of longitudinal studies. Admittedly, it is difficult to definitively test the Variable hypothesis for large populations with the meta‐analysis data; the best test of the Variable hypothesis in large *N* populations would require quantifying and comparing selection across the full gradient of conditions in multiple, large habitats acting on a single fitness‐related trait. Such data, to our knowledge, do not exist.

A related question to why we found no relationship between *N* and *h* ^2^ or *N* and selection is why laboratory studies consistently find evidence for reduced *h* ^2^ (and also reduced response to selection) in small *N* populations. Laboratory experiments tend to use similar, simplified starting conditions across populations even though this is unlikely to occur regularly in nature. Among small, natural populations, conditions immediately after fragmentation likely vary and this will ultimately influence the fate of each population in terms of *h* ^2^ and response to selection; it is possible that our meta‐analysis is capturing a signal of this variability. This raises important alternative questions: are studies that report genetic rescue effects and the extinction vortex in small, natural populations an example of sampling bias? Or is it that small, natural populations really do suffer from reduced quantitative genetic variation and response to selection, but our meta‐analysis was simply unable to track this with the currently available data? For example, no instances were found where *N* and selection coefficient estimates or *h* ^2^ were obtained on the same populations for amphibians, despite being one of the planet's most threatened taxonomic groups (IUCN 2013). Likewise, there were very few reptilian and invertebrate populations with *N* estimates, and even for mammals, data were restricted to a relatively small number of well‐studied systems. A truly representative database would also require data on populations that are both rare and widespread, habitat specialists and generalists; the literature is biased towards widespread, generalist, diploid species. Herein, we acknowledge that an inherent conundrum exists: studies on rare, habitat specialist species are sorely needed, but these are often the most challenging species to study and may require the development of alternative methods of data collection. Finally, it is possible that the meta‐analysis populations are not a random sample of all small populations that have existed for a given species but merely represent populations that have managed to persist over time; other populations may have already been driven to extinction by the cumulative effects predicted by the small population paradigm (i.e. extinction vortex, *sensu* Gilpin and Soulé 1986; Fagan and Holmes 2006).

#### Meta‐analysis limitations, caveats and considerations

Our meta‐analysis reveals at least eight primary limitations and uncertainties to consider in future research programmes on the evolutionary genetics of small, natural populations, in addition to the aforementioned need for more research on adaptive potential and selection in groups of isolated populations across a more taxonomically balanced spectrum.

First, as previously reported (e.g. Mousseau and Roff 1987), our meta‐analysis revealed significantly reduced *h* ^2^ for life‐history compared with morphology traits, but data are heavily biased towards estimates of *h* ^2^ for morphology (1136 of 1735 *h* ^2^ estimates); behaviour and physiological traits were under‐represented in the *h* ^2^ and selection databases. Yet, these traits are likely critical for adapting to environmental change. Whether all trait types are equally homogenous in relation to *N* is uncertain, although *V*_A_ and *h* ^2^ for behavioural traits were recently found to not differ between small and large isolated fish populations (Wood et al. 2015).

Second, most *h* ^2^ estimation methods produced similar values except that Bayesian *h* ^2^ was significantly lower than *h* ^2^ from parent–offspring regression; hence, adaptive potential approximated using Bayesian *h* ^2^ might be consistently biased downwards. Nevertheless, as Bayesian modelling is further adopted in quantitative genetic analyses, the problem of comparing *h* ^2^ derived from nonequivalent methods will likely become less of an issue. Of course, the accurate estimation of *h* ^2^ by any method depends on the quantity and quality of data available. If these are lacking or models are not properly specified, these will affect the estimation of *h* ^2^ and response to selection under the breeder's equation.

Third, responses to selection will likely depend on the nature of the selection pressures acting on particular traits (Willi et al. 2006), but information was not available for traits in the *h* ^2^ analysis to explore this. In a given population, there might be stronger directional selection pressures for certain traits and weaker selection for others, such that *h* ^2^ might be low for some traits but not others. Moreover, for traits not currently under selection in a small, isolated population, the expectation would be reduced *h* ^2^, consistent with predictions for neutral molecular variation (Lynch and Hill 1986). Thus, responses to selection based on the breeder's equation (*R* = *h* ^2^ *S*) become more complex even within a single population because selection and *h* ^2^ are not constant if there is ongoing directional selection altering levels of genetic variation, if trait interactions prevent response to selection, or if ongoing balancing selection maintains genetic variation.

Fourth, *N* and not *N*_e_ was used as the population size metric in our analyses as *N*_e_ was rarely estimated on the same populations as selection, but it is *N*_e_ that dictates rates of genetic drift and inbreeding. Yet our database *N* values ranged from four to one million, implying very small to large *N*_e_: 30 populations in the selection database (17% of the total) and 16 populations in the *h* ^2^ database (11% of the total) had *N *<* *50, well below the minimum population size at which populations are expected to disproportionately experience inbreeding and reduced adaptive potential (Willi et al. 2006; Frankham et al. 2014). Moreover, Mittell et al. (2015) found no relationship between *h* ^2^ and molecular diversity, another reasonable proxy for *N*_e_.

Fifth, multiyear *N* data were available for only a proportion of meta‐analysis populations, so we cannot confirm long‐term *N* for most populations. Fluctuating *N* could affect our conclusions by altering the relationship between *h* ^2^ and *N*. For example, if a population that underwent a severe bottleneck, or that colonized a new area from a relatively small number of individuals, subsequently increased in *N*, it might exhibit lower quantitative genetic variation for some traits than expected based on *N* alone.

Sixth, while selection is frequently measured for large populations, there are fewer attempts to formally quantify their *N*, likely because such populations might occupy a large range, or because of uncertainty in demarcating spatial boundaries. As the selection database was weighted towards populations of *N *<* *100–1000, more studies are required which measure selection and *N* for very large populations; in addition to the comparison of small, isolated versus very small, isolated populations (*N*_*e*_ <10), these will provide an important contrast for comparison with selection at small *N*.

Seventh, there might be a systematic bias in the types of populations/taxa chosen for selection or *h* ^2^ studies. For example, *h* ^2^ estimates might be biased towards species where genetic relatedness is easy to determine in the field, or species that lend themselves well to common garden experimentation. Likewise, selection studies might target populations inhabiting stable environments to facilitate the use of standardized methods and to estimate and compare selection over multiple years.

Finally, a greater adoption of multivariate approaches to studying evolution in natural populations is needed. Many traits are not independent but rather correlated. Direct selection resulting in an increase in the phenotypic value of one trait can result in indirect selection on a correlated trait to increase it, decrease it, or even constrain it; *h* ^2^ is not the only indicator of adaptive potential and is not necessarily the best, and the univariate breeder's equation may not adequately detect constraints to evolution in small populations. Few studies have examined multivariate metrics of adaptive potential in wild populations (e.g. Roff et al. 2004; Garant et al. 2008; Morrissey et al. 2012), and none have related such metrics to *N*.

#### Conservation implications

Our review is the first to investigate links between natural selection and *N*, and quantitative genetic variation with *N* across taxa. Similarities in patterns of both selection and *h* ^2^ across a wide range of *N* were observed suggesting that populations of various *N* and *N*_*e*_ experience a variety of environmental conditions; our findings support previous assertions that *h* ^2^ might only be reduced at extremely small *N* (*N*_*e*_ < 10: Willi et al. 2006; Wood et al. 2015). If these results are not exceptional, response to selection at small *N* might be more extensive than previously assumed in evolutionary and conservation biology. Collectively, species conservation initiatives and priority setting should consider that (i) the evolutionary trajectories of some small populations appear to be very much affected by natural selection; (ii) different small and large populations of the same species may contain variation that is adaptive in a wide range of circumstances; and (iii) minimum viable population sizes for some species – genetically, strictly speaking – may not need to be as high as previously discussed (see Frankham et al. 2013).

Although we have focused on the potential influence of *h* ^2^ for adaptive evolution in relation to *N*,* h* ^2^ is not the only metric of adaptive potential. Other examples include additive genetic variation (*V*_A_), the coefficient of additive genetic variation (CV_A_), the analysis of quantitative trait loci (QTLs) and standing levels of genomewide genetic diversity (e.g. Falconer and Mackay 1996; Harrisson et al. 2014). Hansen et al. (2011) championed a mean‐scaled evolvability metric (*I*_A_) over variance‐standardized *h* ^2^ suggesting that the latter might be more closely linked to adaptive potential. The strength of local adaptation and/or habitat quality (Yates and Fraser 2014) and the extent of inbreeding depression (Willi et al. 2006) will almost certainly influence population responses to environmental change, as may the extent of phenotypic plasticity, at least in the short term (Wood and Fraser 2015). Nor is quantitative genetic variation the only means by which populations, including small populations, can cope with environmental change. For example, many asexual species have persisted in the face of environmental variation through time by plasticity via epigenetic modification, without a specific mechanism for generating genetic variation (Rapp and Wendel 2005; Beldade et al. 2011). We might be tempted to ask then, what is the minimum amount of genetic diversity required for populations to persist for a given length of time? As suggested by Reed (2010) and as supported by our results, this question may only be addressed on a case‐by‐case basis and will intimately depend on factors such as environmental conditions, the rate and magnitude of environmental change, and the genetic characteristics of the population or species of interest.

The challenge in finding studies of completely isolated populations for their relevance to assessing the full effects of habitat fragmentation also raises important questions: is it simply that population isolation is not a prerequisite in many research studies (and hence such populations are rarely the focus of scientific inquiry)? Or is gene flow more pervasive than typically assumed? If the latter is true, this might substantially improve the potential of small populations to adapt; even very small amounts of gene flow might be sufficient to allow the rapid spread of advantageous alleles across populations (Slatkin 1976; Morjan and Rieseberg 2004).

Such questions are of critical interest to conservation biology but are beyond the scope of this study. What our results imply is that small populations – particularly those of generalist species – may not always occupy suboptimal habitats resulting in more rapid loss of quantitative genetic variation and adaptive potential (Frankham 1996; Willi et al. 2006; Kawecki 2008). In such a situation, even though genetic drift might indeed become more important as *N* decreases, selection may also be stronger in some habitat fragments if conditions become more extreme or variable as fragment size decreases. If this is true, some small populations in nature may retain their ability to adapt to future environmental change. On the other hand, if strong selection leads to excessive mortality, this will have a disproportionately larger demographic cost for small than large populations with two potential outcomes for small populations: (i) strong selection might impede response to selection by feeding back into the breeder's equation via reduced *h* ^2^ if *N*_e_ is reduced in addition to *N*, or (ii) for very small populations, the increased mortality may lead to population extinction by environmental or demographic stochasticity before adaptability becomes a problem.

## Data archiving

Data available from the Dryad Digital Repository: http://dx.doi:10.5061/dryad.rd5rn.

## Supporting information


**Appendix S1.** The relationship between sample size and *N* for four types of selection coefficients.

**Appendix S2.** The relationship between standard error and *N* for four types of selection coefficients.

**Appendix S3.** Reference list of studies included in the *h* ^2^ database.

**Appendix S4.** Summary of *h* ^2^ and selection database characteristics.

**Appendix S5.** Reference list of studies included in the selection database.

**Appendix S6.** Results of models to investigate the effect of *N* on *h* ^2^ data using MCMCglmm.

**Appendix S7.** Posterior modes of unweighted *h* ^2^ values including all vertebrate data and weighted and unweighted *h* ^2^ values excluding bird data estimated using four different methods of analysis within each of three different trait classes.

**Appendix S8.** Results of models to investigate the effect of *N* on selection coefficient data using MCMCglmm.

**Appendix S9.** Posterior modes of weighted and unweighted values for all vertebrate data and excluding bird data for the magnitude of two types of selection coefficients in relation to *N* bins.
