# Supplementary material for: Are heritability and selection related to population size in nature? Meta‐analysis and conservation implications
Source: Evol Appl. 2016 Apr 3;9(5):640–57. doi: 10.1111/eva.12375 (PMC4869407; doi:10.1111/eva.12375)

Appendix A: The relationship between standard error and log harmonic mean *N* for a) linear selection gradients, b) linear selection differentials, c) quadratic selection gradients, and d) quadratic selection differentials.


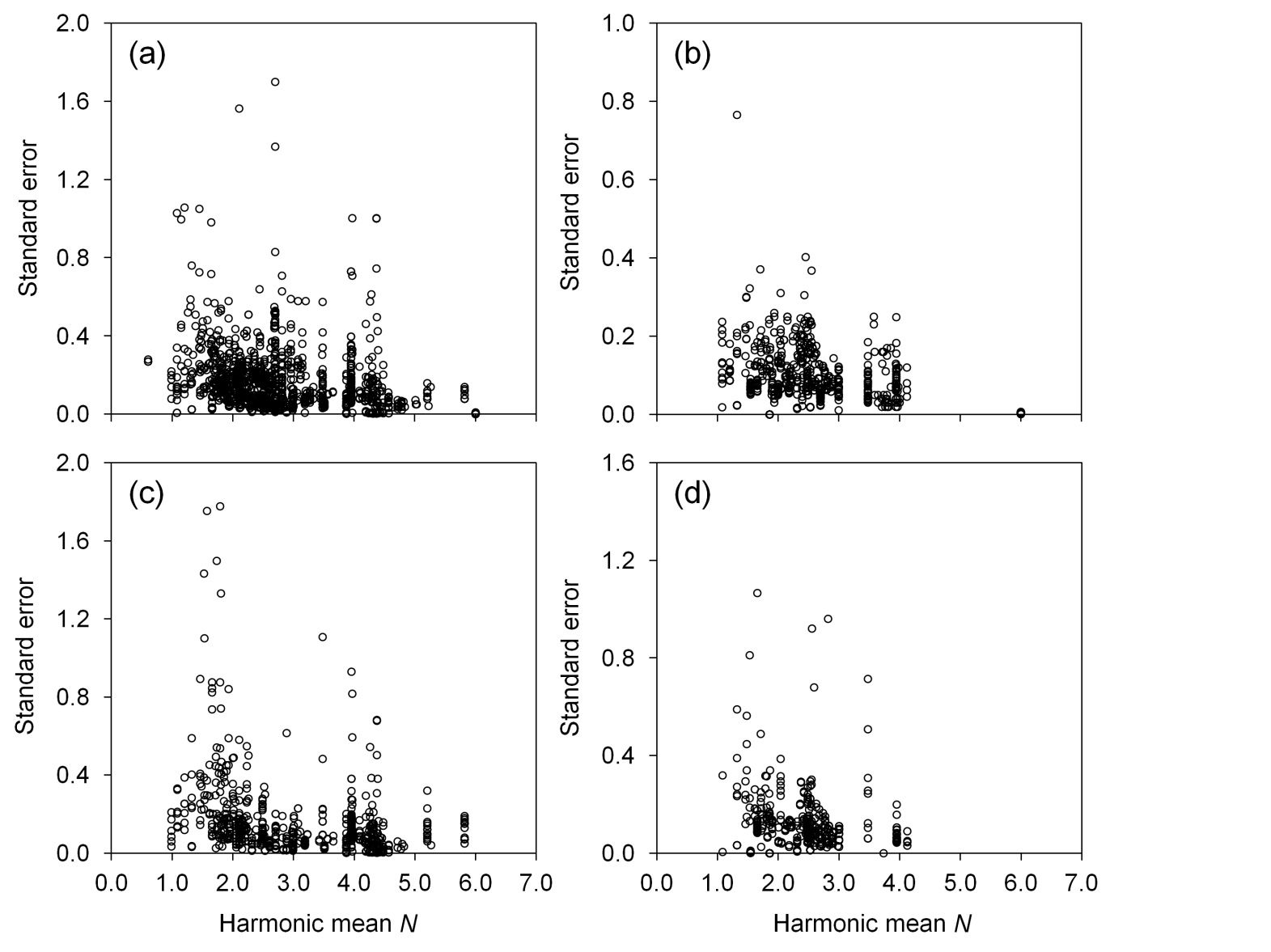

Supplement: Supplementary file 1 — Appendix S1. The relationship between sample size and N for four types of selection coefficients. [file EVA-9-640-s001.docx]
