# Supplementary material for: Are heritability and selection related to population size in nature? Meta‐analysis and conservation implications
Source: Evol Appl. 2016 Apr 3;9(5):640–57. doi: 10.1111/eva.12375 (PMC4869407; doi:10.1111/eva.12375)
Supplement: Supplementary file 3 — Appendix S3. Reference list of studies included in the h 2 database. [file EVA-9-640-s003.docx]

Appendix C: List of studies included in the *h^2^* database.

Agrawal, A. A., J. K. Conner, M. T. J. Johnson, and R. Wallsgrove. 2002. Ecological genetics of an induced plant defense against herbivores: additive genetic variance and costs of phenotypic plasticity. Evolution 56:2206–2213.

Åkesson, M., S. Bensch, and D. Hasselquist. 2007. Genetic and phenotypic associations in morphological traits: a long term study of great reed warblers Acrocephalus arundinaceus. Journal of Avian Biology 38:58–72.

Åkesson, M., S. Bensch, D. Hasselquist, M. Tarka, and B. Hansson. 2008. Estimating heritabilities and genetic correlations: comparing the “animal model” with parent-offspring regression using data from a natural population. PLoS ONE 3:e1739.

Alatalo, R. V., L. Gustafsson, and A. Lundberg. 1984. High frequency of cuckoldry in pied and collared flycatchers. Oikos 42:41–47.

Alatalo, R. V., L. Gustafsson, and A. Lundberg. 1989. Extra-pair paternity and heritability estimates of tarsus length in pied and collared flycatchers. Oikos 56:54–58.

Alatalo, R. V., L. Gustafsson, and A. Lundberg. 1990. Phenotypic selection on heritable size traits: environmental variance and genetic response. The American Naturalist 135:464–471.

Alcántara, J. M., R. Jaime, J. M. Bastida, and P. J. Rey. 2014. The role of genetic constraints on the diversification of Iberian taxa of the genus Aquilegia (Ranunculaceae). Biological Journal of the Linnean Society 111:252–261.

Badyaev, A., and T. Martin. 2000. Sexual dimorphism in relation to current selection in the house finch. Evolution 54:987–997.

Barbraud, C. 2000. Natural selection on body size traits in a long-lived bird, the snow petrel Pagodroma nivea. Journal of Evolutionary Biology 13:81–88.

Beacham, T. D. 1990. A genetic analysis of meristic and morphometric variation in chum salmon (Oncorhynchus keta) at three different temperatures. Canadian Journal of Zoology 68:225–229.

Beraldi, D., A. F. McRae, J. Gratten, J. G. Pilkington, J. Slate, P. M. Visscher, and J. M. Pemberton. 2007. Quantitative trait loci (QTL) mapping of resistance to strongyles and coccidia in the free-living Soay sheep (Ovis aries). International Journal for Parasitology 37:121–129.

Bérénos, C., P. A. Ellis, J. G. Pilkington, and J. M. Pemberton. 2014. Estimating quantitative genetic parameters in wild populations: a comparison of pedigree and genomic approaches. Molecular Ecology 23:3434–3451.

Biino, G., M. A. Palmas, C. Corona, D. Prodi, M. Fanciulli, R. Sulis, A. Serra, M. Fossarello, and M. Pirastu. 2005. Ocular refraction: heritability and genome-wide search for eye morphometry traits in an isolated Sardinian population. Human Genetics 116:152–159.

Blanc, J. M., J. D. McIntyre, and R. C. Simon. 2003. Genetic variation of resistance to mercury poisoning in steelhead (Oncorhynchus mykiss) alevins. Heredity 91:255–261.

Blumstein, D. T., A. J. Lea, L. E. Olson, and J. G. A. Martin. 2010. Heritability of anti-predatory traits: vigilance and locomotor performance in marmots. Journal of Evolutionary Biology 23:879–887.

Boag, P. T. 1983. The heritability of external morphology in Darwin’s grounds finches (Gespiza) on Ilsa Daphne Major, Galapagos. Evolution 37:877–894.

Boag, P. T., and P. R. Grant. 1978. Heritability of external morphology in Darwin’s finches.

Bortolotti, G. R., J. L. Tella, M. G. Forero, R. D. Dawson, and J. J. Negro. 2000. Genetics, local environment and health as factors influencing plasma carotenoids in wild American kestrels (Falco sparverius). Proceedings of the Royal Society B: Biological Sciences 267:1433–1438.

Boulinier, T., G. Sorci, J. Y. Monnat, and E. Danchin. 1997. Parent-offspring regression suggests heritable susceptibility to ectoparasites in a natural population of Kittiwake Rissa tridactyla. Journal of Evolutionary Biology 10:77–85.

Brommer, J. E., K. Ahola, and T. Karstinen. 2005a. The colour of fitness: plumage coloration and lifetime reproductive success in the tawny owl. Proceedings of the Royal Society B: Biological Sciences 272:935–940.

Brommer, J. E., M. Kirkpatrick, A. Qvamström, and L. Gustafsson. 2007a. The intersexual genetic correlation for lifetime fitness in the wild and its implications for sexual selection. PLoS ONE 2:1–6.

Brommer, J. E., and E. Kluen. 2012. Exploring the genetics of nestling personality traits in a wild passerine bird: Testing the phenotypic gambit. Ecology and Evolution 2:3032–3044.

Brommer, J. E., J. Merila, B. C. Shedldon, and L. Gustafsson. 2005b. Natural selection and genetic variation for reproductive reaction norms in a wild bird population. Evolution 59:1362–1371.

Brommer, J. E., and K. Rattiste. 2008. “Hidden” reproductive conflict between mates in a wild bird population. Evolution 62:2326–2333.

Brommer, J. E., K. Rattiste, and A. Wilson. 2010. The rate of ageing in a long-lived bird is not heritable. Heredity 104:363–370.

Brommer, J. E., K. Rattiste, and A. J. Wilson. 2008. Exploring plasticity in the wild: laying date-temperature reaction norms in the common gull Larus canus. Proceedings of the Royal Society B: Biological Sciences 275:687–693.

Brommer, J. E., A. J. Wilson, and L. Gustafsson. 2007b. Exploring the genetics of aging in a wild passerine bird. The American naturalist 170:643–650.

Brown, G. P., and R. Shine. 2007. Repeatability and heritability of reproductive traits in free-ranging snakes. Journal of Evolutionary Biology 20:588–596.

Bushuev, A. V., A. Husby, H. Sternberg, and V. G. Grinkov. 2012. Quantitative genetics of basal metabolic rate and body mass in free-living pied flycatchers. Journal of Zoology 288:245–251.

Campbell, D. R., S. G. Weller, A. K. Sakai, T. M. Culley, P. N. Dang, and A. K. Dunbar-Wallis. 2011. Genetic variation and covariation in floral allocation of two species of schiedea with contrasting levels of sexual dimorphism. Evolution 65:757–770.

Caro, S. P., A. Charmantier, M. M. Lambrechts, J. Blondel, J. Balthazart, and T. D. Williams. 2009. Local adaptation of timing of reproduction: females are in the driver’s seat. Functional Ecology 23:172–179.

Carr, D. E., and C. B. Fenster. 1994. Levels of genetic variation and covariation for Mimulus (Scrophulariaceae) floral traits. Heredity 72:606–618.

Caruso, C. M. 2004. The quantitative genetics of floral trait variation in Lobelia: potential constraints on adaptive evolution. Evolution 58:732–740.

Caruso, C. M., H. Maherali, A. Mikulyuk, K. Carlson, and R. B. Jackson. 2005. Genetic variance and covariance for physiological traits in Lobelia: are there constraints on adaptive evolution? Evolution 59:826–837.

Castellanos, M. C., J. M. Alcántara, P. J. Rey, and J. M. Bastida. 2011. Intra-population comparison of vegetative and floral trait heritabilities estimated from molecular markers in wild Aquilegia populations. Molecular Ecology 20:3513–3524.

Ceplitis, A., and B. O. Bengtsson. 2004. Genetic variation, disequilibrium and natural selection on reproductive traits in Allium vineale. Journal of Evolutionary Biology 17:302–311.

Charmantier, A., M. Buoro, O. Gimenez, and H. Weimerskirch. 2011. Heritability of short-scale natal dispersal in a large-scale foraging bird, the wandering albatross. Journal of Evolutionary Biology 24:1487–1496.

Charmantier, A., A. J. Keyser, and D. E. L. Promislow. 2007. First evidence for heritable variation in cooperative breeding behaviour. Proceedings of the Royal Society B: Biological Sciences 274:1757–1761.

Charmantier, A., L. E. B. Kruuk, J. Blondel, and M. M. Lambrechts. 2004a. Testing for microevolution in body size in three blue tit populations. Journal of Evolutionary Biology 17:732–743.

Charmantier, A., L. E. B. Kruuk, and M. M. Lambrechts. 2004b. Parasitism reduces the potential for evolution in a wild bird population. Evolution 58:203–206.

Charmantier, A., C. Perrins, R. H. McCleery, and B. C. Sheldon. 2006a. Evolutionary response to selection on clutch size in a long-term study of the mute swan. The American Naturalist 167:453–465.

Charmantier, A., C. Perrins, R. H. McCleery, and B. C. Sheldon. 2006b. Age-dependent genetic variance in a life-history trait in the mute swan. Proceedings of the Royal Society B: Biological Sciences 273:225–232.

Charmantier, A., and D. Réale. 2005. How do misassigned paternities affect the estimation of heritability in the wild? Molecular Ecology 14:2839–2850.

Cheverud, J. M., and W. P. J. Dittus. 1992. Primate population studies at Polonnaruwa. II. Heritability of body measurements in a natural population of toque macaques (Macaca sinica). American Journal of Primatology 27:145–154.

Class, B., E. Kluen, and J. E. Brommer. 2014. Evolutionary quantitative genetics of behavioral responses to handling in a wild passerine. Ecology and Evolution 4:427–440.

Clements, M. N., T. H. Clutton-Brock, F. E. Guinness, J. M. Pemberton, and L. E. B. Kruuk. 2011. Variances and covariances of phenological traits in a wild mammal population. Evolution 65:788–801.

Coltman, D. W., P. O’Donoghue, J. T. Hogg, and M. Festa-Bianchet. 2005. Selection and genetic (co) variance in bighorn sheep. Evolution 59:1372–1382.

Coltman, D. W., P. O’Donoghue, J. T. Jorgenson, J. T. Hogg, C. Strobeck, and M. Festa-Bianchet. 2003. Undesirable evolutionary consequences of trophy hunting. Nature 426:655–658.

Coltman, D. W., J. Pilkington, L. E. Kruuk, K. Wilson, and J. M. Pemberton. 2001. Positive genetic correlation between parasite resistance and body size in a free-living ungulate population. Evolution 55:2116–2125.

Conner, J. K., and S. Via. 1993. Patterns of phenotypic and genetic correlations among morphological and life-history traits in wild radish, Raphanus raphanistrum. Evolution 47:704–711.

Cuenco, K. T., E. A. Ottesen, S. A. Williams, T. B. Nutman, and C. Steel. 2009. Heritable factors play a major role in determining host responses to Wuchereria bancrofti infection in an isolated South Pacific island population. The Journal of Infectious Diseases 200:1271–1278.

Davies, J. C., R. F. Rockwell, and F. Cooke. 1988. Body-size variation and fitness components in lesser snow geese, Chen caerulescens caerulescnens. The Auk 105:639–648.

DiBattista, J. D., K. A. Feldheim, D. Garant, S. H. Gruber, and A. P. Hendry. 2009. Evolutionary potential of a large marine vertebrate: quantitative genetic parameters in a wild population. Evolution 63:1051–1067.

DiBattista, J. D., K. A. Feldheim, D. Garant, S. H. Gruber, and A. P. Hendry. 2011. Anthropogenic disturbance and evolutionary parameters: a lemon shark population experiencing habitat loss. Evolutionary Applications 4:1–17.

Dickerson, B. R., M. F. Willson, P. Bentzen, and T. P. Quinn. 2005. Heritability of life history and morphological traits in a wild pink salmon population assessed by DNA parentage analysis. Transactions of the American Fisheries Society 134:1323–1328.

Doligez, B., L. Gustafsson, and T. Pärt. 2009. “Heritability” of dispersal propensity in a patchy population. Proceedings of the Royal Society B: Biological Sciences 276:2829–2836.

Eads, A. R., N. J. Mitchell, and J. P. Evans. 2012. Patterns of genetic variation in desiccation tolerance in embyros of the terrestrial-breeding frog, Pseudophryne guentheri. Evolution 66:2865–2877.

Etterson, J. R. 2004. Evolutionary potential of Chamaecrista fasciculata in relation to climate change. II. Genetic architecture of three populations reciprocally planted along an environmental gradient in the great plains. Evolution 58:1459–1471.

Findlay, C. S., and F. Cooke. 1982. Breeding synchrony in the lesser snow goose (Anser caerulescens caerulescens). I . Genetic and environmental components of hatch date variability and their effects on hatch synchrony. Evolution 36:342–351.

Findlay, C. S., and F. Cooke. 1983. Genetic and environmental components of clutch size variance in a wild population of lesser snow geese (Anser caerulescens caerulescens). Evolution 37:724–734.

Fletcher, Q. E., J. R. Speakman, S. Boutin, J. E. Lane, A. G. McAdam, J. C. Gorrell, D. W. Coltman, and M. M. Humphries. 2014. Daily energy expenditure during lactation is strongly selected in a free-living mammal. Functional Ecology:DOI: 10.1111/1365–2435.12313.

Flux, J. E. C., and M. M. Flux. 1982. Artificial selection and gene flow in wild starlings, Sturnus vulgaris. Naturwissenschaften 69:96–97.

Foerster, K., T. Coulson, B. C. Sheldon, J. M. Pemberton, T. H. Clutton-Brock, and L. E. B. Kruuk. 2007. Sexually antagonistic genetic variation for fitness in red deer. Nature 447:1107–1110.

Fornoni, J., P. L. Valverde, and J. Núñez-Farfán. 2003. Quantitative genetics of plant tolerance and resistance against natural enemies of two natural populations of Datura stramonium. Evolutionary Ecology Research 5:1049–1065.

Freeman-Gallant, C. R., and M. D. Rothstein. 1999. Apparent heritability of parental care in savannah sparrows. The Auk 116:1132–1136.

Frentiu, F. D., S. M. Clegg, M. W. Blows, and I. P. F. Owens. 2007. Large body size in an island-dwelling bird: a microevolutionary analysis. Journal of Evolutionary Biology 20:639–649.

Frère, C. H., M. Krützen, J. Mann, R. C. Connor, L. Bejder, and W. B. Sherwin. 2010. Social and genetic interactions drive fitness variation in a free-living dolphin population. Proceedings of the National Academy of Sciences of the United States of America 107:19949–19954.

Funk, W. C., J. A. Tyburczy, K. L. Knudsen, K. R. Lindner, and F. W. Allendorf. 2005. Genetic basis of variation in morphological and life-history traits of a wild population of pink salmon. Journal of Heredity 96:24–31.

Gallant, B. Y., D. Réale, and M. Festa-Bianchet. 2001. Does mass change of primiparous bighorn ewes reflect reproductive effort? Canadian Journal of Zoology 79:312–318.

Garant, D., J. J. Dodson, and L. Bernatchez. 2003. Differential reproductive success and heritability of alternative reproductive tactics in wild Atlantic salmon (Salmo salar L.). Evolution 57:1133–1141.

Garant, D., J. D. Hadfield, L. E. B. Kruuk, and B. C. Sheldon. 2008. Stability of genetic variance and covariance for reproductive characters in the face of climate change in a wild bird population. Molecular Ecology 17:179–188.

Garant, D., L. E. B. Kruuk, T. a Wilkin, R. H. McCleery, and B. C. Sheldon. 2005. Evolution driven by differential dispersal within a wild bird population. Nature 433:60–65.

Garant, D., L. Kruuk, R. McCleery, and B. Sheldon. 2004a. Evolution in a changing environment: a case study with great tit fledging mass. American Naturalist 164:E115–E129.

Garant, D., B. C. Sheldon, and L. Gustafsson. 2004b. Climatic and temporal effects on the expression of secondary sexual characters: genetic and environmental components. Evolution 58:634–644.

Garnett, M. C. 1981. Body Size, its heritability and influence on juvenile survival among great tits, Parus major. Ibis 123:31 – 41.

Gibbs, H. 1988. Heritability and selection on clutch size in Darwin’s medium ground finches (Geospiza fortis). Evolution 42:750–762.

Gienapp, P., E. Postma, and M. Visser. 2006. Why breeding time has not responded to selection for earlier breeding in a songbird population. Evolution 60:2381–2388.

Gögele, M., C. Pattaro, C. Fuchsberger, C. Minelli, P. P. Pramstaller, and M. Wjst. 2011. Heritability analysis of life span in a semi-isolated population followed across four centuries reveals the presence of pleiotropy between life span and reproduction. Journals of Gerontology - Series A Biological Sciences and Medical Sciences 66 A:26–37.

Goodger, J. Q. D., P. K. Ades, and I. E. Woodrow. 2004. Cyanogenesis in Eucalyptus polyanthemos seedlings: heritability, ontogeny and effect of soil nitrogen. Tree Physiology 24:681–688.

Gosler, A. G., P. R. Barnett, and S. J. Reynolds. 2000. Inheritance and variation in eggshell patterning in the great tit Parus major. Proceedings of the Royal Society B: Biological Sciences 267:2469–2473.

Gosler, A. G., and D. G. C. Harper. 2000. Assessing the heritability of body condition in birds: a challenge exemplified by the great tit Parus major L.(Aves). Biological Journal of the Linnean Society 71:103–117.

Graham, A. L., A. D. Hayward, K. A. Watt, J. G. Pilkington, J. M. Pemberton, and D. H. Nussey. 2010. Fitness correlates of heritable variation in antibody responsiveness in a wild mammal. Science 330:662–666.

Grant, B. R. 1990. The significance of subadult plummage in Darwin’s finches. Behavioural Ecology 1:161–170.

Grant, B. R., and P. R. Grant. 1996. Cultural inheritance of song and its role in the evolution of Darwin’s finches. Evolution 50:2471–2487.

Grant, P. R. 1983. Inheritance of size and shape in a population of Darwin’s finches, Geospiza conirostris. Proceedings of the Royal Society B: Biological Sciences 220:219–236.

Grant, P. R., and B. R. Grant. 1995. Predicting microevolutionary responses to directional selection on heritable variation. Evolution 49:241–251.

Greenwood, P., P. Greenwood, P. Harvey, P. Harvey, C. Perrins, and C. Perrins. 1979. The role of dispersal in the great tit (Parus major): the causes, consequences and heritability. The Journal of Animal Ecology 48:123–142.

Gustafsson, L., and J. Merilä. 1994. Foster parent experiment reveals no genotype — environment correlation in the external morphology of Ficedula albicoiis, the collared flycatcher. Heredity 73:124–129.

Hansson, B., S. Bensch, and D. Hasselquist. 2003. Heritability of dispersal in the great reed warbler. Ecology Letters 6:290–294.

Hasselquist, D., S. Bensch, and T. Vonschantz. 1995. Estimating cuckoldry in birds: the heritability method and DNA fingerprinting give different results. Oikos 72:173–178.

Hebert, K. P., P. L. Goddard, W. W. Smoker, and A. J. Gharrett. 1998. Quantitative genetic variation and genotype by environment interaction of embryo development rate in pink salmon (Oncorhynchus gorbuscha). Canadian Journal of Fisheries and Aquatic Sciences 55:2048–2057.

Hegyi, G., M. Herényi, A. J. Wilson, L. Z. Garamszegi, B. Rosivall, M. Eens, and J. Török. 2010. Breeding experience and the heritability of female mate choice in collared flycatchers. PLoS ONE 5:e13855.

Hochachka, W. 1993. Repeatable reproduction in song sparrows. The Auk 110:603–613.

Howard, R. D., H. H. Wmhteman, and T. I. Schueller. 2013. Sexual selection in American toads: a test of a good-genes hypothesis 48:1286–1300.

Hu, G., C. Wang, and Y. Da. 2014. Genomic heritability estimation for the early life-history transition related to propensity to migrate in wild rainbow and steelhead trout populations. Ecology and Evolution 4:1381–1388.

Husby, A., D. H. Nussey, M. E. Visser, A. J. Wilson, B. C. Sheldon, and L. E. B. Kruuk. 2010. Contrasting patterns of phenotypic plasticity in reproductive traits in two great tit (Parus major) populations. Evolution 64:2221–2237.

Husby, A., M. E. Visser, and L. E. B. Kruuk. 2011. Speeding up microevolution: the effects of increasing temperature on selection and genetic variance in a wild bird population. PLoS Biology 9:e1000585.

Imbert, E. 2001. Capitulum characters in a seed heteromorphic plant, Crepis sancta (Asteraceae): variance partitioning and inference for the evolution of dispersal rate. Heredity 86:78–86.

Van Der Jeugd, H. P., and R. McCleery. 2002. Effects of spatial autocorrelation, natal philopatry and phenotypic plasticity on the heritability of laying date. Journal of Evolutionary Biology 15:380–387.

Johnston, S. E., D. Beraldi, A. F. McRae, J. M. Pemberton, and J. Slate. 2010. Horn type and horn length genes map to the same chromosomal region in Soay sheep. Heredity 104:196–205.

Kaczorowski, R. L., T. E. Juenger, and T. P. Holtsford. 2008. Heritability and correlation structure of nectar and floral morphology traits in Nicotiana alata. Evolution 62:1738–1750.

Karell, P., K. Ahola, T. Karstinen, J. Valkama, and J. E. Brommer. 2011. Climate change drives microevolution in a wild bird. Nature communications 2:208.

Keller, L. F., P. R. Grant, B. R. Grant, and K. Petren. 2001. Heritability of morphological traits in Darwin’s Finches: misidentified paternity and maternal effects. Genetics 87:325–336.

Kelly, M. J. 2001. Lineage loss in Serengeti cheetahs: consequences of high reproductive variance and heritability of fitness on effective population size. Conservation Biology 15:137–147.

Kim, S. Y., H. Drummond, R. Torres, and A. Velando. 2011a. Evolvability of an avian life history trait declines with father’s age. Journal of Evolutionary Biology 24:295–302.

Kim, S. Y., J. C. Noguera, J. Morales, and A. Velando. 2010. Heritability of resistance to oxidative stress in early life. Journal of Evolutionary Biology 23:769–775.

Kim, S.-Y., J. A. Fargallo, P. Vergara, and J. Martínez-Padilla. 2013. Multivariate heredity of melanin-based coloration, body mass and immunity. Heredity 111:139–146.

Kim, S.-Y., J. C. Noguera, J. Morales, and A. Velando. 2011b. Quantitative genetic evidence for trade-off between growth and resistance to oxidative stress in a wild bird. Evolutionary Ecology 25:461–472.

Kinnison, M. T., M. J. Unwin, A. P. Hendry, and T. P. Quinn. 2001. Migratory costs and the evolution of egg size and number in introduced and indigenous salmon populations. Evolution 55:1656–1667.

Kinnison, M. T., M. J. Unwin, and T. P. Quinn. 2003. Migratory costs and contemporary evolution of reproductive allocation in male chinook salmon. Journal of Evolutionary Biology 16:1257–1269.

Kontiainen, P., J. E. Brommer, P. Karell, and H. Pietiäinen. 2008. Heritability, plasticity and canalization of Ural owl egg size in a cyclic environment. Journal of Evolutionary Biology 21:88–96.

Kruuk, L. E. B., J. Slate, J. M. Pemberton, and T. H. Clutton-Brock. 2003. Fluctuating asymmetry in a secondary sexual trait: no associations with individual fitness, environmental stress or inbreeding, and no heritability. Journal of Evolutionary Biology 16:101–113.

Kruuk, L. E., T. H. Clutton-Brock, J. Slate, J. M. Pemberton, S. Brotherstone, and F. E. Guinness. 2000. Heritability of fitness in a wild mammal population. Proceedings of the National Academy of Sciences of the United States of America 97:698–703.

Kruuk, L., J. Merilä, and B. Sheldon. 2001. Phenotypic selection on a heritable size trait revisited. The American Naturalist 158:557–571.

Kruuk, L., J. Slate, and J. Pemberton. 2002. Antler size in red deer: heritability and selection but no evolution. Evolution 56:1683–1695.

Kulbaba, M. W., and A. C. Worley. 2008. Floral design in Polemonium brandegei (Polemoniaceae): genetic and phenotypic variation under hawkmoth and hummingbird pollination. International Journal of Plant Sciences 169:509–522.

Kvalnes, T., T. H. Ringsby, H. Jensen, and B.-E. Sæther. 2012. Correlates of egg size variation in a population of house sparrow Passer domesticus. Oecologia:391–402.

De la Hera, I., T. E. Reed, F. Pulido, and M. E. Visser. 2013. Feather mass and winter moult extent are heritable but not associated with fitness-related traits in a long-distance migratory bird. Evolutionary Ecology 27:1199–1216.

Larsson, K. 1993. Inheritance of body size in the barnacle goose under different environmental conditions. Journal of Evolutionary Biology 6:195–208.

Larsson, K. 1996. Genetic and environmental effects on the timing of wing moult in the barnacle goose. Heredity 76:100–107.

Larsson, K., K. Larsson, P. Forslund, and P. Forslund. 1992. Genetic and social inheritance of body and egg size in the barnacle goose (Branta-Leucopsis). Evolution 46:244.

Larsson, K., K. Rattiste, and V. Lilleleht. 1997. Heritability of head size in the common gull Larus canus in relation to environmental conditions during offspring growth. Heredity 79:201–207.

Lawler, R. R. 2006. Sifaka positional behaviour: ontogenetic and quantitative genetic approaches. American Journal of Physical Anthropology 131:261–271.

Lea, A. J., D. T. Blumstein, T. W. Wey, and J. G. A. Martin. 2010. Heritable victimization and the benefits of agonistic relationships. Proceedings of the National Academy of Sciences of the United States of America 107:21587–21592.

Lessells, C. M., F. Cooke, and R. F. Rockwell. 1989. Is there a trade-off between egg weight and clutch size in wild lesser snow geese (Anser c. caerulescens)? Journal of Evolutionary Biology 472:457–472.

Lessells, C. M., and G. N. Ovenden. 1989. Heritability of wing length and weight in European bee-eaters (Merops apiaster). The Condor 91:210–214.

Liedvogel, M., C. K. Cornwallis, and B. C. Sheldon. 2012. Integrating candidate gene and quantitative genetic approaches to understand variation in timing of breeding in wild tit populations. Journal of Evolutionary Biology 25:813–823.

MacColl, A. D. C., and B. J. Hatchwell. 2003. Heritability of parental effort in a passerine bird. Evolution 57:2191–2195.

Marroni, F., D. Grazio, C. Pattaro, M. Devoto, and P. Pramstaller. 2008. Estimates of genetic and environmental contribution to 43 quantitative traits support sharing of a homogeneous environment in an isolated population from South Tyrol, Italy. Human heredity 65:175–182.

Martin, J. G. a, and M. Festa-Bianchet. 2012. Determinants and consequences of age of primiparity in bighorn ewes. Oikos 121:752–760.

McCleery, R. H., R. A. Pettifor, P. Armbruster, K. Meyer, B. C. Sheldon, and C. M. Perrins. 2004. Components of variance underlying fitness in a natural population of the great tit Parus major. The American naturalist 164:E62–E72.

Mcfarlane, S. E., J. C. Gorrell, D. W. Coltman, M. M. Humphries, S. Boutin, and A. G. Mcadam. 2014. Very low levels of direct additive genetic variance in fitness and fitness components in a red squirrel population. Ecology and Evolution 4:1729–1738.

McFarlane, S. E., J. E. Lane, R. W. Taylor, J. C. Gorrell, D. W. Coltman, M. M. Humphries, S. Boutin, and A. G. McAdam. 2011. The heritability of multiple male mating in a promiscuous mammal. Biology Letters 7:368–371.

McGaugh, S. E., L. E. Schwanz, R. M. Bowden, J. E. Gonzalez, and F. J. Janzen. 2010. Inheritance of nesting behaviour across natural environmental variation in a turtle with temperature-dependent sex determination. Proceedings of the Royal Society B: Biological Sciences 277:1219–1226.

Merila, J. 1996. Genetic variation in offspring condition: an experiment. Functional Ecology 10:465.

Merilä, J. 1997. Expression of genetic variation in body size of the collared flycatcher under different environmental conditions. Evolution 51:526–536.

Merila, J., and L. Gustafsson. 1993. Inheritance of size and shape in a natural population of collared flycatchers, Ficedula albicollis. Journal of Evolutionary Biology 6:375–395.

Merila, J., and L. Gustafsson. 1996. Temporal stability and microgeographic homogeneity of heritability estimates in a natural bird population. Journal of Heredity 87:199–204.

Merilä, J., L. E. B. Kruuk, and B. C. Sheldon. 2001. Natural selection on the genetical component of variance in body condition in a wild bird population. Journal of Evolutionary Biology 14:918–929.

Merilä, J., and B. C. Sheldon. 2000. Lifetime reproductive success and heritability in nature. The American Naturalist 155:301–310.

Merila, J., B. C. Sheldon, and H. Ellegren. 1998. Quantitative genetics of sexual size dimorphism in the collared flycatcher, Ficedula albicollis. Evolution 52:870–876.

Merilä, J., B. C. Sheldon, and S. C. Griffith. 2003. Heterotic effects on fitness in a wild bird population. Annales Zoologici Fennici 40:269–280.

Mihoub, J. B., N. G. Mouawad, P. Pilard, F. Jiguet, M. Low, and C. Teplitsky. 2012. Impact of temperature on the breeding performance and selection patterns in lesser kestrels Falco naumanni. Journal of Avian Biology 43:472–480.

Milner, J. M., J. M. Pemberton, S. Brotherstone, and S. D. Albon. 2000. Estimating variance components and heritabilities in the wild: a case study using the “animal model” approach. Journal of Evolutionary Biology 13:804–813.

Mitchell, R. J., and R. G. Shaw. 1993. Heritability of floral traits for the perennial wild flower Penstemon centranthifolius (Scrophulariaceae): clones and crosses. Heredity 71:185–192.

Mitchell-Olds, T. 1986. Quantitative genetics of survival and growth in Impatiens capensis. Evolution 40:107–116.

Mitchell-Olds, T., and J. Bergelson. 1990. Statistical genetics of an annual plant, Impatiens capensis. I. Genetic basis of quantitative variation. Genetics 124:407–415.

Morales, J., S. Y. Kim, E. Lobato, S. Merino, G. Tomás, J. Martínez-de la Puente, and J. Moreno. 2010. On the heritability of blue-green eggshell coloration. Journal of Evolutionary Biology 23:1783–1791.

Morrissey, M. B., and M. M. Ferguson. 2011. A test for the genetic basis of natural selection: an individual-based longitudinal study in a stream-dwelling fish. Evolution 65:1037–1047.

Nicolaus, M., J. E. Brommer, R. Ubels, J. M. Tinbergen, and N. J. Dingemanse. 2013. Exploring patterns of variation in clutch size-density reaction norms in a wild passerine bird. Journal of Evolutionary Biology 26:2031–2043.

Nilsson, J. Å., M. Åkesson, and J. F. Nilsson. 2009. Heritability of resting metabolic rate in a wild population of blue tits. Journal of Evolutionary Biology 22:1867–1874.

Van Noordwijk, A. J., J. H. Van Balen, and W. Scharloo. 1981a. Genetic and environmental variation in clutch size of the great tit (Parus major). Netherlands Journal of Zoology 31:342–372.

Van Noordwijk, A. J., J. H. Van Balen, and W. Scharloo. 1981b. Genetic variation in the timing of reproduction in the Great Tit. Oecologia 49:158–166.

Van Noordwijk, A. J., J. H. Van Balent, and W. Scharloo. 1988. Heritability of body size in a natural population of the great tit (Parus major) and its relation to age and environmental conditions during growth. Genetic Research 51:149–162.

Van Noordwijk, A. J., L. C. P. Keizer, J. H. Van Balen, and W. Scharloo. 1981c. Genetic variation in egg dimensions in natural populations of the Great Tit. Genetica 55:221–232.

Norris, K. 1993. Heritable variation in a plumage indicator of viability in male great tits Parus major. Nature 362:537–539.

Norris, K. J., and J. K. Blakey. 1989. Evidence for cuckoldry in the great tit Parus major. Ibis 131:436–442.

Nussey, D. H., E. Postma, P. Gienapp, and M. E. Visser. 2005. Selection on heritable phenotypic plasticity in a wild bird population. Science 310:304–306.

Olsen, J. B., Hard, J. J., Harper, K., Miller, S. J., and J. K. Wenburg. 2011. Heritability of Traits in wild Chinook salmon. Arctic Yukon Kuskokwim Sustainable Salmon Initiative report number XXXX.

O’Neil, P. 1997. Natural selection on genetically correlated phenological characters in Lythrum salicaria L.(Lythraceae). Evolution 51:267–274.

O’Neil, P., and J. Schmitt. 1993. Genetic constraints on the independent evolution of male and female reproductive characters in the tristylous plant Lythrum salicaria. Evolution 47:1457–1471.

Oh, K. P., and A. V. Badyaev. 2008. Evolution of adaptation and mate choice: parental relatedness affects expression of phenotypic variance in a natural population. Evolutionary Biology 35:111–124.

Páez, D. J., L. Bernatchez, and J. J. Dodson. 2011. Alternative life histories in the Atlantic salmon: genetic covariances within the sneaker sexual tactic in males. Proceedings of the Royal Society B: Biological Sciences 278:2150–2158.

Páez, D. J., M. Morrissey, L. Bernatchez, and J. J. Dodson. 2010. The genetic basis of early-life morphological traits and their relation to alternative male reproductive tactics in Atlantic salmon. Journal of Evolutionary Biology 23:757–768.

Paik, S. H., H.-J. Kim, H.-Y. Son, S. Lee, S.-W. Im, Y. S. Ju, J. H. Yeon, S. J. Jo, H. C. Eun, J.-S. Seo, O. S. Kwon, and J.-I. Kim. 2012. Gene mapping study for constitutive skin color in an isolated Mongolian population. Experimental and Molecular Medicine 44:241.

Papaïx, J., S. Cubaynes, M. Buoro, A. Charmantier, P. Perret, and O. Gimenez. 2010. Combining capture-recapture data and pedigree information to assess heritability of demographic parameters in the wild. Journal of Evolutionary Biology 23:2176–2184.

Pasinelli, G., K. Schiegg, and J. R. Walters. 2004. Genetic and environmental influences on natal dispersal distance in a resident bird species. American naturalist 164:660–669.

Pastorino, M. J., S. Ghirardi, J. Grosfeld, L. A. Gallo, and J. G. Puntieri. 2010. Genetic variation in architectural seedling traits of Patagonian cypress natural populations from the extremes of a precipitation range. Annals of Forest Science 67:508.

Pelletier, F., D. Réale, D. Garant, D. W. Coltman, and M. Festa-Bianchet. 2007. Selection on heritable seasonal phenotypic plasticity of body mass. Evolution 61:1969–1979.

Petit, C., H. Fréville, A. Mignot, B. Colas, M. Riba, E. Imbert, S. Hurtrez-Boussés, M. Virevaire, and I. Olivieri. 2001. Gene flow and local adaptation in two endemic plant species. Biological Conservation 100:21–34.

Pitala, N., L. Gustafsson, J. Sendecka, and J. E. Brommer. 2007. Nestling immune response to phytohaemagglutinin is not heritable in collared flycatchers. Biology letters 3:418–421.

Poissant, J., C. S. Davis, R. M. Malenfant, J. T. Hogg, and D. W. Coltman. 2012. QTL mapping for sexually dimorphic fitness-related traits in wild bighorn sheep. Heredity 108:256–263.

Poissant, J., D. Réale, J. G. A. Martin, M. Festa-Bianchet, and D. W. Coltman. 2013. A quantitative trait locus analysis of personality in wild bighorn sheep. Ecology and Evolution 3:474–481.

Poissant, J., A. J. Wilson, M. Festa-Bianchet, J. T. Hogg, and D. W. Coltman. 2008. Quantitative genetics and sex-specific selection on sexually dimorphic traits in bighorn sheep. Proceedings of the Royal Society B: Biological Sciences 275:623–628.

Postma, E., F. Heinrich, U. Koller, R. J. Sardell, J. M. Reid, P. Arcese, and L. F. Keller. 2011. Disentangling the effect of genes, the environment and chance on sex ratio variation in a wild bird population. Proceedings of the Royal Society B: Biological Sciences 278:2996–3002.

Postma, E., J. Visser, and A. J. Van Noordwijk. 2007. Strong artificial selection in the wild results in predicted small evolutionary change. Journal of Evolutionary Biology 20:1823–1832.

Potti, J. 1998. Arrival time from spring migration in male pied flycatchers: individual consistency and familial resemblance. Condor 100:702-708.

Potti, J. 1999. Maternal effects and the pervasive impact of nestling history on egg size in a passerine bird. Evolution 53:279–285.

Potti, J., and D. Canal. 2011. Heritability and genetic correlation between the sexes in a songbird sexual ornament. Heredity 106:945–954.

Potti, J., and S. Merino. 1994. Heritability estimates and maternal effects on tarsus length in pied flycatchers, Ficedula hypoleuca. Oecologia 100:331–338.

Potti, J., and S. Montalvo. 1991. Male color variation in Spanish pied flycatchers Ficedula- hypoleuca. Ibis 133:293–299.

Potti, J., J. Moreno, S. Merino, O. Frías, and R. Rodríguez. 1999. Environmental and genetic variation in the haematocrit of fledgling pied flycatchers Ficedula hypoleuca. Oecologia 120:1–8.

Price, T. D., and P. R. Grant. 1985. The evolution of ontogeny in Darwin’s finches: a quantitative genetic approach. American Naturalist 125:169–188.

Quinn, J. L., S. C. Patrick, S. Bouwhuis, T. A. Wilkin, and B. C. Sheldon. 2009. Heterogeneous selection on a heritable temperament trait in a variable environment. Journal of Animal Ecology 78:1203–1215.

Quinn, T. P., M. J. Unwin, and M. T. Kinnison. 2000. Evolution of temporal isolation in the wild: genetic divergence in timing of migration and breeding by introduced chinook salmon populations. Evolution 54:1372–1385.

Qvarnstrom, A. 1999. Genotype-by-environment interactions in the determination of the size of a secondary sexual character in the collared flycatcher (Ficedula albicollis). Evolution 53:1564–1572.

Qvarnström, A., J. E. Brommer, and L. Gustafsson. 2006. Testing the genetics underlying the co-evolution of mate choice and ornament in the wild. Nature 441:84–86.

Réale, D., D. Berteaux, A. McAdam, and S. Boutin. 2003. Lifetime selection on heritable life-history traits in a natural population of red squirrels. Evolution 57:2416–2423.

Réale, D., and M. Festa-Bianchet. 2000a. Mass-dependent reproductive strategies in wild bighorn ewes: a quantitative genetic approach. Journal of Evolutionary Biology 13:679–688.

Réale, D., and M. Festa-Bianchet. 2000b. Quantitative genetics of life-history traits in a long-lived wild mammal. Heredity 85:593–603.

Réale, D., M. Festa-Bianchet, and J. T. Jorgenson. 1999. Heritability of body mass varies with age and season in wild bighorn sheep. Heredity 83:526–532.

Reid, J. M., P. Arcese, R. J. Sardell, and L. F. Keller. 2011a. Heritability of female extra-pair paternity rate in song sparrows (Melospiza melodia). Proceedings of the Royal Society B: Biological Sciences 278:1114–1120.

Reid, J. M., P. Arcese, R. J. Sardell, and L. F. Keller. 2011b. Additive genetic variance, heritability, and inbreeding depression in male extra-pair reproductive success. American Naturalist 177:177–187.

Reid, J. M., and R. J. Sardell. 2012. Indirect selection on female extra-pair reproduction? Comparing the additive genetic value of maternal half-sib extra-pair and within-pair offspring. Proceedings of the Royal Society B: Biological Sciences 279:1700–1708.

Riddington, R., and A. Gosler. 1995. Differences in reproductive success and parental qualities between habitats in the great tit Parus major. Ibis 137:371–378.

Ritland, K., and C. Ritland. 1996. Inferences about quantitative inheritance based on natural population structure in the yellow monkeyflower, Mimulus guttatus. Evolution 50:1074–1082.

Robinson, M. R., J. G. Pilkington, T. H. Clutton-Brock, J. M. Pemberton, and L. E. B. Kruuk. 2008. Environmental heterogeneity generates fluctuating selection on a secondary sexual trait. Current Biology 18:751–757.

Robinson, M. R., A. J. Wilson, J. G. Pilkington, T. H. Clutton-Brock, J. M. Pemberton, and L. E. B. Kruuk. 2009. The impact of environmental heterogeneity on genetic architecture in a wild population of soay sheep. Genetics 181:1639–1648.

Routley, M. B., and B. C. Husband. 2005. Responses to selection on male-phase duration in Chamerion angustifolium. Journal of Evolutionary Biology 18:1050–1059.

Sahli, H. F., J. K. Conner, F. H. Shaw, S. Howe, and A. Lale. 2008. Adaptive differentiation of quantitative traits in the globally distributed weed, wild radish (Raphanus raphanistrum). Genetics 180:945–955.

Saunders, S. P., and F. J. Cuthbert. 2014. Genetic and environmental influences on fitness-related traits in an endangered shorebird population. Biological Conservation 177:26–34.

Saura, M., P. Morán, S. Brotherstone, A. Caballero, J. Álvarez, and B. Villanueva. 2010. Predictions of response to selection caused by angling in a wild population of Atlantic salmon (Salmo salar). Freshwater Biology 55:923–930.

Schluter, D., and J. N. M. Smith. 1986. Genetic and phenotypic correlations in a natural population of song sparrows. Biological Journal of the Linnean Society 29:23–36.

Serbezov, D., L. Bernatchez, E. M. Olsen, and L. A. Vøllestad. 2010. Quantitative genetic parameters for wild stream-living brown trout: heritability and parental effects. Journal of Evolutionary Biology 23:1631–1641.

Sheldon, B., L. Kruuk, and J. Merila. 2003. Natural selection and inheritance of breeding time and clutch size in the collared flycatcher. Evolution 57:406–420.

Slate, J., P. M. Visscher, S. MacGregor, D. Stevens, M. L. Tate, and J. M. Pembertont. 2002. A genome scan for quantitative trait loci in a wild population of red deer (Cervus elaphus). Genetics 162:1863–1873.

Sletvold, N., P. Huttunen, R. Handley, K. Kärkkäinen, and J. Ågren. 2010. Cost of trichome production and resistance to a specialist insect herbivore in Arabidopsis lyrata. Evolutionary Ecology 24:1307–1319.

Smith, J. A., K. Wilson, J. G. Pilkington, and J. M. Pemberton. 1999. Heritable variation in resistance to gastro-intestinal nematodes in an unmanaged mammal population. Proceedings of the Royal Society B: Biological Sciences 266:1283–1290.

Smith, J. N. M. 1981. Does high fecundity reduce survival in song sparrows ? Evolution 35:1142–1148.

Smith, J. N. M., and A. A. Dhondt. 1980. Experimental confirmation of heritable morphological variation in a natural population of Song sparrows. Evolution 34:1155–1158.

Smith, J. N. M., and R. Zach. 1979. Heritability of some morphological characters in a song sparrow population. Evolution 33:460–467.

Smoker, W. W., A. J. Gharrett, and M. S. Stekoll. 1998. Genetic variation of return date in a population of pink salmon: a consequence of fluctuating environment and dispersive selection? Alaska Fishery Research Bulletin 5:46–54.

Smoker, W. W., A. J. Gharrett, M. S. Stekoll, and J. E. Joyce. 1994. Genetic analysis of size in an anadromous population of pink salmon. Canadian Journal of Fisheries and Aquatic Sciences 51:9–15.

Solberg, M. F., Ø. Skaala, F. Nilsen, and K. A. Glover. 2013. Does domestication cause changes in growth reaction norms? A study of farmed, wild and hybrid Atlantic salmon families exposed to environmental stress. PLoS ONE 8:e54469.

Sommer, S., and P. B. Pearman. 2003. Quantitative genetic analysis of larval life history traits in two alpine populations of Rana temporaria. Genetica 118:1–10.

Sorci  J. G. Swallow, T. Garland, Jr., J. Clobert, G. 1995. Quantitative genetics of locomoter speed and endurance in the lizard Lacerta vivipara. Physiological Zoology 68:698–720.

Stopher, K. V, C. a Walling, A. Morris, F. E. Guinness, T. H. Clutton-brock, J. M. Pemberton, and D. H. Nussey. 2012. Shared spatial effects on quantitative genetic parameters: accounting for spatial autocorrelation and home range overlap reduces estimates of heritability in wild red deer. Evolution 66:2411–2426.

Tarka, M., M. Akesson, D. Hasselquist, and B. Hansson. 2014. Intralocus sexual conflict over wing length in a wild migratory bird. The American naturalist 183:62–73.

Taylor, R. W., A. K. Boon, B. Dantzer, D. Réale, M. M. Humphries, S. Boutin, J. C. Gorrell, D. W. Coltman, and A. G. McAdam. 2012. Low heritabilities, but genetic and maternal correlations between red squirrel behaviours. Journal of Evolutionary Biology 25:614–624.

Teplitsky, C., J. A. Mills, J. S. Alho, J. W. Yarrall, and J. Merilä. 2008. Bergmann’s rule and climate change revisited: disentangling environmental and genetic responses in a wild bird population. Proceedings of the National Academy of Sciences of the United States of America 105:13492–13496.

Teplitsky, C., J. A. Mills, J. W. Yarrall, and J. Merilä. 2009. Heritability of fitness components in a wild bird population. Evolution 63:716–726.

Teplitsky, C., J. A. Mills, J. W. Yarrall, and J. Merilä. 2010. Indirect genetic effects in a sex-limited trait: the case of breeding time in red-billed gulls. Journal of Evolutionary Biology 23:935–944.

Thériault, V., D. Garant, L. Bernatchez, and J. J. Dodson. 2007. Heritability of life-history tactics and genetic correlation with body size in a natural population of brook charr (Salvelinus fontinalis). Journal of Evolutionary Biology 20:2266–2277.

Thessing, A., and J. Ekman. 1994. Selection on the genetical and environmental components of tarsal growth in juvenile willow tits (Parus montanus). Journal of Evolutionary Biology 7:713–726.

Thrower, F. P., J. J. Hard, and J. E. Joyce. 2004. Genetic architecture of growth and early life-history transitions in anadromous and derived freshwater populations of steelhead. Journal of Fish Biology 65:286–307.

Tschirren, B., J. Sendecka, T. G. G. Groothuis, L. Gustafsson, and B. Doligez. 2009. Heritable variation in maternal yolk hormone transfer in a wild bird population. American Naturalist 174:557–564.

Waldmann, P. 2001. Additive and non-additive genetic architecture of two different-sized populations of Scabiosa canescens. Heredity 86:648–657.

Waldmann, P., and S. Andersson. 1998. Comparison of quantitative genetic variation and allozyme diversity within and between populations of Scabiosa canescens and S-columbaria. Heredity 81:79–86.

Weber, A., and A. Kolb. 2014. Differences in heritable trait variation among populations of varying size in the perennial herb Phyteuma spicatum. Conservation Genetics:1329–1337.

Weller, S. G., A. K. Sakai, T. M. Culley, D. R. Campbell, P. Ngo, and A. K. Dunbar-Wallis. 2007. Sexually dimorphic inflorescence traits in a wind-pollinated species: heritabilities and genetic correlations in Schiedea adamantis (Caryophyllaceae). American Journal of Botany 94:1716–1725.

Widén, B., and S. Andersson. 1993. Quantitative genetics of life-history and morphology in a rare plant, Senecio integrifolius. Heredity 70:503–514.

Wilson, A. J., J. A. Hutchings, and M. M. Ferguson. 2003. Selective and genetic constraints on the evolution of body size in a stream-dwelling salmonid fish. Journal of Evolutionary Biology 16:584–594.

Wilson, A. J., L. E. B. Kruuk, and D. W. Coltman. 2005. Ontogenetic patterns in heritable variation for body size: using random regression models in a wild ungulate population. American Naturalist 166:E177–E192.

Wilson, A. J., M. B. Morrissey, M. J. Adams, C. A. Walling, F. E. Guinness, J. M. Pemberton, T. H. Clutton-Brock, and L. E. B. Kruuk. 2011. Indirect genetics effects and evolutionary constraint: an analysis of social dominance in red deer, Cervus elaphus. Journal of Evolutionary Biology 24:772–783.

Wilson, A. J., J. M. Pemberton, J. G. Pilkington, T. H. Clutton-Brock, D. W. Coltman, and L. E. B. Kruuk. 2007. Quantitative genetics of growth and cryptic evolution of body size in an island population. Evolutionary Ecology 21:337–356.

Wood, J. L. A., T. Dezel, D. Joyal, and D. J. Fraser. 2015. Population size is weakly related to quantitative genetic variation and trait differentiation in a stream fish. Evolution 69:2303-2318.

Zub, K., S. Piertney, P. A. Szafrańska, and M. Konarzewski. 2012. Environmental and genetic influences on body mass and resting metabolic rates (RMR) in a natural population of weasel Mustela nivalis. Molecular Ecology 21:1283–1293.
