# Supplementary material for: Are heritability and selection related to population size in nature? Meta‐analysis and conservation implications
Source: Evol Appl. 2016 Apr 3;9(5):640–57. doi: 10.1111/eva.12375 (PMC4869407; doi:10.1111/eva.12375)
Supplement: Supplementary file 4 — Appendix S4. Summary of h 2 and selection database characteristics. [file EVA-9-640-s004.docx]

Appendix D (Table D1). Summary of the characteristics of *h^2^* data from the pared dataset used in statistical analyses. The database includes studies of *h^2^* in natural populations from 1980-2014 for which *N* data was also available.

|  | | Number of items |
| --- | --- | --- |
| Studies | | 233 |
| Species | | 83 |
| Populations | | 146 |
| Records | | 1735 |
| Trait class: | |  |
| Morphology | | 1136 |
| Life history | | 446 |
| Other | | 153 |
| Taxon: | |  |
| Vertebrates (all) | | 1134 |
| Mammals | | 231 |
| Birds | | 561 |
| Fish | | 324 |
| Reptiles | | 7 |
| Amphibians | 11 | |
| Plants | | 601 |

Appendix D (Table D2). Summary of selection database characteristics. The database includes studies of phenotypic selection in natural populations from 1984-2014 for which *N* data was also available.

|  | Number of items |
| --- | --- |
| Studies | 133 |
| Species | 80 |
| Populations | 172 |
| Estimates (total) | 7344 |
| Linear gradients | 2784 |
| Linear differentials | 2425 |
| Quadratic gradients | 1187 |
| Quadratic differentials | 948 |
| Taxon: |  |
| Vertebrates (all) | 5177 |
| Mammals | 995 |
| Birds | 2895 |
| Fish | 1178 |
| Reptiles | 109 |
| Plants | 1782 |
| Invertebrates | 385 |

Appendix D (Table D3). Number of estimates of linear and quadratic selection as a function of taxon and trait type.

| Taxon | | Trait | |
| --- | --- | --- | --- |
| Linear selection gradients: | | | |
| Mammals | 255 | Morphology | 1535 |
| Birds | 1252 | Life history | 1104 |
| Fish | 301 | Principle component | 83 |
| Reptiles | 56 | Behaviour | 41 |
| Plants | 784 | Other | 21 |
| Invertebrates | 136 |  |  |
| Linear selection differentials: | | | |
| Mammals | 605 | Morphology | 1654 |
| Birds | 698 | Life history | 586 |
| Fish | 447 | Principle component | 142 |
| Reptiles | 29 | Behaviour | 31 |
| Plants | 550 | Other | 12 |
| Invertebrates | 96 |  |  |
| Quadratic selection gradients: | | | |
| Mammals | 60 | Morphology | 524 |
| Birds | 636 | Life history | 602 |
| Fish | 170 | Principle component | 35 |
| Reptiles | 17 | Behaviour | 6 |
| Plants | 231 | Other | 20 |
| Invertebrates | 73 |  |  |
| Quadratic selection differentials: | | | |
| Mammals | 75 | Morphology | 769 |
| Birds | 309 | Life history | 128 |
| Fish | 260 | Principle component | 24 |
| Reptiles | 7 | Behaviour | 24 |
| Plants | 217 | Other | 3 |
| Invertebrates | 80 |  |  |
