# Supplementary material for: Are heritability and selection related to population size in nature? Meta‐analysis and conservation implications
Source: Evol Appl. 2016 Apr 3;9(5):640–57. doi: 10.1111/eva.12375 (PMC4869407; doi:10.1111/eva.12375)
Supplement: Supplementary file 5 — Appendix S5. Reference list of studies included in the selection database. [file EVA-9-640-s005.docx]

Appendix E: List of studies included in the selection database.

Åkesson, M., S. Bensch, and D. Hasselquist. 2007. Genetic and phenotypic associations in morphological traits: a long term study of great reed warblers Acrocephalus arundinaceus. Journal of Avian Biology 38:58–72.

Alatalo, A. R. V, L. Gustafsson, and A. Lundberg. 1990. Phenotypic Selection on Heritable Size Traits: Environmental Variance and Genetic Response. American Naturalist 135:464–471.

Alcántara, J. M., J. M. Bastida, and P. J. Rey. 2010. Linking divergent selection on vegetative traits to environmental variation and phenotypic diversification in the Iberian columbines (Aquilegia). Journal of Evolutionary Biology 23:1218–1233.

Alcántara, J., P. Rey, F. Valera, A. M. Sanchez-Lafuente, and J. E. Gutierrez. 1997. Habitat alteration and plant intra-specific competition for seed dispersers. An example with Olea europaea var. sylvestris. Oikos 79:291–300.

Amon, T. R., and C. J. Foote. 2005. Concurrent natural and sexual selection in wild male sockeye salmon, Oncorhynchus nerka. Evolution 59:1104–1118.

Anderson, J. H., P. L. Faulds, W. I. Atlas, G. R. Pess, and T. P. Quinn. 2010. Selection on breeding date and body size in colonizing coho salmon, Oncorhynchus kisutch. Molecular ecology 19:2562–2573.

Arnqvist, G. 2015. Spatial variation in selective regimes: sexual selection in the water strider, Gerris odontogaster. Evolution 46:914–929.

Authier, M., E. Cam, and C. Guinet. 2011. Selection for increased body length in subantarctic fur seals on Amsterdam Island. Journal of Evolutionary Biology 24:607–616.

Badyaev, A., and T. Martin. 2000. Sexual dimorphism in relation to current selection in the house finch. Evolution 54:987–997.

Bailey, M. M., and M. T. Kinnison. 2010. Habitat-mediated size selection in endangered Atlantic salmon fry: selectional restoration assessment. Evolutionary Applications 3:352–362.

Barbraud, C. 2000. Natural selection on body size traits in a long-lived bird, the snow petrel Pagodroma nivea. Journal of Evolutionary Biology 13:81–88.

Bartkowska, M. P., and M. O. Johnston. 2012. Pollinators cause stronger selection than herbivores on floral traits in Lobelia cardinalis (Lobeliaceae). The New Phytologist 193:1039–1048.

Benkman, C., J. Smith, M. Maier, L. Hansen, and M. V. Talluto. 2013. Consistency and variation in phenotypic selection exerted by a community of seed predators. Evolution 67:157–169.

Bensch, S., H. Andrén, B. Hansson, H. C. Pedersen, H. Sand, D. Sejberg, P. Wabakken, M. Akesson, and O. Liberg. 2006. Selection for heterozygosity gives hope to a wild population of inbred wolves. PloS one 1:e72.

Berghe, E. Van Den, and M. Gross. 1989. Natural selection resulting from female breeding competition in a Pacific salmon (coho: Oncorhynchus kisutch). Evolution 43:125–140.

Bittner, D., L. Excoffier, and C. R. Largiadèr. 2010. Patterns of morphological changes and hybridization between sympatric whitefish morphs (Coregonus spp.) in a Swiss lake: a role for eutrophication? Molecular ecology 19:2152–2167.

Björklund, M., and M. Lindén. 1993. Sexual size dimorphism in the great tit (Parus major) in relation to history and current selection. Journal of Evolutionary Biology 6:397–415.

Bouwhuis, S., J. L. Quinn, B. C. Sheldon, and S. Verhulst. 2014. Personality and basal metabolic rate in a wild bird population. Oikos 123:56–62.

Boyce, M. S., and C. M. Perrins. 1987. Optimizing great tit clutch size in a fluctuating environment. Ecology 68:142–153.

Brommer, J. E., J. Merila, B. C. Shedldon, and L. Gustafsson. 2005. Natural selection and genetic variation for reproductive reaction norms in a wild bird population. Evolution 59:1362–1371.

Brommer, J. E., and K. Rattiste. 2008. “Hidden” reproductive conflict between mates in a wild bird population. Evolution 62:2326–2333.

Brown, G. P., and P. J. Weatherhead. 1999. Female distribution affects mate searching and sexual selection in male northern water snakes (Nerodia sipedon). Behavioral Ecology and Sociobiology 47:9–16.

Carlson, S. M., E. Edeline, L. Asbjørn Vøllestad, T. O. Haugen, I. J. Winfield, J. M. Fletcher, J. Ben James, and N. C. Stenseth. 2007. Four decades of opposing natural and human-induced artificial selection acting on Windermere pike (Esox lucius). Ecology Letters 10:512–521.

Carlson, S. M., E. M. Olsen, and L. A. Vllestad. 2008. Seasonal mortality and the effect of body size: a review and an empirical test using individual data on brown trout. Functional Ecology 22:663–673.

Carlson, S. M., and T. P. Quinn. 2007. Ten years of varying lake level and selection on size-at-maturity in sockeye salmon. Ecology 88:2620–2629.

Carlson, S. M., H. B. Rich, and T. P. Quinn. 2009. Does variation in selection imposed by bears drive divergence among populations in the size and shape of sockeye salmon? Evolution 63:1244–1261.

Caruso, C. M. 2000. Competition for pollination influences selection on floral traits of Ipomopsis aggregata. Evolution 54:1546–1557.

Caruso, C. M., S. B. Peterson, and C. Ridley. 2003. Natural selection of floral traits of Lobelia (Lobeliaceae): spatial and temporal variation. American Journal of Botany 90:1333–1340.

Castellanos, M. C., J. M. Alcántara, P. J. Rey, and J. M. Bastida. 2011. Intra-population comparison of vegetative and floral trait heritabilities estimated from molecular markers in wild Aquilegia populations. Molecular Ecology 20:3513–3524.

Castilla, A. R., C. Alonso, and C. M. Herrera. 2013. Herbivory at marginal populations: Consequences for maternal fitness and vegetative differentiation. Acta Oecologica 49:32–38.

Castilla, A. R., C. Alonso, and C. M. Herrera. 2015. Sex-specific phenotypic selection and geographic variation in gender divergence in a gynodioecious shrub. Plant Biology 17:186–193.

Charmantier, A., L. E. B. Kruuk, J. Blondel, and M. M. Lambrechts. 2004. Testing for microevolution in body size in three blue tit populations. Journal of Evolutionary Biology 17:732–743.

Charmantier, A., R. H. Mccleery, L. R. Cole, C. Perrins, L. E. B. Kruuk, and B. C. Sheldon. 2008. Adaptive phenotypic plasticity in response to climate change in a wild bird population. Science 320:800–803.

Charmantier, A., C. Perrins, R. H. McCleery, and B. C. Sheldon. 2006. Evolutionary response to selection on clutch size in a long‐term study of the mute swan. The American Naturalist 167:453–465.

Cintrón-Berdecía, S., and R. Tremblay. 2006. Spatial variation in phenotypic selection on floral characteristics in an epiphytic orchid. Folia Geobotanica 41:33–46.

Clegg, S. M., F. D. Frentiu, J. Kikkawa, G. Tavecchia, and I. P. F. Owens. 2008. 4000 years of phenotypic change in an island bird: heterogeneity of selection over three microevolutionary timescales. Evolution 62:2393–2410.

Coltman, D. W., P. O’Donoghue, J. T. Hogg, and M. Festa-Bianchet. 2005. Selection and genetic (co) variance in bighorn sheep. Evolution 59:1372–1382.

Coulson, T., L. Kruuk, G. Tavecchia, J. M. Pemberton, and T. H. Clutton-Brock. 2003. Estimating selection on neonatal traits in red deer using elasticity path analysis. Evolution 57:2879–2892.

Descamps, S., M. R. Forbes, H. G. Gilchrist, O. P. Love, and J. Bêty. 2011. Avian cholera, post-hatching survival and selection on hatch characteristics in a long-lived bird, the common eider Somateria mollisima. Journal of Avian Biology 42:39–48.

DiBattista, J. D., K. A. Feldheim, D. Garant, S. H. Gruber, and A. P. Hendry. 2011. Anthropogenic disturbance and evolutionary parameters: a lemon shark population experiencing habitat loss. Evolutionary Applications 4:1–17.

Dibattista, J. D., K. A. Feldheim, S. H. Gruber, and A. P. Hendry. 2007. When bigger is not better: selection against large size, high condition and fast growth in juvenile lemon sharks. Journal of Evolutionary Biology 20:201–212.

Dieringer, G., and R. L. Cabreara. 1994. Sexual selection of anther trichomes and sexual dimorphism in Ibervillea lindheimeri (Cucurbitaceae: Melothrieae). American Journal of Botany 81:111–118.

Dugdale, H. L., P. Nouvellet, L. C. Pope, T. Burke, and D. W. Macdonald. 2010. Fitness measures in selection analyses: sensitivity to the overall number of offspring produced in a lifetime. Journal of Evolutionary Biology 23:282–292.

Ferguson, I. M., and D. J. Fairbairn. 2000. Sex-specific selection and sexual size dimorphism in the waterstrider Aquarius remigis. Journal of Evolutionary Biology 13:160–170.

Ferguson, I. M., and D. J. Fairbairn. 2001. Is selection ready when opportunity knocks ? Evolutionary Ecology Research 3:199–207.

Fletcher, Q. E., J. R. Speakman, S. Boutin, J. E. Lane, A. G. McAdam, J. C. Gorrell, D. W. Coltman, and M. M. Humphries. 2014. Daily energy expenditure during lactation is strongly selected in a free-living mammal. Functional Ecology:DOI: 10.1111/1365–2435.12313.

Frentiu, F. D., S. M. Clegg, M. W. Blows, and I. P. F. Owens. 2007. Large body size in an island-dwelling bird: a microevolutionary analysis. Journal of Evolutionary Biology 20:639–649.

Garant, D., L. E. B. Kruuk, R. H. McCleery, and B. C. Sheldon. 2007. The effects of environmental heterogeneity on multivariate selection on reproductive traits in female great tits. Evolution 61:1546–1559.

Garant, D., L. Kruuk, R. McCleery, and B. Sheldon. 2004. Evolution in a changing environment: a case study with great tit fledging mass. The American Naturalist 164:E115–E129.

Gibbs, H. L. 1988. Heritability and Selection on Clutch Size in Darwin’s Medium Ground Finches (Geospiza fortis). Evolution 42:750–762.

Gienapp, P., and T. Bregnballe. 2012. Fitness consequences of timing of migration and breeding in cormorants. PloS one 7:e46165.

Gienapp, P., E. Postma, and M. Visser. 2006. Why breeding time has not responded to selection for earlier breeding in a songbird population. Evolution 60:2381–2388.

Gómez, J. M., F. Perfectti, J. P. M. Camacho, F. Perfectti, and J. P. M. Camacho. 2015. Natural selection on erysimum mediohispanicum flower shape: insights into the evolution of zygomorphy. The American Naturalist 168:531–545.

Goodenough, A. E., A. G. Hart, and S. L. Elliot. 2011. What prevents phenological adjustment to climate change in migrant bird species? Evidence against the “arrival constraint” hypothesis. International Journal of Biometeorology 55:97–102.

Grant, B., and P. Grant. 1993. Evolution of Darwin’s finches caused by a rare climatic event. Proceedings of the Royal Society B: Biological Sciences 251:111–117.

Grant, B. R. 1985. Selection on bill characters in a population of Darwin’s finches: Geospiza conirostris on Isla Genovesa, Galapagos. Evolution 39:523–532.

Grant, B. R., and P. R. Grant. 1989. Natural selection in a population of Darwin’s finches. The American Naturalist 133:377–393.

Grant, P. R., and B. R. Grant. 1995. Predicting microevolutionary responses to directional selection on heritable variation. Evolution 49:241–251.

Grant, P. R., and B. R. Grant. 2002. Unpredictable evolution in a 30-year study of Darwin’s finches. Science 296:707–711.

Hodgins, K. a, and S. C. H. Barrett. 2008. Natural selection on floral traits through male and female function in wild populations of the heterostylous daffodil Narcissus triandrus. Evolution 62:1751–1763.

Holleley, C. E., C. R. Dickman, M. S. Crowther, and B. P. Oldroyd. 2006. Size breeds success: multiple paternity, multivariate selection and male semelparity in a small marsupial, Antechinus stuartii. Molecular Ecology 15:3439–3448.

Husby, A., M. E. Visser, and L. E. B. Kruuk. 2011. Speeding up microevolution: the effects of increasing temperature on selection and genetic variance in a wild bird population. PLoS Biology 9:e1000585.

Johnston, F., and J. Post. 2009. Density-dependent life-history compensation of an iteroparous salmonid. Ecological Applications 19:449–467.

Kalisz, S. 1986. Variable selection on the timing of germination in Collinsia verna (Scrophulariaceae). Evolution 40:479–491.

Kim, S.-Y., J. A. Fargallo, P. Vergara, and J. Martínez-Padilla. 2013. Multivariate heredity of melanin-based coloration, body mass and immunity. Heredity 111:139–146.

Kleunen, M., and K. Ritland. 2004. Predicting evolution of floral traits associated with mating system in a natural plant population. Journal of Evolutionary Biology 17:1389–1399.

Van Kleunen, M. 2006. Adaptive genetic differentiation in life-history traits between populations of Mimulus guttatus with annual and perennial life-cycles. Evolutionary Ecology 21:185–199.

Kodama, M., J. J. Hard, and K. A. Naish. 2012. Temporal variation in selection on body length and date of return in a wild population of coho salmon, Oncorhynchus kisutch. BMC Evolutionary Biology 12.

Koskinen, M., T. Haugen, and C. Primmer. 2002. Contemporary fisherian life-history evolution in small salmonid populations. Nature 419:826–830.

Kruuk, L., J. Merilä, and B. Sheldon. 2001. Phenotypic selection on a heritable size trait revisited. The American Naturalist 158:557–571.

Kruuk, L., J. Slate, and J. Pemberton. 2002. Antler size in red deer: heritability and selection but no evolution. Evolution 56:1683–1695.

Lane, J. E., S. Boutin, M. R. Gunn, and D. W. Coltman. 2009. Sexually selected behaviour: red squirrel males search for reproductive success. The Journal of Animal Ecology 78:296–304.

Larivée, M. L., S. Boutin, J. R. Speakman, A. G. McAdam, and M. M. Humphries. 2010. Associations between over-winter survival and resting metabolic rate in juvenile North American red squirrels. Functional Ecology 24:597–607.

Lea, A. J., D. T. Blumstein, T. W. Wey, and J. G. A. Martin. 2010. Heritable victimization and the benefits of agonistic relationships. Proceedings of the National Academy of Sciences of the United States of America 107:21587–21592.

Lindén, M., L. Gustafsson, and T. Pärt. 1992. Selection on fledging mass in the collared flycatcher and the great tit. Ecology 73:336–343.

Maad, J. 2000. Phenotypic selection in hawkmoth‐pollinated Platanthera bifolia: targets and fitness surfaces. Evolution 54:112–123.

Maad, J., and R. Alexandersson. 2004. Variable selection in Platanthera bifolia (Orchidaceae): phenotypic selection differed between sex functions in a drought year. Journal of Evolutionary Biology 17:642–650.

Madsen, T., and R. Shine. 1993. Temporal variability in sexual selection acting on reproductive tactics and body size in male snakes. American Naturalist 141:167–171.

McAdam, A., and S. Boutin. 2003. Variation in viability selection among cohorts of juvenile red squirrels (Tamiasciurus hudsonicus). Evolution 57:1689–1697.

McAdam, A. G., and S. Boutin. 2004. Maternal effects and the response to selection in red squirrels. Proceedings of the Royal Society B: Biological Sciences 271:75–79.

Medel, R. 2000. Assessment of parasite-mediated selection in a host-parasite system in plants. Ecology 81:1554–1564.

Mihoub, J. B., N. G. Mouawad, P. Pilard, F. Jiguet, M. Low, and C. Teplitsky. 2012. Impact of temperature on the breeding performance and selection patterns in lesser kestrels Falco naumanni. Journal of Avian Biology 43:472–480.

Milner, J. M., S. D. Albon, A. W. Illius, J. M. Pemberton, and T. H. Clutton-Brock. 1999. Repeated selection of morphometric traits in the Soay sheep on St Kilda. Journal of Animal Ecology 68:472–488.

Mitchell-Olds, T., and J. Bergelson. 1990. Statistical genetics of an annual plant, Impatiens capensis. II. Natural selection. Genetics 124:417–421.

Nussey, D. H., T. H. Clutton-Brock, D. a. Elston, S. D. Albon, and L. E. B. Kruuk. 2005. Phenotypic plasticity in a maternal trait in red deer. Journal of Animal Ecology 74:387–396.

O’Neil, P. 1997. Natural selection on genetically correlated phenological characters in Lythrum salicaria L.(Lythraceae). Evolution 51:267–274.

Olsson, M., and T. Madsen. 2001. Between‐year variation in determinants of offspring survival in the Sand Lizard, Lacerta agilis. Functional Ecology 15:443–450.

Parker, T. H., T. a Wilkin, I. R. Barr, B. C. Sheldon, L. Rowe, and S. C. Griffith. 2011. Fecundity selection on ornamental plumage colour differs between ages and sexes and varies over small spatial scales. Journal of Evolutionary Biology 24:1584–1597.

Parra-Tabla, V., and C. F. Vargas. 2004. Phenology and phenotypic natural selection on the flowering time of a deceit-pollinated tropical orchid, Myrmecophila christinae. Annals of Botany 94:243–250.

Pelletier, F., D. Réale, D. Garant, D. W. Coltman, and M. Festa-Bianchet. 2007. Selection on heritable seasonal phenotypic plasticity of body mass. Evolution 61:1969–1979.

Poissant, J., A. J. Wilson, M. Festa-Bianchet, J. T. Hogg, and D. W. Coltman. 2008. Quantitative genetics and sex-specific selection on sexually dimorphic traits in bighorn sheep. Proceedings of the Royal Society B: Biological Sciences 275:623–628.

Postma, E., and A. van Noordwijk. 2005. Gene flow maintains a large genetic difference in clutch size at a small spatial scale. Nature 433:65–68.

Preziosi, R. F., and D. J. Fairbairn. 1996. Sexual size dimorphism and selection in the wild in the waterstrider Aquarius remigis: body size, components of body size and male mating success. Journal of Evolutionary Biology 9:317–336.

Preziosi, R., and D. Fairbairn. 1997. Sexual size dimorphism and selection in the wild in the waterstrider Aquarius remigis: lifetime fecundity selection on female total length and its components. Evolution 51:467–474.

Price, T. 1984a. Sexual selection on body size, territory and plumage variables in a population of Darwin’s finches. Evolution 38:327–341.

Price, T. 1984b. The evolution of sexual size dimorphism in Darwin’s finches. American Naturalist 123:500–518.

Price, T., P. Grant, H. Gibbs, and P. Boag. 1984. Recurrent patterns of natural selection in a population of Darwin’s finches. Nature 309:787–789.

Réale, D., D. Berteaux, A. McAdam, and S. Boutin. 2003. Lifetime selection on heritable life‐history traits in a natural population of red squirrels. Evolution 57:2416–2423.

Réale, D., and M. Festa-Bianchet. 2003. Predator-induced natural selection on temperament in bighorn ewes. Animal Behaviour 65:463–470.

Reed, T. E., S. Wanless, M. P. Harris, M. Frederiksen, L. E. B. Kruuk, and E. J. A. Cunningham. 2006. Responding to environmental change: plastic responses vary little in a synchronous breeder. Proceedings of the Royal Society B: Biological Sciences 273:2713–2719.

Reed, T. E., P. Warzybok, A. J. Wilson, R. W. Bradley, S. Wanless, and W. J. Sydeman. 2009. Timing is everything: flexible phenology and shifting selection in a colonial seabird. The Journal of Animal Ecology 78:376–387.

Sánchez-Lafuente, A. M., and R. Parra. 2009. Implications of a long-term, pollinator-mediated selection on floral traits in a generalist herb. Annals of Botany 104:689–701.

Sandring, S., and J. Agren. 2009. Pollinator-mediated selection on floral display and flowering time in the perennial herb Arabidopsis lyrata. Evolution 63:1292–1300.

Sandring, S., M. Riihimäki, O. Savolainen, and J. Agren. 2007. Selection on flowering time and floral display in an alpine and a lowland population of Arabidopsis lyrata. Journal of Evolutionary Biology 20:558–567.

Schluter, D., and J. Smith. 1986. Natural selection on beak and body size in the song sparrow. Evolution 40:221–231.

Seamons, T., P. Bentzen, and T. P. Quinn. 2007. DNA parentage analysis reveals inter-annual variation in selection: results from 19 consecutive brood years in steelhead trout. Evolutionary Ecology Research 9:409–431.

Serbezov, D., L. Bernatchez, E. M. Olsen, and L. A. Vøllestad. 2010. Mating patterns and determinants of individual reproductive success in brown trout (Salmo trutta) revealed by parentage analysis of an entire stream living population. Molecular Ecology 19:3193–3205.

Sheldon, B., L. Kruuk, and J. Merila. 2003. Natural selection and inheritance of breeding time and clutch size in the collared flycatcher. Evolution 57:406–420.

Sletvold, N., and J. Ågren. 2010. Pollinator-mediated selection on floral display and spur length in the orchid Gymnadenia conopsea. International Journal of Plant Sciences 171:999–1009.

Sletvold, N., J. Grindeland, and J. Ågren. 2013. Vegetation context influences the strength and targets of pollinator-mediated selection in a deceptive orchid. Ecology 94:1236–1242.

Sletvold, N., J. M. Grindeland, and J. Agren. 2010. Pollinator-mediated selection on floral display, spur length and flowering phenology in the deceptive orchid Dactylorhiza lapponica. The New Phytologist 188:385–392.

Steiger, S., G. Ower, J. Stoki, C. Mitchell, J. Hunt, and S. K. Sakaluk. 2013. Sexual selection on cuticular hydrocarbons of male sagebrush crickets in the wild. Proceedings of the Royal Society B: Biological Sciences 280.

Sun, H.-Q., R. Alexandersson, and S. Ge. 2010. Positive effects of flower abundance and synchronous flowering on pollination success, and pollinia dispersal in rewardless Changnienia amoena (Orchidaceae). Biological Journal of the Linnean Society 99:477–488.

Svanbäck, R., and L. Persson. 2009. Population density fluctuations change the selection gradient in Eurasian perch. The American Naturalist 173:507–16.

Tarka, M., M. Akesson, D. Hasselquist, and B. Hansson. 2014. Intralocus sexual conflict over wing length in a wild migratory bird. The American naturalist 183:62–73.

Taylor, R. W., S. Boutin, M. M. Humphries, and a G. McAdam. 2014. Selection on female behaviour fluctuates with offspring environment. Journal of Evolutionary Biology 27:2308–2321.

Teplitsky, C., J. A. Mills, J. S. Alho, J. W. Yarrall, and J. Merilä. 2008. Bergmann’s rule and climate change revisited: disentangling environmental and genetic responses in a wild bird population. Proceedings of the National Academy of Sciences of the United States of America 105:13492–13496.

Teplitsky, C., J. A. Mills, J. W. Yarrall, and J. Merilä. 2010. Indirect genetic effects in a sex-limited trait: the case of breeding time in red-billed gulls. Journal of Evolutionary Biology 23:935–944.

Teplitsky, C., M. Tarka, A. P. Møller, S. Nakagawa, J. Balbontín, T. a Burke, C. Doutrelant, A. Gregoire, B. Hansson, D. Hasselquist, L. Gustafsson, F. de Lope, A. Marzal, J. A. Mills, N. T. Wheelwright, J. W. Yarrall, and A. Charmantier. 2014. Assessing multivariate constraints to evolution across ten long-term avian studies. PloS one 9:e90444.

Thessing, A., and J. Ekman. 1994. Selection on the genetical and environmental components of tarsal growth in juvenile willow tits (Parus montanus). Journal of Evolutionary Biology 7:713–726.

Tschirren, B., E. Postma, L. Gustafsson, T. G. G. Groothuis, and B. Doligez. 2014. Natural selection acts in opposite ways on correlated hormonal mediators of prenatal maternal effects in a wild bird population. Ecology Letters 17:1310–1315.

Walling, C. A., M. B. Morrissey, K. Foerster, T. H. Clutton-Brock, J. M. Pemberton, and L. E. B. Kruuk. 2014. A multivariate analysis of genetic constraints to life history evolution in a wild population of red deer. Genetics 198:1735–1749.

Weber, A., and A. Kolb. 2010. Evolutionary consequences of habitat fragmentation: population size and density affect selection on inflorescence size in a perennial herb. Evolutionary Ecology 25:417–428.

Weber, A., and A. Kolb. 2013. Population size, pollination and phenotypic trait selection in Phyteuma spicatum. Acta Oecologica 47:46–51.

Weese, D. J., S. P. Gordon, A. P. Hendry, and M. T. Kinnison. 2010. Spatiotemporal variation in linear natural selection on body color in wild guppies (Poecilia reticulata). Evolution 64:1802–1815.

Widén, B. 1991. Phenotypic selection on flowering phenology in Senecio integrifolius, a perennial herb. Oikos 61:205–215.

Willis, J. 1996. Measures of phenotypic selection are biased by partial inbreeding. Evolution 50:1501–1511.

Wilson, A. J., J. A. Hutchings, and M. M. Ferguson. 2003. Selective and genetic constraints on the evolution of body size in a stream-dwelling salmonid fish. Journal of Evolutionary Biology 16:584–594.

Wilson, A. J., J. M. Pemberton, J. G. Pilkington, D. W. Coltman, D. V. Mifsud, T. H. Clutton-Brock, and L. E. B. Kruuk. 2006. Environmental coupling of selection and heritability limits evolution. PLoS Biology 4:e216.

Yeh, P., and T. Price. 2004. Adaptive phenotypic plasticity and the successful colonization of a novel environment. The American Naturalist 164:531–542.

Zhang, B., and Q. J. Li. 2014. Phenotypic selection on the staminal lever mechanism in Salvia digitaloides (Labiaceae). Evolutionary Ecology 28:373–386.
