# Supplementary material for: Are heritability and selection related to population size in nature? Meta‐analysis and conservation implications
Source: Evol Appl. 2016 Apr 3;9(5):640–57. doi: 10.1111/eva.12375 (PMC4869407; doi:10.1111/eva.12375)
Supplement: Supplementary file 6 — Appendix S6. Results of models to investigate the effect of N on h 2 data using MCMCglmm. [file EVA-9-640-s006.docx]

Appendix F (Table F1). Results of unweighted models to investigate the effect of *N* on *h^2^* data using MCMCglmm. Models included *h^2^* data for bird populations.

| Trait class | Intercept | Fixed effect | Posterior mode | l-95% CI | u-95% CI | *P_MCMC_* |
| --- | --- | --- | --- | --- | --- | --- |
| Life history (All data) | ANOVA | (Intercept) | 0.324 | 0.216 | 0.421 | <0.001 |
|  |  | *N* | -0.000452 | -0.0120 | 0.0102 | 0.857 |
|  |  | Trait class (MO) | 0.123 | 0.0567 | 0.191 | 0.00100 |
|  |  | Trait class (O) | 0.0608 | -0.0234 | 0.152 | 0.155 |
|  |  | Analysis type (Bayesian) | -0.109 | -0.244 | -0.0124 | 0.0340 |
|  |  | Analysis type (P-O regression) | -0.00508 | -0.113 | 0.119 | 0.954 |
|  |  | Analysis type (REML) | -0.0861 | -0.192 | 0.00500 | 0.0605 |
|  | PO-regression | (Intercept) | 0.307 | 0.223 | 0.399 | <0.001 |
|  |  | *N* | -0.000689 | -0.0115 | 0.0102 | 0.874 |
|  |  | Trait class (MO) | 0.131 | 0.0572 | 0.193 | 0.000400 |
|  |  | Trait class (O) | 0.0538 | -0.0202 | 0.153 | 0.143 |
|  |  | Analysis type (ANOVA) | -0.00138 | -0.109 | 0.121 | 0.941 |
|  |  | Analysis type (Bayesian) | -0.124 | -0.216 | -0.0139 | 0.0202 |
|  |  | Analysis type (REML) | -0.0876 | -0.168 | -0.0144 | 0.0190 |
|  | REML | (Intercept) | 0.218 | 0.158 | 0.282 | <0.001 |
|  |  | *N* | 0.00114 | -0.0118 | 0.0101 | 0.860 |
|  |  | Trait class (MO) | 0.115 | 0.0550 | 0.191 | 0.000400 |
|  |  | Trait class (O) | 0.0617 | -0.0262 | 0.153 | 0.157 |
|  |  | Analysis type (ANOVA) | 0.101 | -0.00551 | 0.193 | 0.0594 |
|  |  | Analysis type (Bayesian) | -0.0273 | -0.106 | 0.0487 | 0.477 |
|  |  | Analysis type (P-O regression) | 0.0950 | 0.0169 | 0.171 | 0.0248 |
|  | Bayesian | (Intercept) | 0.193 | 0.110 | 0.267 | <0.001 |
|  |  | *N* | -0.000948 | -0.0119 | 0.0100 | 0.876 |
|  |  | Trait class (MO) | 0.121 | 0.0584 | 0.196 | <0.001 |
|  |  | Trait class (O) | 0.0670 | -0.0232 | 0.151 | 0.142 |
|  |  | Analysis type (ANOVA) | 0.138 | 0.00822 | 0.242 | 0.0385 |
|  |  | Analysis type (P-O regression) | 0.118 | 0.0215 | 0.220 | 0.0200 |
|  |  | Analysis type (REML) | 0.0301 | -0.0532 | 0.104 | 0.474 |
| Morphology (All data) | ANOVA | (Intercept) | 0.448 | 0.343 | 0.537 | <0.001 |
|  |  | *N* | -0.000215 | -0.0114 | 0.0105 | 0.885 |
|  |  | Trait class (LH) | -0.118 | -0.193 | -0.0552 | 0.000200 |
|  |  | Trait class (O) | -0.0623 | -0.139 | 0.0205 | 0.150 |
|  |  | Analysis type (Bayesian) | -0.126 | -0.242 | -0.00944 | 0.0346 |
|  |  | Analysis type (P-O regression) | 0.0160 | -0.117 | 0.114 | 0.933 |
|  |  | Analysis type (REML) | -0.0903 | -0.198 | 0.000150 | 0.0588 |
|  | PO-regression | (Intercept) | 0.439 | 0.367 | 0.506 | <0.001 |
|  |  | *N* | -0.00109 | -0.0120 | 0.00967 | 0.875 |
|  |  | Trait class (LH) | -0.124 | -0.192 | -0.0550 | 0.000600 |
|  |  | Trait class (O) | -0.0470 | -0.138 | 0.0215 | 0.154 |
|  |  | Analysis type (ANOVA) | -0.00229 | -0.110 | 0.117 | 0.928 |
|  |  | Analysis type (Bayesian) | -0.113 | -0.220 | -0.0195 | 0.0202 |
|  |  | Analysis type (REML) | -0.104 | -0.169 | -0.0154 | 0.0212 |
|  | REML | (Intercept) | 0.341 | 0.299 | 0.386 | <0.001 |
|  |  | *N* | -0.000797 | -0.0117 | 0.0100 | 0.868 |
|  |  | Trait class (LH) | -0.116 | -0.192 | -0.0567 | 0.000400 |
|  |  | Trait class (O) | -0.0611 | -0.138 | 0.0228 | 0.157 |
|  |  | Analysis type (ANOVA) | 0.120 | -0.00345 | 0.198 | 0.0626 |
|  |  | Analysis type (Bayesian) | -0.0443 | -0.110 | 0.0435 | 0.459 |
|  |  | Analysis type (P-O regression) | 0.0986 | 0.0149 | 0.167 | 0.0218 |
|  | Bayesian | (Intercept) | 0.315 | 0.236 | 0.395 | <0.001 |
|  |  | *N* | -0.000674 | -0.0119 | 0.00982 | 0.890 |
|  |  | Trait class (LH) | -0.122 | -0.192 | -0.0556 | 0.000400 |
|  |  | Trait class (O) | -0.0776 | -0.140 | 0.0198 | 0.143 |
|  |  | Analysis type (ANOVA) | 0.130 | 0.00797 | 0.235 | 0.0324 |
|  |  | Analysis type (P-O regression) | 0.116 | 0.0205 | 0.220 | 0.0178 |
|  |  | Analysis type (REML) | 0.0350 | -0.0499 | 0.106 | 0.477 |
| Other (All data) | ANOVA | (Intercept) | 0.382 | 0.267 | 0.497 | <0.001 |
|  |  | *N* | -0.00313 | -0.0116 | 0.0102 | 0.860 |
|  |  | Trait class (LH) | -0.0699 | -0.153 | 0.0223 | 0.152 |
|  |  | Trait class (MO) | 0.0487 | -0.0203 | 0.139 | 0.153 |
|  |  | Analysis type (Bayesian) | -0.120 | -0.242 | -0.00619 | 0.0396 |
|  |  | Analysis type (P-O regression) | -0.00205 | -0.119 | 0.110 | 0.932 |
|  |  | Analysis type (REML) | -0.0970 | -0.194 | 0.00680 | 0.0620 |
|  | PO-regression | (Intercept) | 0.365 | 0.270 | 0.474 | <0.001 |
|  |  | *N* | -0.000101 | -0.0117 | 0.0102 | 0.866 |
|  |  | Trait class (LH) | -0.0656 | -0.157 | 0.0199 | 0.147 |
|  |  | Trait class (MO) | 0.0617 | -0.0221 | 0.139 | 0.154 |
|  |  | Analysis type (ANOVA) | -0.0114 | -0.110 | 0.115 | 0.930 |
|  |  | Analysis type (Bayesian) | -0.119 | -0.219 | -0.0164 | 0.0178 |
|  |  | Analysis type (REML) | -0.0937 | -0.168 | -0.0145 | 0.0198 |
|  | REML | (Intercept) | 0.276 | 0.211 | 0.355 | <0.001 |
|  |  | *N* | -0.00209 | -0.0117 | 0.0102 | 0.869 |
|  |  | Trait class (LH) | -0.0534 | -0.155 | 0.0249 | 0.154 |
|  |  | Trait class (MO) | 0.0610 | -0.0206 | 0.141 | 0.152 |
|  |  | Analysis type (ANOVA) | 0.105 | -0.00254 | 0.194 | 0.0552 |
|  |  | Analysis type (Bayesian) | -0.0295 | -0.109 | 0.0483 | 0.470 |
|  |  | Analysis type (P-O regression) | 0.0877 | 0.0178 | 0.169 | 0.0208 |
|  | Bayesian | (Intercept) | 0.248 | 0.167 | 0.343 | <0.001 |
|  |  | *N* | -0.00201 | -0.0118 | 0.0101 | 0.873 |
|  |  | Trait class (LH) | -0.0557 | -0.151 | 0.0224 | 0.154 |
|  |  | Trait class (MO) | 0.0514 | -0.0215 | 0.137 | 0.148 |
|  |  | Analysis type (ANOVA) | 0.132 | 0.0121 | 0.247 | 0.0402 |
|  |  | Analysis type (P-O regression) | 0.113 | 0.0127 | 0.216 | 0.0194 |
|  |  | Analysis type (REML) | 0.0347 | -0.0474 | 0.108 | 0.469 |

Appendix F (Table F2). Results of meta-analysis and unweighted models to investigate the effect of *N* on *h^2^* data using MCMCglmm. Models excluded *h^2^* data for bird populations.

| Trait class | Intercept | Fixed effect | Posterior mode | l-95% CI | u-95% CI | *P_MCMC_* |
| --- | --- | --- | --- | --- | --- | --- |
| Life history (SE) | ANOVA | (Intercept) | 0.342 | 0.183 | 0.468 | <0.001 |
|  |  | *N* | 0.00305 | -0.00961 | 0.0182 | 0.605 |
|  |  | Trait class (MO) | 0.0514 | -0.0489 | 0.154 | 0.307 |
|  |  | Trait class (O) | -0.0398 | -0.179 | 0.0758 | 0.432 |
|  |  | Analysis type (Bayesian) | -0.130 | -0.287 | 0.0272 | 0.104 |
|  |  | Analysis type (REML) | -0.0630 | -0.193 | 0.0816 | 0.384 |
|  | REML | (Intercept) | 0.258 | 0.175 | 0.356 | <0.001 |
|  |  | *N* | 0.00544 | -0.0104 | 0.0175 | 0.579 |
|  |  | Trait class (MO) | 0.0654 | -0.0451 | 0.153 | 0.280 |
|  |  | Trait class (O) | -0.0506 | -0.176 | 0.0831 | 0.440 |
|  |  | Analysis type (ANOVA) | 0.0421 | -0.0707 | 0.210 | 0.385 |
|  |  | Analysis type (Bayesian) | -0.0623 | -0.173 | 0.0340 | 0.192 |
|  | Bayesian | (Intercept) | 0.187 | 0.0888 | 0.309 | 0.00220 |
|  |  | *N* | 0.00317 | -0.0103 | 0.0177 | 0.597 |
|  |  | Trait class (MO) | 0.0468 | -0.0464 | 0.154 | 0.296 |
|  |  | Trait class (O) | -0.0295 | -0.179 | 0.0738 | 0.419 |
|  |  | Analysis type (ANOVA) | 0.133 | -0.0348 | 0.282 | 0.110 |
|  |  | Analysis type (REML) | 0.0681 | -0.0324 | 0.172 | 0.188 |
| Morphology (SE) | ANOVA | (Intercept) | 0.375 | 0.241 | 0.512 | <0.001 |
|  |  | *N* | 0.00428 | -0.0108 | 0.0171 | 0.598 |
|  |  | Trait class (LH) | -0.0712 | -0.160 | 0.0442 | 0.290 |
|  |  | Trait class (O) | -0.123 | -0.217 | 0.0160 | 0.0900 |
|  |  | Analysis type (Bayesian) | -0.101 | -0.285 | 0.0355 | 0.117 |
|  |  | Analysis type (REML) | -0.0572 | -0.197 | 0.0811 | 0.396 |
|  | REML | (Intercept) | 0.318 | 0.261 | 0.371 | <0.001 |
|  |  | *N* | 0.00356 | -0.0104 | 0.0176 | 0.602 |
|  |  | Trait class (LH) | -0.0500 | -0.151 | 0.0474 | 0.295 |
|  |  | Trait class (O) | -0.100 | -0.219 | 0.0156 | 0.104 |
|  |  | Analysis type (ANOVA) | 0.0758 | -0.0797 | 0.194 | 0.389 |
|  |  | Analysis type (Bayesian) | -0.0788 | -0.175 | 0.0345 | 0.193 |
|  | Bayesian | (Intercept) | 0.264 | 0.141 | 0.355 | <0.001 |
|  |  | *N* | 0.00154 | -0.0103 | 0.0175 | 0.604 |
|  |  | Trait class (LH) | -0.0591 | -0.156 | 0.0455 | 0.290 |
|  |  | Trait class (O) | -0.11341 | -0.221 | 0.0177 | 0.105 |
|  |  | Analysis type (ANOVA) | 0.128 | -0.0283 | 0.286 | 0.110 |
|  |  | Analysis type (REML) | 0.0561 | -0.0336 | 0.174 | 0.184 |
| Other (SE) | ANOVA | (Intercept) | 0.274 | 0.100 | 0.436 | 0.00120 |
|  |  | *N* | 0.00193 | -0.0103 | 0.0177 | 0.599 |
|  |  | Trait class (LH) | 0.0579 | -0.0765 | 0.179 | 0.430 |
|  |  | Trait class (MO) | 0.108 | -0.0181 | 0.219 | 0.103 |
|  |  | Analysis type (Bayesian) | -0.137 | -0.291 | 0.0322 | 0.117 |
|  |  | Analysis type (REML) | -0.0503 | -0.193 | 0.0837 | 0.412 |
|  | REML | (Intercept) | 0.226 | 0.107 | 0.329 | 0.000200 |
|  |  | *N* | 0.00540 | -0.00960 | 0.0182 | 0.587 |
|  |  | Trait class (LH) | 0.0746 | -0.0749 | 0.181 | 0.424 |
|  |  | Trait class (MO) | 0.112 | -0.0162 | 0.216 | 0.0948 |
|  |  | Analysis type (ANOVA) | 0.0602 | -0.0799 | 0.198 | 0.397 |
|  |  | Analysis type (Bayesian) | -0.0706 | -0.169 | 0.0381 | 0.191 |
|  | Bayesian | (Intercept) | 0.140 | 0.0467 | 0.244 | 0.00600 |
|  |  | *N* | 0.00645 | -0.0106 | 0.0168 | 0.606 |
|  |  | Trait class (LH) | 0.0539 | -0.0854 | 0.174 | 0.447 |
|  |  | Trait class (MO) | 0.116 | -0.0187 | 0.217 | 0.106 |
|  |  | Analysis type (ANOVA) | 0.109 | -0.0308 | 0.283 | 0.104 |
|  |  | Analysis type (REML) | 0.0544 | -0.0363 | 0.170 | 0.191 |
| Life history (All data) | ANOVA | (Intercept) | 0.400 | 0.280 | 0.501 | <0.001 |
|  |  | *N* | -0.00307 | -0.0152 | 0.0120 | 0.758 |
|  |  | Trait class (MO) | 0.00698 | -0.0793 | 0.0992 | 0.769 |
|  |  | Trait class (O) | -0.0885 | -0.200 | 0.0371 | 0.201 |
|  |  | Analysis type (Bayesian) | -0.104 | -0.240 | 0.00908 | 0.0775 |
|  |  | Analysis type (REML) | -0.0961 | -0.194 | 0.0155 | 0.0805 |
|  | REML | (Intercept) | 0.301 | 0.215 | 0.379 | <0.001 |
|  |  | *N* | -0.00347 | -0.0158 | 0.0113 | 0.761 |
|  |  | Trait class (MO) | 0.0109 | -0.0759 | 0.0998 | 0.776 |
|  |  | Trait class (O) | -0.0741 | -0.202 | 0.0355 | 0.193 |
|  |  | Analysis type (ANOVA) | 0.115 | -0.00969 | 0.202 | 0.0810 |
|  |  | Analysis type (Bayesian) | -0.0187 | -0.108 | 0.0810 | 0.700 |
|  | Bayesian | (Intercept) | 0.286 | 0.188 | 0.372 | <0.001 |
|  |  | *N* | -0.00237 | -0.0161 | 0.0114 | 0.768 |
|  |  | Trait class (MO) | 0.000474 | -0.0740 | 0.0992 | 0.786 |
|  |  | Trait class (O) | -0.0841 | -0.191 | 0.0389 | 0.193 |
|  |  | Analysis type (ANOVA) | 0.0894 | -0.0120 | 0.240 | 0.0880 |
|  |  | Analysis type (REML) | 0.0281 | -0.0733 | 0.114 | 0.696 |
| Morphology (All data) | ANOVA | (Intercept) | 0.420 | 0.302 | 0.505 | <0.001 |
|  |  | *N* | -0.000900 | -0.0154 | 0.0118 | 0.781 |
|  |  | Trait class (LH) | -0.0101 | -0.106 | 0.0760 | 0.759 |
|  |  | Trait class (O) | -0.0862 | -0.202 | 0.0172 | 0.104 |
|  |  | Analysis type (Bayesian) | -0.107 | -0.236 | 0.0119 | 0.0792 |
|  |  | Analysis type (REML) | -0.110 | -0.199 | 0.0125 | 0.0880 |
|  | REML | (Intercept) | 0.320 | 0.262 | 0.361 | <0.001 |
|  |  | *N* | -0.00221 | -0.0157 | 0.0119 | 0.778 |
|  |  | Trait class (LH) | -0.0224 | -0.101 | 0.0750 | 0.787 |
|  |  | Trait class (O) | -0.0976 | -0.195 | 0.0190 | 0.101 |
|  |  | Analysis type (ANOVA) | 0.0855 | -0.0151 | 0.198 | 0.0952 |
|  |  | Analysis type (Bayesian) | -0.0287 | -0.112 | 0.0776 | 0.703 |
|  | Bayesian | (Intercept) | 0.291 | 0.196 | 0.391 | <0.001 |
|  |  | *N* | -0.00439 | -0.0156 | 0.0117 | 0.752 |
|  |  | Trait class (LH) | -0.0270 | -0.101 | 0.0737 | 0.784 |
|  |  | Trait class (O) | -0.0784 | -0.200 | 0.0136 | 0.0968 |
|  |  | Analysis type (ANOVA) | 0.121 | -0.0153 | 0.237 | 0.0850 |
|  |  | Analysis type (REML) | 0.0210 | -0.0738 | 0.115 | 0.708 |
| Other (All data) | ANOVA | (Intercept) | 0.287 | 0.175 | 0.450 | 0.000200 |
|  |  | *N* | -0.00131 | -0.0158 | 0.0116 | 0.776 |
|  |  | Trait class (LH) | 0.0901 | -0.0385 | 0.195 | 0.197 |
|  |  | Trait class (MO) | 0.104 | -0.0187 | 0.197 | 0.103 |
|  |  | Analysis type (Bayesian) | -0.127 | -0.237 | 0.0165 | 0.0816 |
|  |  | Analysis type (REML) | -0.115 | -0.200 | 0.0101 | 0.0816 |
|  | REML | (Intercept) | 0.225 | 0.117 | 0.319 | 0.000400 |
|  |  | *N* | -0.00262 | -0.0163 | 0.0111 | 0.774 |
|  |  | Trait class (LH) | 0.0880 | -0.0408 | 0.192 | 0.194 |
|  |  | Trait class (MO) | 0.0939 | -0.0162 | 0.199 | 0.0948 |
|  |  | Analysis type (ANOVA) | 0.0784 | -0.00815 | 0.201 | 0.0844 |
|  |  | Analysis type (Bayesian) | -0.0130 | -0.107 | 0.0791 | 0.700 |
|  | Bayesian | (Intercept) | 0.196 | 0.0989 | 0.316 | 0.000400 |
|  |  | *N* | -0.000120 | -0.0156 | 0.0114 | 0.754 |
|  |  | Trait class (LH) | 0.0978 | -0.0429 | 0.196 | 0.199 |
|  |  | Trait class (MO) | 0.0829 | -0.0139 | 0.200 | 0.104 |
|  |  | Analysis type (ANOVA) | 0.0986 | -0.0132 | 0.235 | 0.0832 |
|  |  | Analysis type (REML) | 0.0230 | -0.0776 | 0.108 | 0.698 |
