# Supplementary material for: Are heritability and selection related to population size in nature? Meta‐analysis and conservation implications
Source: Evol Appl. 2016 Apr 3;9(5):640–57. doi: 10.1111/eva.12375 (PMC4869407; doi:10.1111/eva.12375)

Appendix G (Fig. G1). Posterior modes of unweighted heritability values estimated using four different methods of analysis within each of three different trait classes. Error bars represent 95% HPD confidence intervals calculated using MCMCglmm. Sample sizes in each category are in brackets.


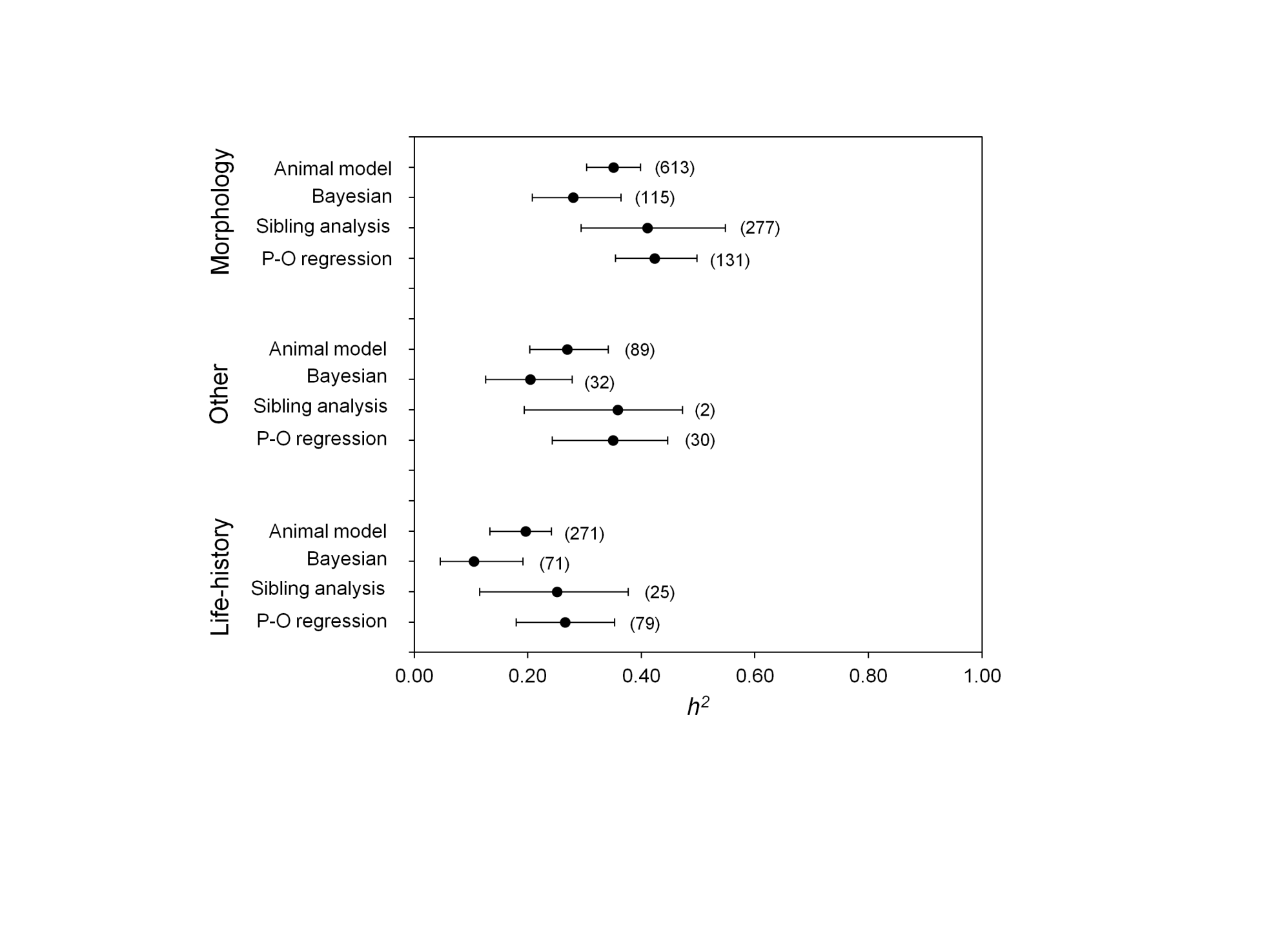


Appendix G (Fig. G2). Posterior modes of weighted heritability values estimated using three different methods of analysis within each of three different trait classes excluding *h^2^* estimates for bird populations. Error bars represent 95% HPD confidence intervals calculated using MCMCglmm. Sample sizes in each category are in brackets.


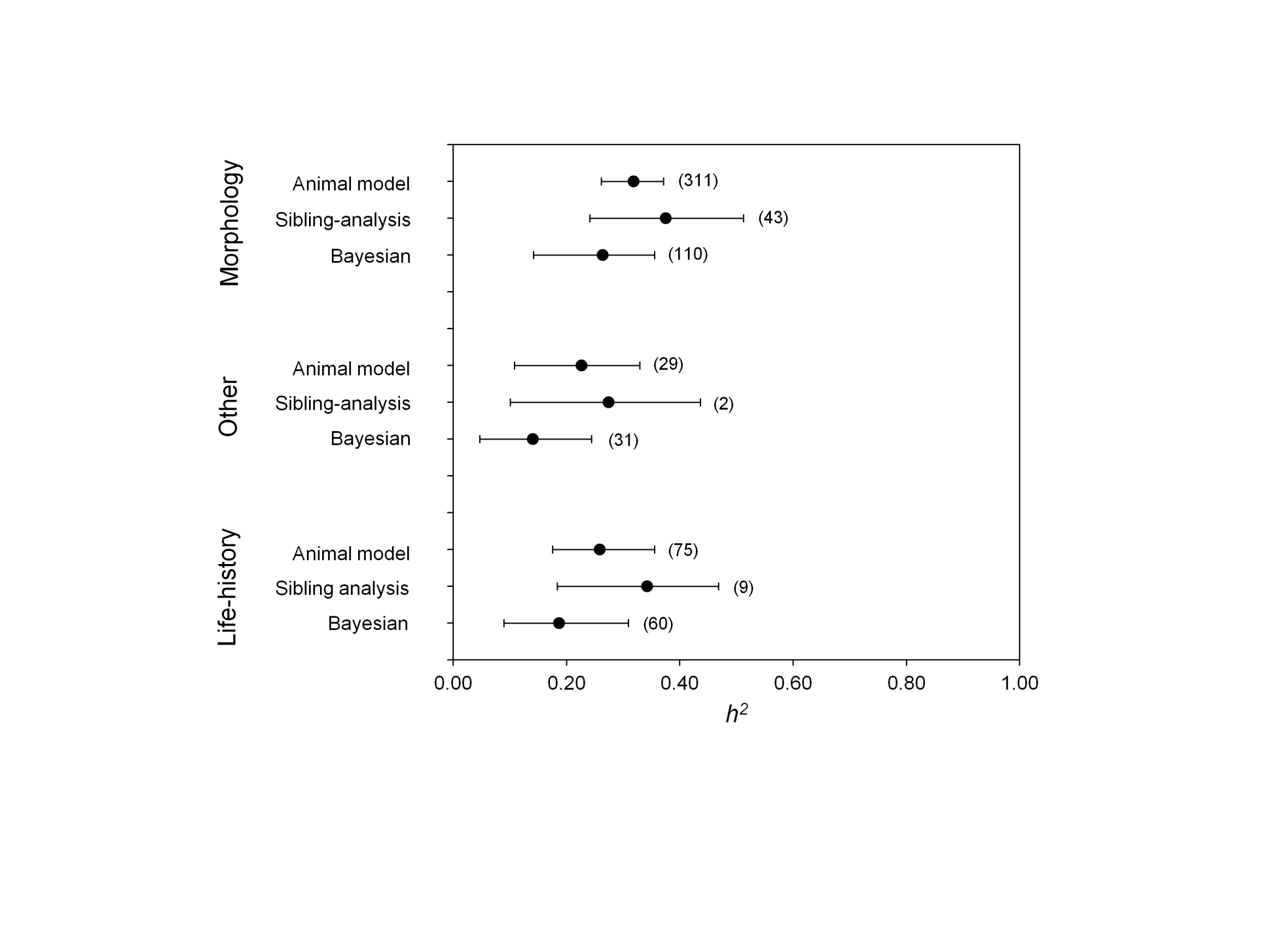


Appendix G (Fig. G3). Posterior modes of unweighted heritability values estimated using three different methods of analysis within each of three different trait classes excluding *h^2^* estimates for bird populations. Error bars represent 95% HPD confidence intervals calculated using MCMCglmm. Sample sizes in each category are in brackets.


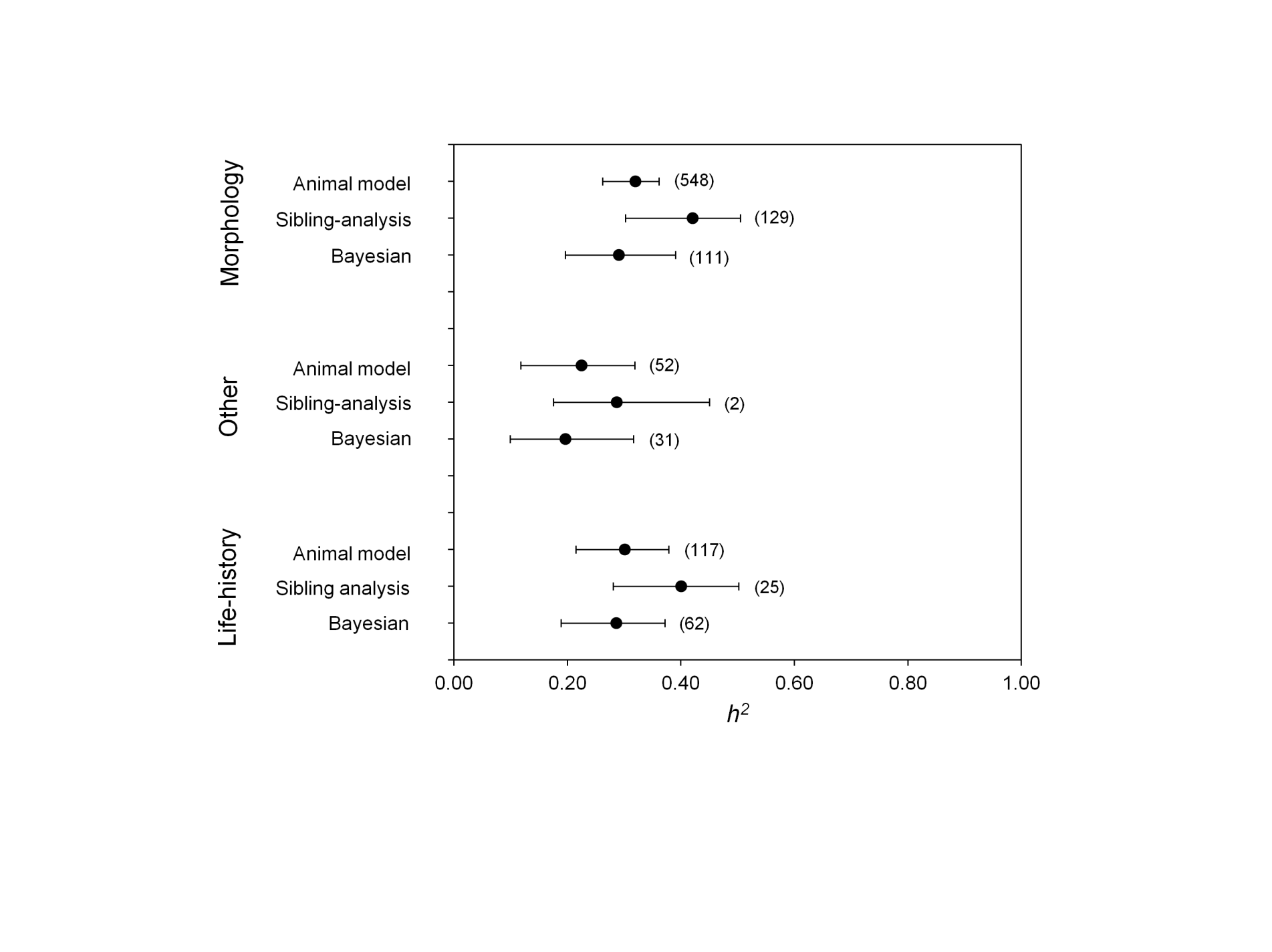

Supplement: Supplementary file 7 — Appendix S7. Posterior modes of unweighted h 2 values including all vertebrate data and weighted and unweighted h 2 values excluding bird data estimated using four different methods of analysis within each of three different trait classes. [file EVA-9-640-s007.docx]
