# Supplementary material for: Are heritability and selection related to population size in nature? Meta‐analysis and conservation implications
Source: Evol Appl. 2016 Apr 3;9(5):640–57. doi: 10.1111/eva.12375 (PMC4869407; doi:10.1111/eva.12375)
Supplement: Supplementary file 8 — Appendix S8. Results of models to investigate the effect of N on selection coefficient data using MCMCglmm. [file EVA-9-640-s008.docx]

Appendix H (Table H1). Results of unweighted models to investigate the effect of *N* on selection coefficient data using MCMCglmm. Models included selection coefficient data for bird populations.

| Selection coefficient | Intercept | Fixed effect | Posterior mode | l- 95% CI | u- 95% CI | *P_MCMC_* |
| --- | --- | --- | --- | --- | --- | --- |
| Linear gradient (all data) | Life history | (Intercept) | 0.129 | 0.0834 | 0.176 | <0.001 |
|  |  | *N* | 0.00276 | -0.0176 | 0.0192 | 0.904 |
|  |  | Trait class (MO) | -0.143 | -0.222 | -0.0668 | <0.001 |
|  |  | *N* × trait class (MO) | -0.0190 | -0.0453 | 0.00828 | 0.152 |
|  | Morphology | (Intercept) | 0.136 | 0.0798 | 0.173 | <0.001 |
|  |  | *N* | 0.0000656 | -0.0175 | 0.0202 | 0.907 |
|  |  | Trait class (L) | -0.131 | -0.221 | -0.0626 | <0.001 |
|  |  | *N* × trait class(L) | -0.0179 | -0.0455 | 0.00812 | 0.156 |
|  | Plants | (Intercept) | 0.142 | 0.0878 | 0.207 | <0.001 |
|  |  | *N* | -0.00998 | -0.0304 | 0.00564 | 0.180 |
|  |  | Taxa(V) | -0.120 | -0.193 | -0.0473 | 0.00100 |
|  |  | *N* × taxa(V) | 0.00525 | -0.0236 | 0.0280 | 0.857 |
|  | Vertebrates | (Intercept) | 0.0263 | -0.0163 | 0.0664 | 0.240 |
|  |  | *N* | -0.0120 | -0.0280 | 0.00809 | 0.302 |
|  |  | Taxa(P) | 0.113 | 0.0512 | 0.193 | 0.00175 |
|  |  | *N* × taxa(P) | -0.00115 | -0.0284 | 0.0220 | 0.838 |
| Linear differential (all data) | Life history | (Intercept) | 0.0157 | -0.155 | 0.190 | 0.904 |
|  |  | *N* | -0.0271 | -0.0729 | 0.0220 | 0.284 |
|  |  | Trait class (MO) | 0.156 | -0.0457 | 0.330 | 0.123 |
|  |  | *N* × trait class (MO) | 0.0468 | -0.0182 | 0.0852 | 0.210 |
|  | Morphology | (Intercept) | 0.155 | 0.0765 | 0.242 | <0.001 |
|  |  | *N* | 0.00996 | -0.0135 | 0.0299 | 0.498 |
|  |  | Trait class (L) | -0.165 | -0.344 | 0.0408 | 0.128 |
|  |  | *N* × trait class(L) | -0.0296 | -0.0854 | 0.0167 | 0.203 |
|  | Plants | (Intercept) | 0.118 | -0.00814 | 0.249 | 0.0645 |
|  |  | *N* | -0.00978 | -0.0401 | 0.0171 | 0.424 |
|  |  | Taxa(V) | -0.0397 | -0.171 | 0.138 | 0.806 |
|  |  | *N* × taxa(V) | 0.00957 | -0.0245 | 0.0516 | 0.499 |
|  | Vertebrates | (Intercept) | 0.109 | 0.0170 | 0.188 | 0.0185 |
|  |  | *N* | 0.000822 | -0.0245 | 0.0258 | 0.910 |
|  |  | Taxa(P) | 0.0133 | -0.140 | 0.166 | 0.829 |
|  |  | *N* × taxa(P) | -0.00974 | -0.0506 | 0.0259 | 0.502 |
| Quadratic gradient (all data) | Life history | (Intercept) | 0.0722 | -0.120 | 0.195 | 0.550 |
|  |  | *N* | 0.0336 | -0.0290 | 0.0799 | 0.266 |
|  |  | Trait class (MO) | -0.0997 | -0.266 | 0.0908 | 0.326 |
|  |  | *N* × trait class (MO) | -0.0110 | -0.0905 | 0.0513 | 0.624 |
|  | Morphology | (Intercept) | -0.0409 | -0.125 | 0.0331 | 0.312 |
|  |  | *N* | 0.0145 | -0.0331 | 0.0596 | 0.590 |
|  |  | Trait class (LH) | 0.106 | -0.0894 | 0.265 | 0.318 |
|  |  | *N* × trait class (LH) | 0.0220 | -0.0499 | 0.0896 | 0.615 |
|  | Plants | (Intercept) | -0.0224 | -0.134 | 0.0703 | 0.487 |
|  |  | *N* | 0.0262 | -0.00356 | 0.0648 | 0.0825 |
|  |  | Taxa (V) | 0.0233 | -0.128 | 0.216 | 0.596 |
|  |  | *N* × taxa (V) | 0.00115 | -0.0759 | 0.0853 | 0.931 |
|  | Vertebrates | (Intercept) | 0.00844 | -0.129 | 0.149 | 0.885 |
|  |  | *N* | 0.0379 | -0.0423 | 0.108 | 0.390 |
|  |  | Taxa (P) | -0.0370 | -0.223 | 0.124 | 0.584 |
|  |  | *N* × taxa (P) | -0.00666 | -0.0863 | 0.0747 | 0.925 |
| Quadratic differential (all data) |  | Intercept | -0.0855 | -0.226 | 0.0876 | 0.380 |
|  |  | *N* | 0.00136 | -0.0657 | 0.0783 | 0.926 |

Appendix H (Table H2). Results of meta-analysis and unweighted models to investigate the effect of *N* on selection coefficient data using MCMCglmm. Models excluded selection coefficient data for bird populations.

| Selection coefficient | Intercept | Fixed effect | Posterior mode | l-95% CI | u-95% CI | *P_MCMC_* |
| --- | --- | --- | --- | --- | --- | --- |
| Linear gradient (SE) | Life history | (Intercept) | -0.0409 | -0.118 | 0.0517 | 0.444 |
|  |  | *N* | -0.0294 | -0.0615 | 0.0114 | 0.164 |
|  |  | Trait class (MO) | 0.158 | 0.0561 | 0.273 | 0.00460 |
|  |  | *N* × trait class (MO) | 0.0147 | -0.0296 | 0.0602 | 0.458 |
|  | Morphology | (Intercept) | 0.123 | 0.0646 | 0.203 | <0.001 |
|  |  | *N* | -0.0132 | -0.0344 | 0.0160 | 0.474 |
|  |  | Trait class (LH) | -0.156 | -0.280 | -0.0590 | 0.00360 |
|  |  | *N* × trait class (LH) | -0.0131 | -0.0615 | 0.0274 | 0.455 |
|  | Plants | (Intercept) | 0.119 | 0.0287 | 0.186 | 0.00625 |
|  |  | *N* | -0.0151 | -0.0355 | 0.0113 | 0.295 |
|  |  | Taxa (V) | -0.0833 | -0.197 | 0.0258 | 0.118 |
|  |  | *N* × taxa (V) | 0.00305 | -0.0414 | 0.0460 | 0.934 |
|  | Vertebrates | (Intercept) | 0.0252 | -0.0552 | 0.0992 | 0.540 |
|  |  | *N* | -0.0177 | -0.0474 | 0.0262 | 0.582 |
|  |  | Taxa (P) | 0.103 | -0.0211 | 0.197 | 0.124 |
|  |  | *N* × taxa (P) | -0.00365 | -0.0467 | 0.0405 | 0.928 |
| Linear gradient (all data) | Life history | (Intercept) | 0.0280 | -0.0670 | 0.118 | 0.568 |
|  |  | *N* | -0.0198 | -0.0485 | 0.00728 | 0.151 |
|  |  | Trait class (MO) | 0.128 | 0.0112 | 0.238 | 0.0232 |
|  |  | *N* × trait class (MO) | 0.0197 | -0.0163 | 0.0578 | 0.270 |
|  | Morphology | (Intercept) | 0.155 | 0.0871 | 0.222 | <0.001 |
|  |  | *N* | -0.00178 | -0.0242 | 0.0228 | 0.984 |
|  |  | Trait class (L) | -0.126 | -0.239 | -0.0135 | 0.0254 |
|  |  | *N* × trait class(L) | -0.0179 | -0.0566 | 0.0180 | 0.270 |
|  | Plants | (Intercept) | 0.155 | 0.0816 | 0.222 | <0.001 |
|  |  | *N* | -0.0117 | -0.0339 | 0.00559 | 0.160 |
|  |  | Taxa(V) | -0.0880 | -0.204 | -0.000240 | 0.0505 |
|  |  | *N* × taxa(V) | 0.0233 | -0.0134 | 0.0726 | 0.215 |
|  | Vertebrates | (Intercept) | 0.0579 | -0.0171 | 0.127 | 0.158 |
|  |  | *N* | 0.00525 | -0.0262 | 0.0487 | 0.500 |
|  |  | Taxa(P) | 0.0971 | -0.00214 | 0.201 | 0.0582 |
|  |  | *N* × taxa(P) | -0.0224 | -0.0710 | 0.0143 | 0.213 |
| Linear differential (SE) | LH | (Intercept) | 0.0459 | -0.267 | 0.385 | 0.724 |
|  |  | *N* | -0.00908 | -0.119 | 0.0922 | 0.742 |
|  |  | Trait class (MO) | 0.248 | -0.208 | 0.684 | 0.249 |
|  |  | *N* × trait class (MO) | 0.00660 | -0.132 | 0.105 | 0.878 |
|  | MO | (Intercept) | 0.319 | -0.0252 | 0.575 | 0.0794 |
|  |  | *N* | -0.0321 | -0.0754 | 0.0279 | 0.345 |
|  |  | Trait class (LH) | -0.216 | -0.653 | 0.236 | 0.253 |
|  |  | *N* × trait class (LH) | 0.00160 | -0.109 | 0.125 | 0.866 |
|  | Plants | (Intercept) | NA | NA | NA | NA |
|  |  | *N* | NA | NA | NA | NA |
|  |  | Taxa (V) | NA | NA | NA | NA |
|  |  | *N* × taxa (V) | NA | NA | NA | NA |
|  | Vertebrates | (Intercept) | NA | NA | NA | NA |
|  |  | *N* | NA | NA | NA | NA |
|  |  | Taxa (P) | NA | NA | NA | NA |
|  |  | *N* × taxa (P) | NA | NA | NA | NA |
| Linear differential (all data) | LH | (Intercept) | -0.0220 | -0.271 | 0.214 | 0.743 |
|  |  | *N* | -0.0242 | -0.0770 | 0.0433 | 0.564 |
|  |  | Trait class (MO) | 0.203 | -0.0800 | 0.472 | 0.140 |
|  |  | *N* × trait class (MO) | 0.0317 | -0.0350 | 0.0982 | 0.314 |
|  | MO | (Intercept) | 0.190 | 0.0367 | 0.314 | 0.0158 |
|  |  | *N* | 0.0189 | -0.0121 | 0.0458 | 0.279 |
|  |  | Trait class (L) | -0.230 | -0.491 | 0.0690 | 0.144 |
|  |  | *N* × trait class(L) | -0.0327 | -0.100 | 0.0315 | 0.292 |
|  | Plants | (Intercept) | 0.0993 | -0.00978 | 0.244 | 0.067 |
|  |  | *N* | -0.0125 | -0.0416 | 0.0165 | 0.446 |
|  |  | Taxa(V) | -0.0361 | -0.170 | 0.141 | 0.819 |
|  |  | *N* × taxa(V) | 0.0111 | -0.0270 | 0.0492 | 0.523 |
|  | Vertebrates | (Intercept) | 0.0889 | 0.0189 | 0.184 | 0.0175 |
|  |  | *N* | 0.00142 | -0.0244 | 0.0259 | 0.893 |
|  |  | Taxa(P) | 0.000810 | -0.132 | 0.172 | 0.816 |
|  |  | *N* × taxa(P) | -0.00974 | -0.0541 | 0.0236 | 0.490 |
| Quadratic gradient (SE) | LH | (Intercept) | 0.150 | -0.0313 | 0.275 | 0.100 |
|  |  | *N* | 0.111 | 0.0460 | 0.176 | 0.00300 |
|  |  | Trait class (MO) | -0.121 | -0.291 | 0.0265 | 0.0968 |
|  |  | *N* × trait class (MO) | -0.100 | -0.172 | -0.0326 | 0.00680 |
|  | MO | (Intercept) | -0.00868 | -0.0508 | 0.0364 | 0.779 |
|  |  | *N* | 0.0107 | -0.0160 | 0.0348 | 0.440 |
|  |  | Trait class (LH) | 0.138 | -0.0277 | 0.299 | 0.101 |
|  |  | *N* × trait class (LH) | 0.0978 | 0.0294 | 0.172 | 0.00620 |
|  | Plants | (Intercept) | 0.000531 | -0.113 | 0.128 | 0.849 |
|  |  | *N* | 0.0691 | 0.0260 | 0.127 | 0.00300 |
|  |  | Taxa (V) | -0.0350 | -0.173 | 0.124 | 0.705 |
|  |  | *N* × taxa (V) | -0.0344 | -0.0982 | 0.0385 | 0.321 |
|  | Vertebrates | (Intercept) | -0.0159 | -0.104 | 0.0737 | 0.714 |
|  |  | *N* | 0.0334 | -0.0106 | 0.0929 | 0.116 |
|  |  | Taxa (P) | 0.0563 | -0.127 | 0.170 | 0.707 |
|  |  | *N* × taxa (P) | 0.0431 | -0.0315 | 0.103 | 0.301 |
| Quadratic gradient (all data) | LH | (Intercept) | 0.0722 | -0.120 | 0.195 | 0.550 |
|  |  | *N* | 0.0336 | -0.0290 | 0.0799 | 0.266 |
|  |  | Trait class (MO) | -0.0997 | -0.266 | 0.0909 | 0.326 |
|  |  | *N* × trait class (MO) | -0.0110 | -0.0905 | 0.0513 | 0.624 |
|  | MO | (Intercept) | -0.0409 | -0.125 | 0.0331 | 0.312 |
|  |  | *N* | 0.0145 | -0.0331 | 0.0596 | 0.590 |
|  |  | Trait class (L) | 0.106 | -0.0894 | 0.265 | 0.318 |
|  |  | *N* × trait class(L) | 0.0220 | -0.0499 | 0.0896 | 0.615 |
|  | Plants | (Intercept) | -0.0224 | -0.134 | 0.0703 | 0.487 |
|  |  | *N* | 0.0262 | -0.00356 | 0.0648 | 0.0825 |
|  |  | Taxa(V) | 0.0233 | -0.128 | 0.216 | 0.596 |
|  |  | *N* × taxa(V) | 0.00115 | -0.0759 | 0.0853 | 0.931 |
|  | Vertebrates | (Intercept) | 0.00844 | -0.129 | 0.149 | 0.885 |
|  |  | *N* | 0.0379 | -0.0423 | 0.108 | 0.390 |
|  |  | Taxa(P) | -0.0370 | -0.223 | 0.124 | 0.584 |
|  |  | *N* × taxa(P) | -0.00666 | -0.0863 | 0.0747 | 0.925 |
| Quadratic differential (SE) |  | Intercept | -0.0334 | -0.231 | 0.201 | 0.850 |
|  |  | *N* | 0.0324 | -0.0589 | 0.131 | 0.502 |
| Quadratic differential (all data) data) |  | Intercept | -0.119 | -0.390 | 0.147 | 0.361 |
|  |  | *N* | -0.0165 | -0.1103 | 0.129 | 0.951 |
