# Supplementary material for: Are heritability and selection related to population size in nature? Meta‐analysis and conservation implications
Source: Evol Appl. 2016 Apr 3;9(5):640–57. doi: 10.1111/eva.12375 (PMC4869407; doi:10.1111/eva.12375)

Appendix I (Fig. I1): Posterior modes of the a) weighted and b) unweighted magnitude of linear selection differential values in three different *N* bins. The magnitude of selection was calculated using the folded binomial distribution. Error bars represent 95% HPD confidence intervals calculated using MCMCglmm. Sample sizes in each *N* bin are in brackets.


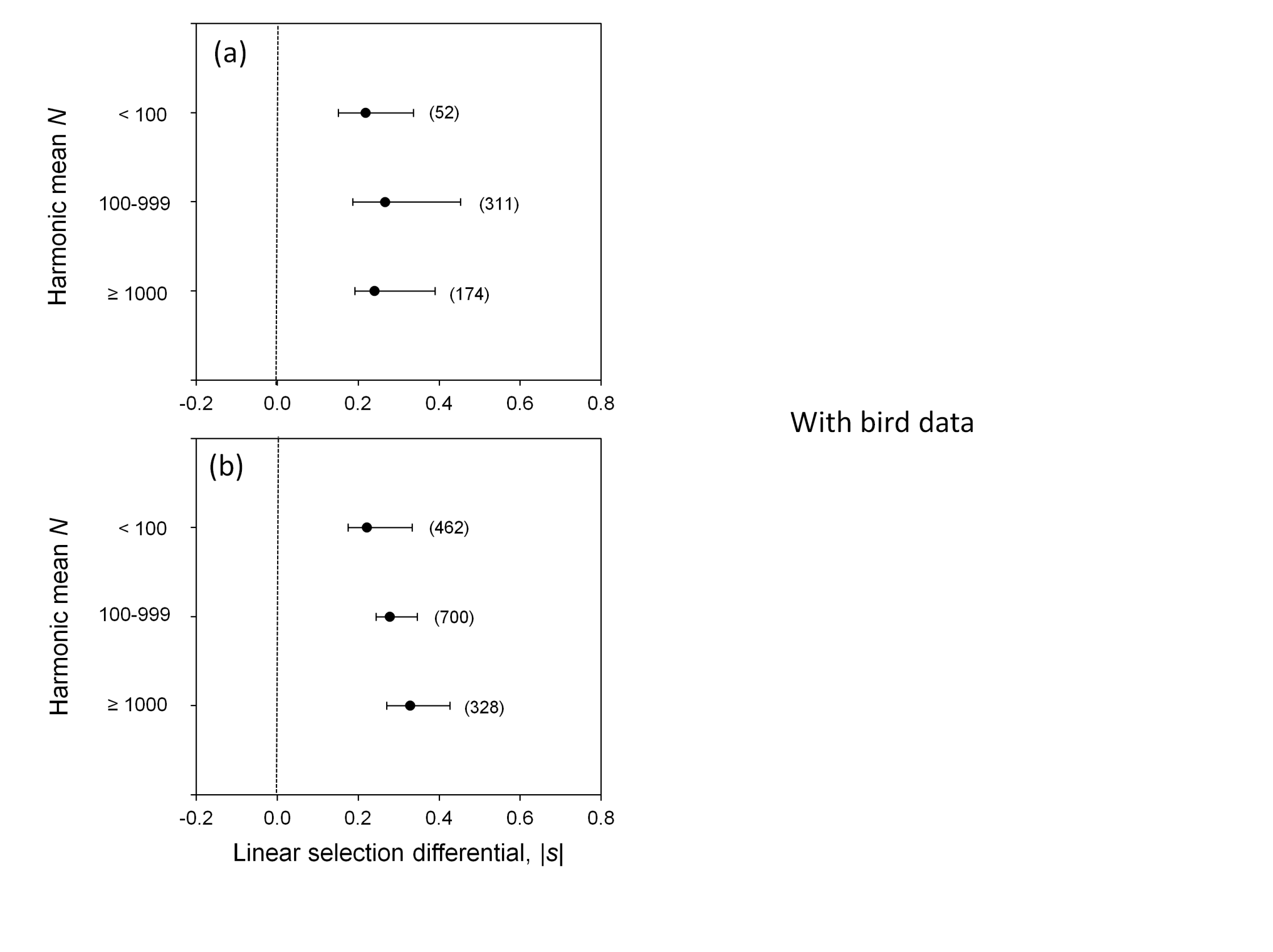


Appendix I (Fig. I2): Posterior modes of the unweighted magnitude of linear selection gradients in four different *N* bins where the largest bin consisted of a) *N* ≥ 1000 individuals or b) ≥ 4000 individuals. The magnitude of selection was calculated using the folded binomial distribution. Error bars represent 95% HPD confidence intervals calculated using MCMCglmm. Sample sizes in each *N* bin are in brackets.


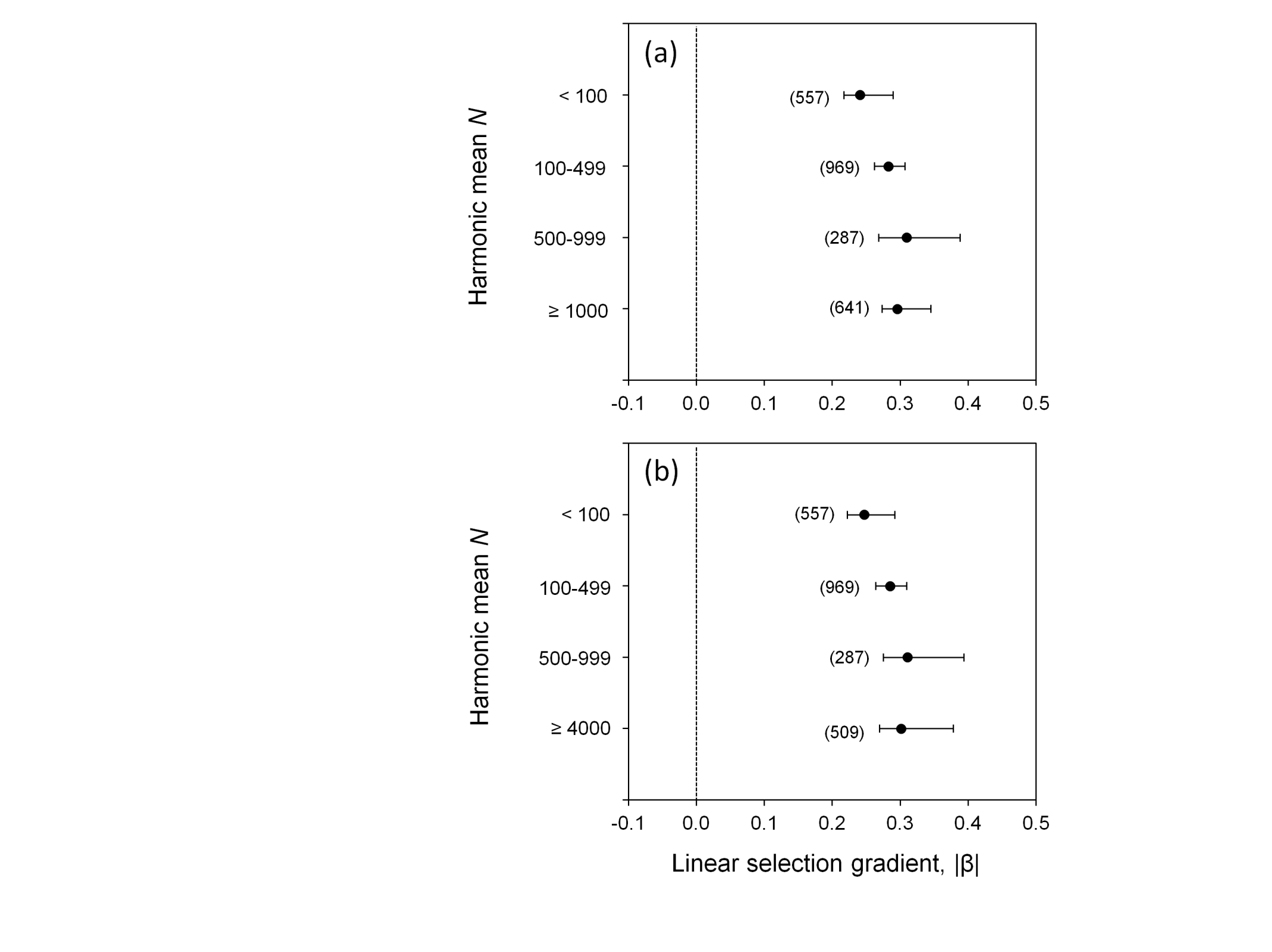


Appendix I (Fig. I3): Posterior modes of the unweighted magnitude of linear selection gradients for a) morphological and life history traits, and b) plants and vertebrates in each of four *N* bins. The magnitude of selection was calculated using the folded binomial distribution. Error bars represent 95% HPD confidence intervals calculated using MCMCglmm. Sample sizes in each *N* bin are in brackets.


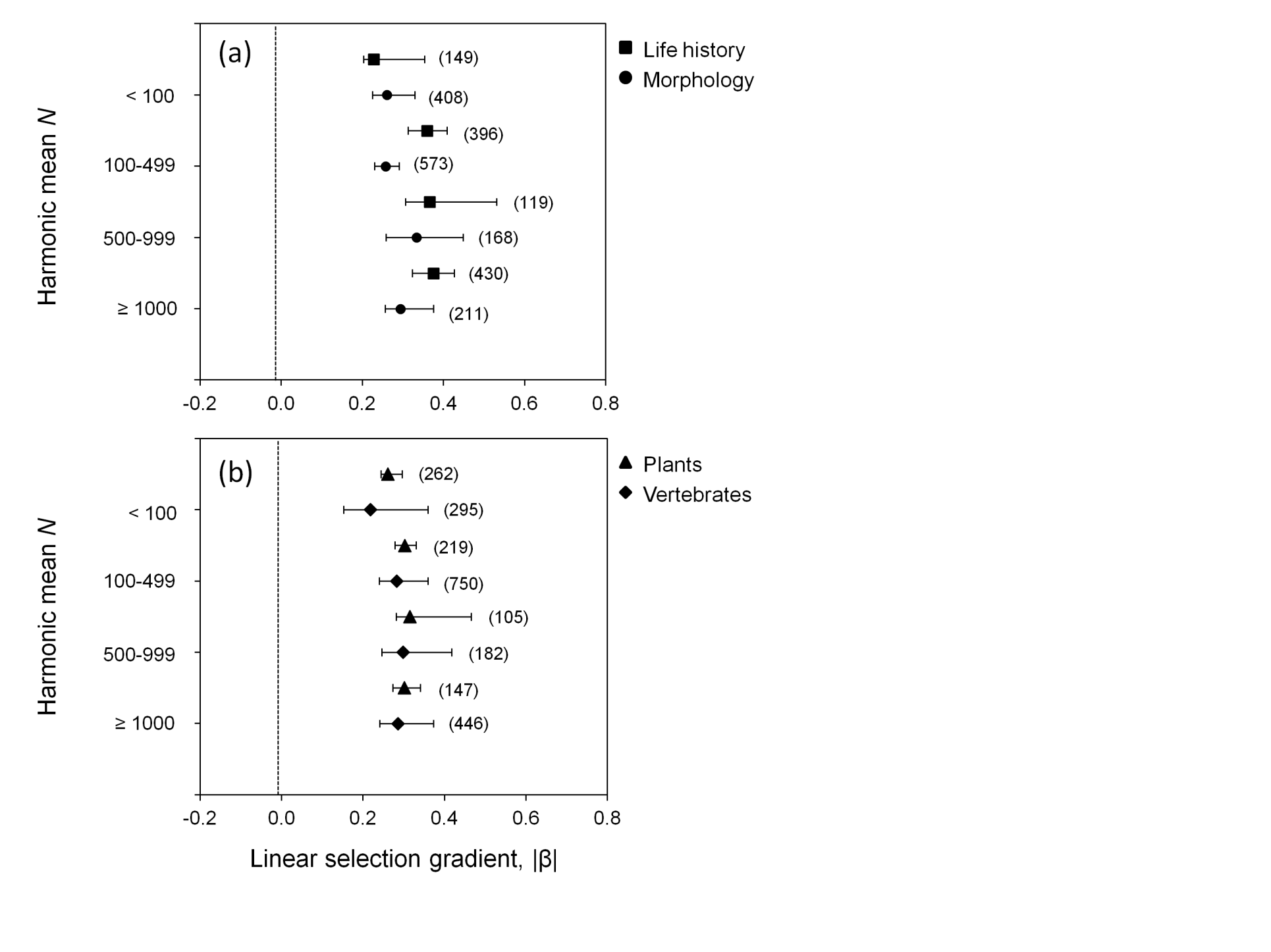


Appendix I (Fig. I4): Posterior modes of the weighted magnitude of linear selection gradients in four different *N* bins excluding data for wild bird populations where the largest bin consisted of a) *N* ≥ 1000 individuals or b) ≥ 4000 individuals. The magnitude of selection was calculated using the folded binomial distribution. Error bars represent 95% HPD confidence intervals calculated using MCMCglmm. Sample sizes in each *N* bin are in brackets.


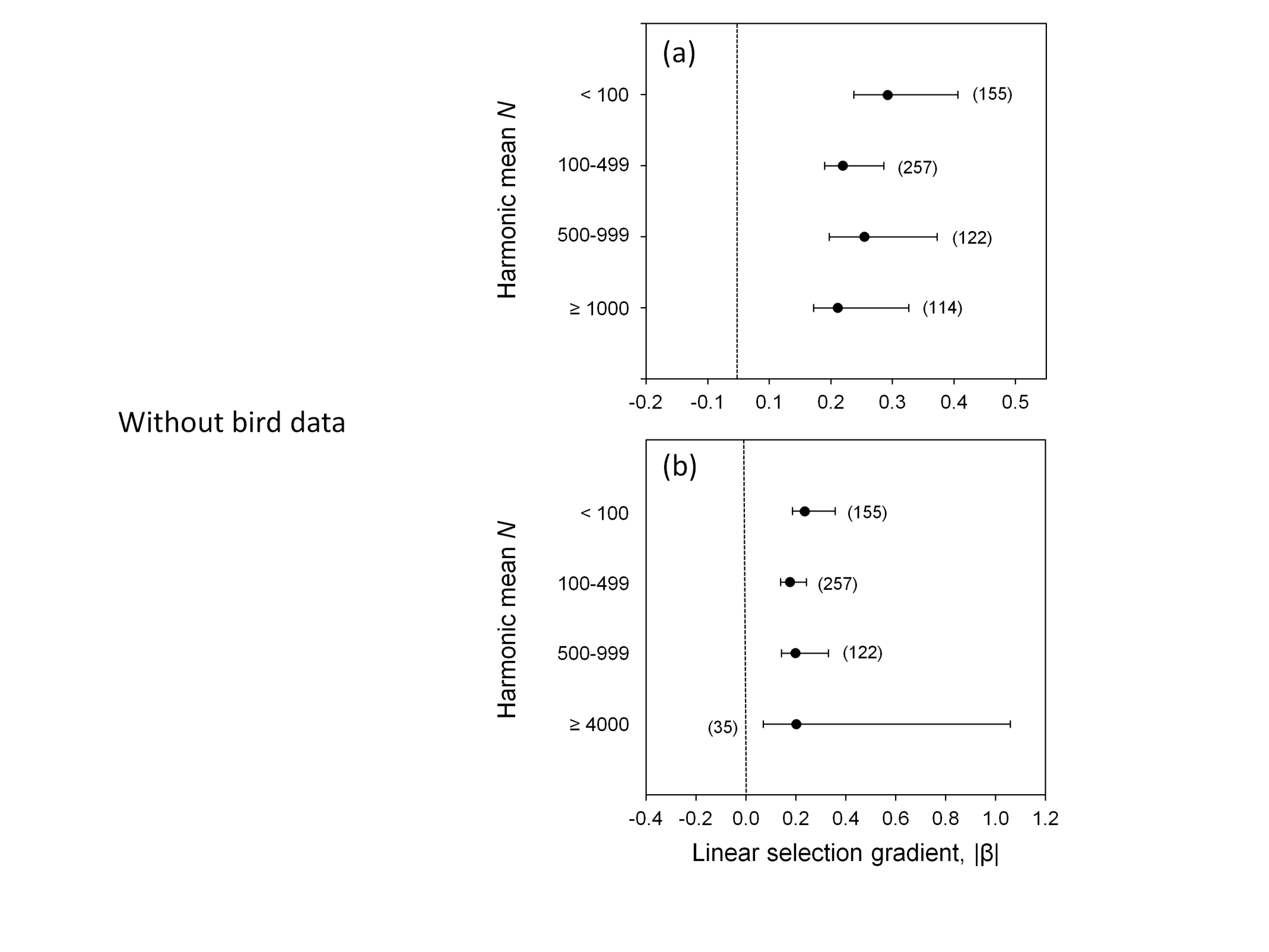


Appendix I (Fig. I5): Posterior modes of the unweighted magnitude of linear selection gradients in four different *N* bins excluding data for wild bird populations where the largest bin consisted of a) *N* ≥ 1000 individuals or b) ≥ 4000 individuals. The magnitude of selection was calculated using the folded binomial distribution. Error bars represent 95% HPD confidence intervals calculated using MCMCglmm. Sample sizes in each *N* bin are in brackets.


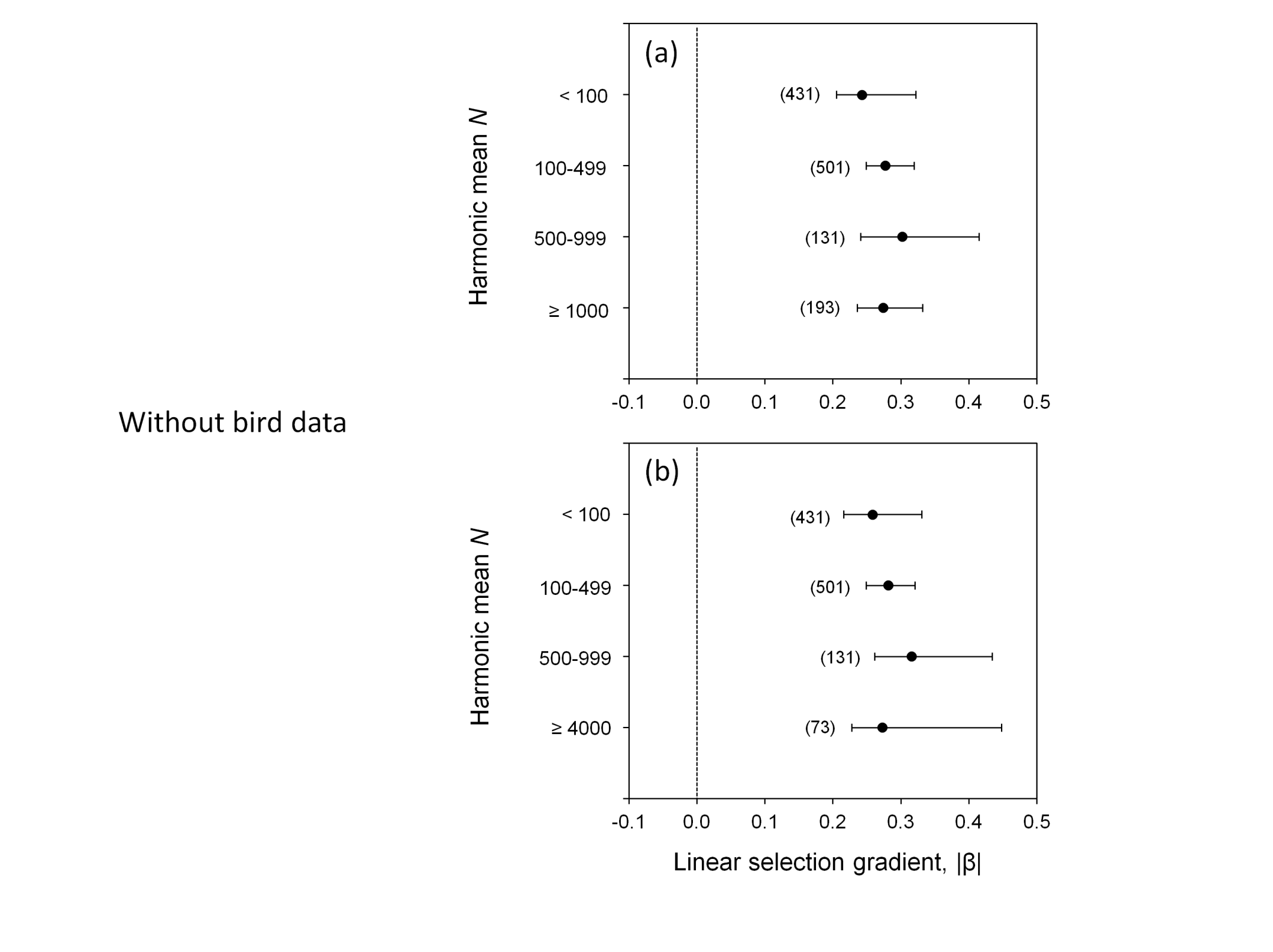


Appendix I (Fig. I6): Posterior modes of the (a) weighted and (b) unweighted magnitude of linear selection gradients for morphological and life history traits in each of four *N* bins excluding data for wild bird populations. The magnitude of selection was calculated using the folded binomial distribution. Error bars represent 95% HPD confidence intervals calculated using MCMCglmm. Sample sizes in each *N* bin are in brackets.


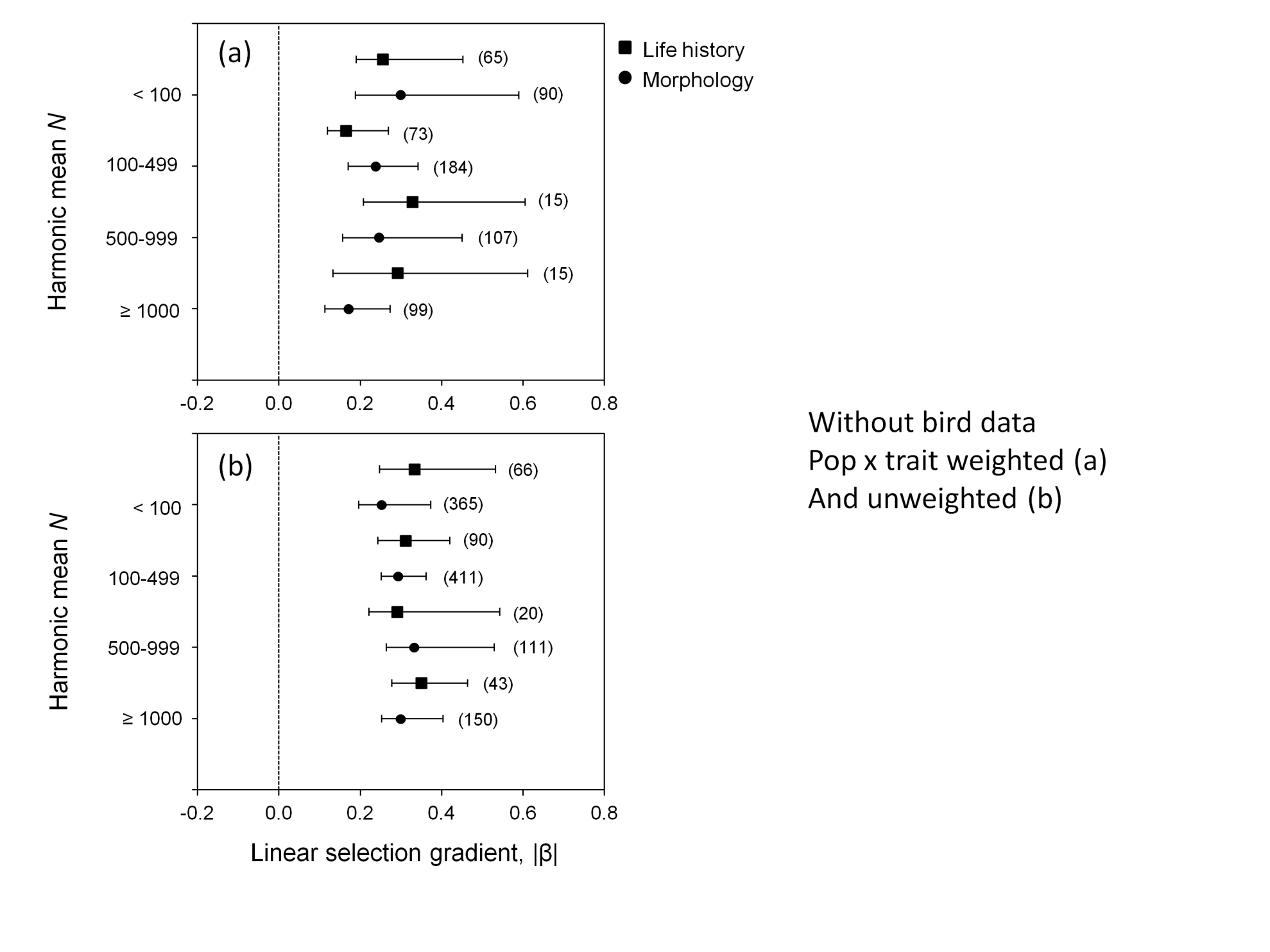

Supplement: Supplementary file 9 — Appendix S9. Posterior modes of weighted and unweighted values for all vertebrate data and excluding bird data for the magnitude of two types of selection coefficients in relation to N bins. [file EVA-9-640-s009.docx]
